# Crosstalk between copper homeostasis and cuproptosis reveals a lncRNA signature to prognosis prediction, immunotherapy personalization, and agent selection for patients with lung adenocarcinoma

**DOI:** 10.18632/aging.205281

**Published:** 2023-11-26

**Authors:** Chao Ma, Zhuoyu Gu, Weizheng Ding, Feng Li, Yang Yang

**Affiliations:** 1 Department of Thoracic Surgery, The First Affiliated Hospital of Zhengzhou University, Zhengzhou, China

**Keywords:** lung adenocarcinoma, lncRNA signature, copper homeostasis, cuproptosis, prognosis, immunotherapy, drug prediction

## Abstract

Background: Copper homeostasis and cuproptosis play critical roles in various biological processes of cancer; however, whether they can impact the prognosis of lung adenocarcinoma (LUAD) remain to be fully elucidated. We aimed to adopt these concepts to create and validate a lncRNA signature for LUAD prognostic prediction.

Methods: For this study, the TCGA-LUAD dataset was used as the training cohort, and multiple datasets from the GEO database were pooled as the validation cohort. Copper homeostasis and cuproptosis regulated genes were obtained from published studies, and various statistical methods, including Kaplan-Meier (KM), Cox, and LASSO, were used to train our gene signature CoCuLncSig. We utilized KM analysis, COX analysis, receiver operating characteristic analysis, time-dependent AUC analysis, principal component analysis, and nomogram predictor analysis in our validation process. We also compared CoCuLncSig with previous studies. We performed analyses using R software to evaluate CoCuLncSig's immunotherapeutic ability, focusing on eight immune algorithms, TMB, and TIDE. Additionally, we investigated potential drugs that could be effective in treating patients with high-risk scores. Additionally qRT-PCR examined the expression patterns of CoCuLncSig lncRNAs, and the ability of CoCuLncSig in pan-cancer was also assessed.

Results: CoCuLncSig containing eight lncRNAs was trained and showed strong predictive ability in the validation cohort. Compared with previous similar studies, CoCuLncSig had more prognostic ability advantages. CoCuLncSig was closely related to the immune status of LUAD, and its tight relationship with checkpoints IL10, IL2, CD40LG, SELP, BTLA, and CD28 may be the key to its potential immunotherapeutic ability. For the high CoCuLncSig score population, we found 16 drug candidates, among which epothilone-b and gemcitabine may have the most potential. The pan-cancer analysis found that CoCuLncSig was a risk factor in multiple cancers. Additionally, we discovered that some of the CoCuLncSig lncRNAs could play crucial roles in specific cancer types.

Conclusion: The current study established a powerful prognostic CoCuLncSig signature for LUAD that was also valid for most pan-cancers. This signature could serve as a potential target for immunotherapy and might help the more efficient application of drugs to specific populations.

## INTRODUCTION

Lung cancer is the leading cancer diagnosis and cause of cancer-related deaths worldwide [1, 2]. In 2020, there were 2.2 million new cases of lung cancer and 1.8 million deaths attributed to this disease, accounting for 18% of all cancer-related deaths [1, 2]. Unfortunately, lung cancer-related deaths are projected to increase globally to approximately 3 million annually by 2035 due to the high prevalence of tobacco use and aging populations [1, 2]. The most common type of lung cancer is lung adenocarcinoma (LUAD) [1, 2]. Treatment strategies for LUAD can be divided into five categories: surgery, chemotherapy, radiotherapy, targeted therapy, and immunotherapy [3–5]. Despite ongoing updates to clinical management strategies for LUAD, issues such as a low early diagnosis rate and unsatisfactory long-term patient survival persist [3–5]. Therefore, it is imperative to identify a new clinical model that can precisely diagnose and prognose LUAD, delve deeper into the molecular mechanisms underlying its development, and generate novel ideas for targeted therapies.

Cells tightly regulate their copper homeostasis through a network of copper-dependent proteins, which maintain the intracellular copper content within specific ranges [6]. Maintaining copper homeostasis is essential to avoid the adverse effects of both copper deficiency and copper overload [6]. An imbalance in copper levels in the body has been correlated with several diseases, including cancer [6]. Copper plays a crucial role in cell signaling and contributes to cancer development by promoting cell proliferation, angiogenesis, and metastasis [6]. Studies have found elevated levels of copper in lung cancer tissue, and research suggests that high serum copper levels in patients with lung cancer are linked to tumor stage and disease progression [7]. Tsvetkov et al. discovered a novel form of regulated cell death, called cuproptosis, which is induced by intracellular copper [6, 8]. This unique pathway of cell death is distinguishable from established cell death mechanisms by the aggregation of lipidated mitochondrial enzymes and the loss of Fe-S protein [6, 8]. The discovery of cuproptosis opens up new possibilities for potential applications in cancer therapy. Evidence suggests that copper complexes can be targeted for therapeutic use in cancer treatment [9]. However, the precise mechanism of cuproptosis remains unclear, and its association with LUAD requires further investigation. Considering the critical roles of copper homeostasis and cuproptosis in cancer progression, the corresponding related gene signatures are expected to bring new insights into LUAD clinical treatment and more clues to reveal the underlying molecular mechanisms.

lncRNAs are RNA molecules that are longer than 200 nucleotides and do not provide instructions for making proteins [10, 11]. They are known to have significant roles in a variety of biological processes such as gene regulation, chromatin modification, and epigenetic regulation [10, 11]. Numerous studies have investigated the possibility of using lncRNAs as indicators of lung cancer in order to diagnose and forecast the disease [10, 11]. These studies have discovered many lncRNAs that are abnormal in lung cancer and show promise as diagnostic and prognostic biomarkers. For example, lung cancer tissues exhibit elevated levels of HOTAIR [12] and MALAT1 [13], which can facilitate the growth, invasion, and metastasis of lung cancer. Furthermore, a high degree of HOTAIR and MALAT1 expression is linked to unfavorable prognoses in individuals with lung cancer [12, 13]. Considering their potential as indicators of lung cancer, lncRNAs could be utilized as therapeutic interventions for LUAD. Further research on lncRNAs is needed to investigate their potential.

The goal of this research is to develop a prognostic signature for copper homeostasis and cuproptosis regulated lncRNAs in LUAD. The study involved validating the prognostic potential of the signature in a large independent group and comparing its effectiveness to similar previous studies. Using qRT-PCR, we also confirmed the differential expression of signature lncRNAs in normal and tumor lung tissues. Moreover, the study explored the potential of immunotherapy and identified certain checkpoints (such as IL10, IL2, CD40LG, SELP, BTLA, and CD28) that could serve as indicators for the signature, and potentially be used in immunotherapy for patients with LUAD. The research identified several agents that could be possible treatment options for high-risk patients and evaluated the potential of the signature in pan-cancer.

## MATERIALS AND METHODS

### Exploring datasets for this study and data preprocessing

In the present study, we utilized expression data and clinical characterization of patients with LUAD from the TCGA-LUAD project, which was acquired through the Xena Hub online portal (https://xenabrowser.net/) and served as the training cohort. To validate our results, we utilized the Gene Expression Omnibus (GEO, https://www.ncbi.nlm.nih.gov/geo/) to gather validation data. Our search on GEO was tailored to identify a dataset related to “lung adenocarcinoma”, where we filtered out any results that did not contain expression and survival data to create our candidate dataset. We opted for GSE29013, GSE30219, GSE31210, GSE37745, and GSE50081 datasets from GEO. It is essential to highlight that these GEO datasets underwent preprocessing before being used. To carry out preprocessing, we utilized the R package “inSilicoMerging” [14] to merge them, and we eliminated batch effects using the approach established by Johnson et al. [15]. The preprocessed GEO data was utilized as the validation cohort.

### Consensus clustering for clusters that identified by copper homeostasis and cuproptosis correlated genes (CoCu clusters)

We selected 11 copper homeostasis-regulated genes [6], STEAP1, SLC31A1, CCS, SOD1, ATOX1, ATP7A, ATP7B, COX17, COX11, SCO1, and MT-CO1, and 10 cuproptosis-regulated genes [8], FDX1, LIAS, PDHA1, PDHB, MTF1, LIPT1, DLD, DLAT, GLS, and CDKN2A from previous studies. Applying the Pearson test to the copper homeostasis-regulated genes in the LUAD population and setting the threshold as |coefficient| > 0.6 to yield copper homeostasis-correlated genes. The same method and threshold were applied to the cuproptosis-regulated genes and outputted cuproptosis-correlated genes. We then went to the intersection of the above correlated genes and put them into an algorithm of consensus clustering (“ConsensusClusterPlus” R package) to classify the LUADs in the training cohort. We selected the optimal value of k for forming our CoCu clusters by evaluating the cumulative distribution function (CDF) plot, intragroup consistency plot, and Kaplan-Meier (KM) curve. KM curve was made possible by using the R packages “survival” and “survminer”. Additionally, we employed several R packages, including “GSEABase”, “reshape2”, “limma”, “ggpubr”, and “GSVA”, to execute the single-sample gene set enrichment analysis (ssGSEA) and generate visualizations. We utilized the “limma” R package with an FDR threshold of less than 0.05 to identify the differentially expressed genes identified among CoCu clusters (CoCu-DEGs) among the CoCu clusters. Kyoto Encyclopedia of Genes and Genomes (KEGG) analysis was then conducted on CoCu-DEGs for discovering potential pathways. To perform KEGG analysis, we employed the KEGG API (https://www.kegg.jp/kegg/rest/keggapi.html) to retrieve the most up to date KEGG Pathway gene annotations as a reference for gene mapping. The R package “clusterProfiler” (version 3.14.3) was utilized for conducting enrichment analysis on specific operations. The minimum gene set was set to 5, while the maximum gene set was set to 5000. Results were deemed statistically significant when the *P* value was less than 0.05 and the *FDR* was less than 0.25.

### Development of CoCu-DEG cluster and copper homeostasis and cuproptosis regulated lncRNA signature (CoCuLncSig)

We categorized patients in the training cohort based on CoCu-DEGs and generated KM curves to evaluate survival disparities across the CoCu-DEG clusters. To assess the level of differentiation among the different clusters, we utilized principal component analysis (PCA) by using “scatterplot3d” package in R. Then we conducted the ssGSEA and generated visualizations. Next, we employed the “limma”, “GSEABase”, “GSVA”, and “pheatmap” R packages to perform GSVA to identify the top significant KEGG pathways among the CoCu-DEG clusters. We observed the distribution of 21 copper homeostasis/cuproptosis-regulated genes (STEAP1, SLC31A1, CCS, SOD1, ATOX1, ATP7A, ATP7B, COX17, COX11, SCO1, MT-CO1, FDX1, LIAS, PDHA1, PDHB, MTF1, LIPT1, DLD, DLAT, GLS, and CDKN2A) across CoCu-DEG clusters using boxplot. We explored the lncRNA that were differentially expressed between the CoCu-DEG clusters (DELs) with an FDR threshold of less than 0.05. Subsequently, we conducted univariate Cox and KM analyses on DELs to identify the ones that showed potential prognostic significance with a *p*-value of less than 0.05. The CoCuLncSig was constructed using prognostic DELs and a least absolute shrinkage and selection operator (LASSO) to prevent overfitting. The “glmnet” R package was used to ascertain the model, with the penalty parameter (λ) corresponding to the partial likelihood deviance and tested using tenfold cross-validation. The R package outputted the composition of the CoCuLncSig and the coefficient of each lncRNA. The risk score was calculated by summing the expression level of each lncRNA in the CoCuLncSig multiplied by its corresponding coefficient.

### Validation of the CoCuLncSig in an independent cohort

After assigning a risk score to each LUAD in our study using the formula above, we categorized the population into high and low-risk groups using their medians. In order to evaluate CoCuLncSig’s predictive, accuracy, and discriminative abilities, a variety of bioinformatic analyses were conducted on all populations within the study. These analyses included Cox analysis, receiver operating characteristic (ROC) analysis, time-dependent AUC (tAUC) analysis, and survival nomogram using continuous variables, as well as KM analysis and PCA analysis using categorical variables. The validation process was carried out in R software, utilizing several R packages such as “timeROC”, “survival”, “survminer”, “rms”, “scatterplot3d”, “forestplot”, “limma”, “reshape2”, “ggplot2”, “ggpubr”, and “regplot”. To perform Gene Set Enrichment Analysis (GSEA) [16] for CoCuLncSig, we obtained the “c2.cp.kegg.v7.4.symbols.gmt” [17] subset from http://www.gsea-msigdb.org/gsea/downloads.jsp to assess associated pathways and molecular mechanisms. In our GSEA analysis, we established the minimum gene set as 5, the maximum gene set as 5000, and performed 1000 resamples. We deemed results to be statistically significant if they had a *P*-value < 0.05 and an FDR < 0.25.

### Identification of the immunological status of the CoCuLncSig

The R package “ESTIMATE” utilizes the gene expression levels of the training cohort to compute stromal, immune, and ESTIMATE scores for individual patients [18]. We evaluated the correlation between CoCuLncSig and the above category scores using statistical analysis methods like the Pearson coefficient and the Wilcoxon rank sum test. With R package “IOBR,” immuno-oncology exploration can be facilitated, tumor-immune interactions can be explored, and precision immunotherapy can be expedited [19]. The R package “IOBR” or its algorithms included, namely CIBERSORT [20], CIBERSORT-ABS [20], quanTIseq [21], TIMER [22], MCPCounter [23], xCell [24], EPIC [25], and IPS [26] were applied to assess immune-infiltrating levels of every LUAD in the TCGA-LUAD. To assess the relationship between CoCuLncSig and immune-infiltrating levels, we employed the Pearson coefficient and the Wilcoxon rank sum test, and the outcomes were presented as lollipop plots and heatmaps. We summarized the aforementioned findings through Venn and cloud diagrams, and assessed the immune function of CoCuLncSig utilizing the ‘ssGSEA’ function available in the ‘gsva’ R package.

### Identification of CoCuLncSig’s role in immunotherapy and its potential checkpoint targets

Mariathasan’s study [27] signature provided us with a set of genes associated with the immune checkpoint blocker (ICB) response, while Xu et al.’s web portal [28] gave us gene sets linked to the steps of the tumor immune cycle. As the immune microenvironment influences both ICB responses and immune cycle steps, we aimed to leverage this information to investigate the potential role of CoCuLncSig in LUAD immunity. Specifically, we conducted analyses to examine the correlations between CoCuLncSig and ICB responses, as well as CoCuLncSig and tumor immune cycle steps. We utilized the R language package “maftools” to generate a visual representation of the mutational landscape of genes within the training cohort. To evaluate the correlation between the risk score and the tumor mutational burden (TMB) [29], a commonly used indicator of immunotherapy sensitivity that measures the frequency of specific mutations in tumor genes, we employed a combination of Pearson’s coefficient and the Wilcoxon rank sum test in our study. By utilizing markers of T cell dysfunction and data on T cell exclusion, the Tumor Immune Dysfunction and Exclusion (TIDE) framework models how tumors evade detection by the immune system [30–32]. We also determined the correlation between our signature and the TIDE using Pearson’s coefficient and Wilcoxon rank sum. To assess immunotherapy capacity in the CoCuLncSig, we obtained TCGA-LUAD immunotherapy response data from the TIDE portal and done visualization using a ridgeline plot and percent stacked column chart. In our study, we chose a set of 60 immune checkpoints that had been previously investigated, which included 24 inhibitory and 36 stimulatory checkpoints [33] (Supplementary Table 1). To evaluate the relationships between our CoCuLncSig and the 60 selected immune checkpoints, we conducted integration analysis including Pearson coefficient and Wilcoxon rank-sum analyses. We sought to determine if our CoCuLncSig could serve as a guide for immunotherapy. To this end, we utilized the KM and Cox analysis to assess the outcome predictive value of 60 immune checkpoints. Using a Venn diagram, we summarized the results to identify potential checkpoints with targeting ability related to that of the CoCuLncSig. We gathered immunotherapy-related data from various published immune datasets and analyzed the effects of identified checkpoints on immunotherapy outcomes. This particular step was carried out through the “regulator prioritization” module in the TIDE online tool [31].

### Drug selection for patients with high CoCuLncSig score LUAD

Data regarding drug susceptibility in cancer cell lines (CCLs) was downloaded from two sources, namely the Cancer Therapeutics Response Portal (CTRP) at https://portals.broadinstitute.org/ctrp and PRISM Repurpose at https://depmap.org/portal/prism/. The CTRP evaluated 481 compounds across 835 CCLs, while PRISM Repurpose assessed 1448 compounds across 482 CCLs. In both datasets, the drug sensitivity was determined by the area under the dose-response curve (AUC), with lower values indicating greater sensitivity. Our study involves the analysis of drug response data from CTRP and PRISM to identify feasible drug candidates from the high-scoring group. To do this, we compared drug responses between patients with the highest and lowest decile risk scores and used a threshold of log_2_FC > 0.05 to screen for drugs with lower AUC in high-scoring patients [34]. To select compounds with a negative correlation between AUC values and risk scores, we performed Spearman correlation analysis. We set our screening threshold at a Spearman correlation coefficient [34] of less than −0.3.

### Validation of drug candidates

Additional validation analyses were conducted on the results of the drug candidate, which involved reviewing the data of clinical trial and published experimental evidence, and the use of Connectivity Map (CMap) to further confirm its potential in LUAD [34]. CMap is a tool that generates and examines large perturbed datasets, aiding in the comprehension of human disease and speeding up the identification of new treatments. CMap’s datasets, processing, and analysis capabilities are utilized to advance drug research [34]. In this study, we employed CMap analysis as a supplementary approach to explore the potential efficacy of the identified drug candidates in LUAD. A total of 2429 compounds were available for analysis on the CMap online analysis portal (https://clue.io/query). We conducted a differential expression analysis to compare tumor and normal samples. We then selected the top 150 up-regulated and the top 150 down-regulated genes based on the fold difference results and submitted them for analysis on the CMap online analysis portal. Each compound’s CMap result is represented as a value between −100 and 100, with a result closer to -100 indicating a greater potential for therapeutic power.

### Comparing CoCuLncSig with previous studies

In order to conclude whether our study is more robust than previous, we searched PubMed using keywords “copper lncRNA signature lung adenocarcinoma” or “cuproptosis lncRNA signature lung adenocarcinoma” to find candidate study. We included the research that contained a lncRNA signature and the related coefficient. Because most of the candidate studies did not upload raw data or used different or unmentioned data preprocessing methods, therefore, to ensure the standard consistency of the comparison, we use the official TCGA data for analysis here, which are TCGA-LUAD_PanCanAtlas from Genomic Data Commons, Pan-Cancer Atlas (https://gdc.cancer.gov/about-data/publications/pancanatlas), and TCGA-LUAD_Count, TCGA-LUAD_FPKM_UQ, and TCGA-LUAD_FPKM from Genomic Data Commons Data Portal (https://portal.gdc.cancer.gov/). For specific comparative analysis, we used Cox regression analysis.

### CoCuLncSig’s expression pattern determination by qRT-PCR and its pan-cancer ability assessment

To investigate the expression status of CoCuLncSig lncRNAs in real-life scenarios, we conducted qRT-PCR on clinical obtained human tissue samples from our facility. We collected nine LUAD tissues and their corresponding adjacent normal tissues from nine clinical patients. This approach was approved by the Ethics Review Committee of the First Affiliated Hospital of Zhengzhou University, and informed consent was obtained from all patients prior to surgery. None of the patients had received any kind of therapy before undergoing the surgical operation. Tissue samples were immediately frozen and stored in liquid nitrogen after extraction during the surgery. The TRIzol reagent (Invitrogen, Thermo Fisher Scientific corporation, USA) was used to extract total RNA from sample tissues, following the manufacturer’s instructions. The extracted RNA (1 μg) was reverse transcribed using the PrimeScript RT reagent kit (TAKARA BIO INC., Shiga, Japan). The resulting cDNA was used in triplicate for qRT-PCR, performed with SYBR^®^ Premix Ex Taq™ (Perfect Real-Time) (TAKARA BIO INC., Shiga, Japan). The qRT-PCR conditions involved 40 cycles of 95°C for 30 s, 95°C for 10 s, and 60°C for 30 s. The internal reference used was GAPDH and the primer sequences are listed in Table 1. Gene expression was quantified using the 2^−ΔΔCt^ method. The statistical analyses were conducted using GraphPad Prism 9.0 software (GraphPad Software, Inc., La Jolla, CA, USA). The data were presented as mean ± standard deviation, and the unpaired Student’s *t*-test was utilized to compare two groups. Statistical significance was indicated by *p* values < 0.05.

**Table 1:** Prognostic LncRNAs obtained from LASSO Cox regression model and their primer sequences.

**Gene Symbol**	**Coefficient**	**Sequence (5′–3′)**	
		**Forward**	**Reverse**
AL691432.2	−0.479622606	AGGCTCTCCAGGACAAGTGA	GGCTCTCTCCAACAACAAGC
AC093010.2	−0.36577691	GGTGAGCCTGAGAGTTGAGG	AGCAGAGGGTGAAGGAGACA
AC107464.3	−0.212021473	GGCAAGAGAATGCTGGTCTC	TCTTTTCTCATGCCCCTCTG
AC025278.1	−0.109850151	CGTTCACCTCTTTTCCAAGC	TGACCTGGTTGTCAGGATGA
COLCA1	−0.079334536	GACAAGTTTGGCTCCTGCTC	CCTCTGTGGACCATTCCTGT
AC026471.3	−0.008162688	CACTCCACCTCCACAGGAGT	ACTTCAGCTTCGCTGGACAT
LINC01833	0.16143488	ACCTCACACTCCACCCAAAG	ATTATGCCTGTGGGCACTTC
ITGB1-DT	0.278740024	AGTTGCGTCCTGCTTTTGAT	CAATCATCGAATCGACATGC

To evaluate the pan-cancer potential of CoCuLncSig, we obtained the TCGA pan-cancer datasets from Genomic Data Commons, Pan-Cancer Atlas (https://gdc.cancer.gov/about-data/publications/pancanatlas). We applied our CoCuLncSig to pan-cancer visualizing the risk score distribution and determining our signature’s prognosis impacts on cancers using Cox regression. R packages of “ggplot2”, “survival”, “cowplot”, and “ggpubr” were making this demonstration possible. We also assessed whether the lncRNAs in CoCuLncSig are differentially expressed between tumors and normal tissues. R packages “ggplot2”, “clusterProfiler”, “ComplexHeatmap”, and “limma” were adopted for the calculation and visualization. Then we conducted the prognostic ability determination for CoCuLncSig lncRNAs using R packages “survival” and “pheatmap”.

### Novelty and impact statements

This study utilized a novel approach by leveraging the publicly available online repository (with a total sample size of > 1000 cases) to develop CoCuLncSig, an eight-lncRNA signature related to copper homeostasis and cuproptosis that can predict LUAD prognosis. We also confirmed the CoCuLncSig’s power by comparing it with previous studies. The CoCuLncSig lncRNAs’ expression patterns were measured using human tissues and qRT-PCR. We identified the specific targets of CoCuLncSig that played vital roles in immunotherapy and highlighted potential therapeutic agents that may be effective for high-risk score LUADs.

### Availability of data and materials

The study utilized a combination of publicly available databases and original data. The TCGA data used for the model training was downloaded from Xena Hub online portal (https://xenabrowser.net/), and GEO data for model validation, GSE29013, GSE30219, GSE31210, GSE37745, and GSE50081, were obtained from https://www.ncbi.nlm.nih.gov/geo. Additionally, data for drug prediction was sourced from the CTRP database, which can be accessed at https://portals.broadinstitute.org/ctrp, as well as the PRISM database, which was downloaded from https://depmap.org/portal/prism. For comparison and pan-cancer assessments, we obtained official TCGA data Genomic Data Commons, Pan-Cancer Atlas (https://gdc.cancer.gov/about-data/publications/pancanatlas) and Genomic Data Commons Data Portal (https://portal.gdc.cancer.gov/). The raw data generated from the qRT-PCR used in the study is available upon request from the corresponding author.

## RESULTS

### Characteristics of LUADs included in the study

**Figure 1:**
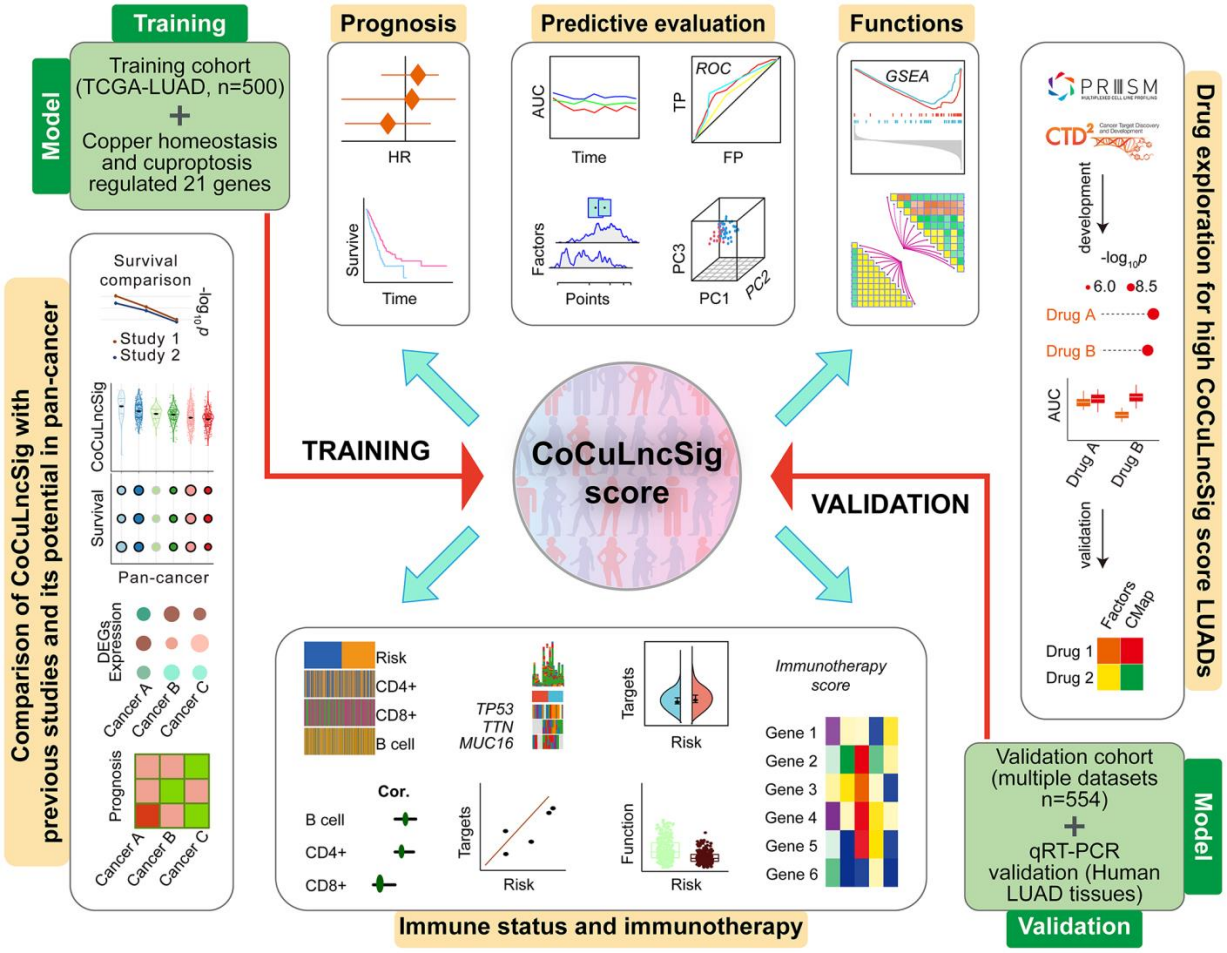
**Flowchart of the main steps, design, and analysis process of this study [80].** Abbreviations: TCGA: The Cancer Genome Atlas; LUAD: lung adenocarcinoma; CoCuLncSig: copper homeostasis and cuproptosis regulated lncRNA signature; HR: hazard ratio; ROC: receiver operating characteristic; AUC: area under the ROC curve; TP: true positive rate; FP: false positive rate; PC: principal component; GSEA: gene set enrichment analysis; CMap: Connectivity Map; DEGs: differentially expressed genes; qRT-PCR: quantitative real-time PCR.

**Figure 2:**
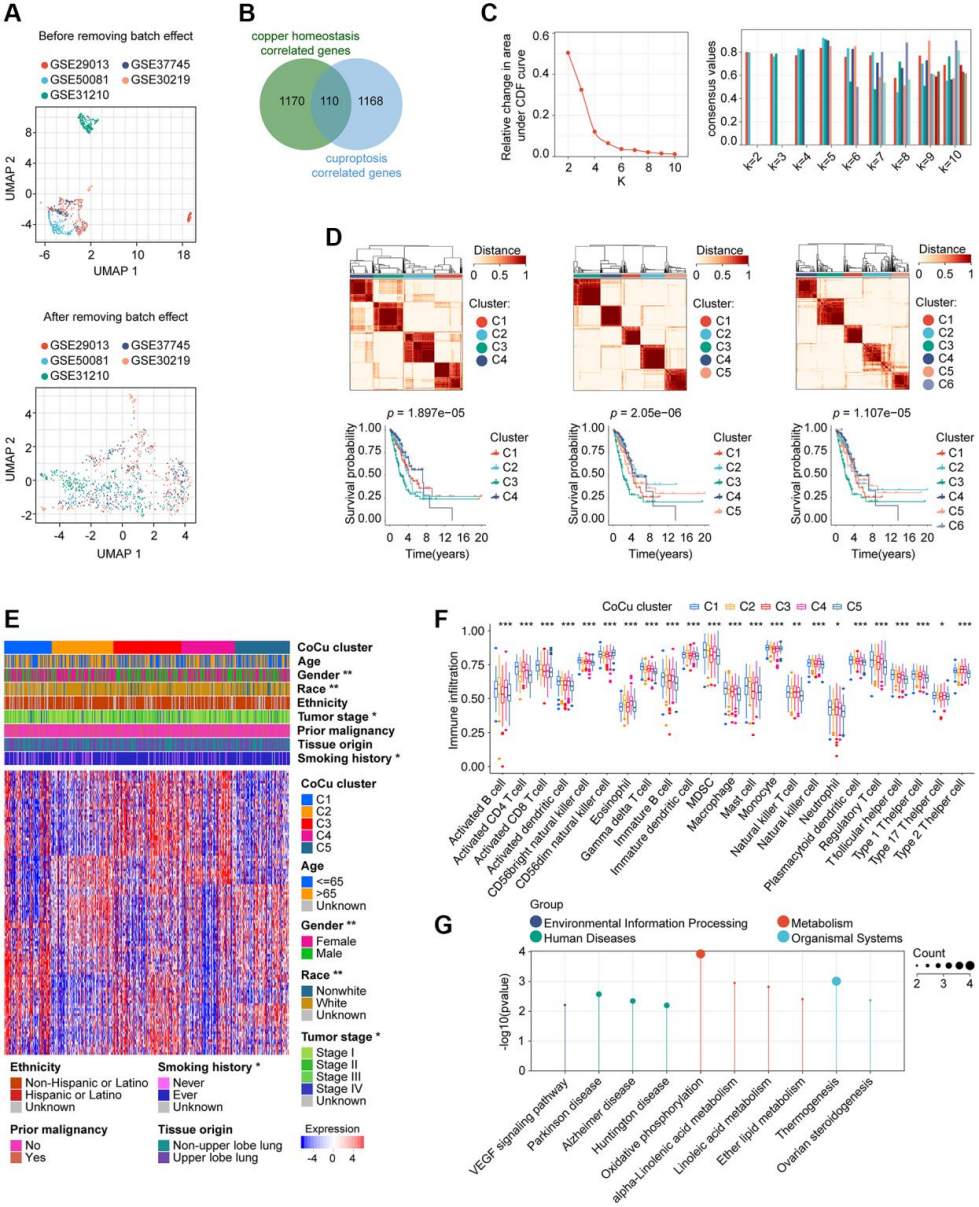
**Removal of batch effects for the validation cohort and construction of the CoCu cluster**. (**A**) The validation cohort is visually compared before and after the elimination of batch effects. The UMAP plot displayed at the top depicts the combined state of the GEO dataset before removing the batch effect. Conversely, the UMAP plot in the lower section illustrates that after removing the batch effect from the merged dataset, the samples are interwoven, providing proof of the effectiveness of batch effect removal. (**B**) 110 genes that correlate to both copper homeostasis and cuproptosis-regulated genes were identified using a Venn diagram. (**C**) CDF plot (left) showing the downward trend of the curve. Consensus plot (right) showing the consensus value at specific k value. (**D**) Based on the evidence provided from CDF and consensus plots, we were more interested in the clustering situation when *k* = 4, 5, and 6. Clustering diagrams (upper) for *k* = 4, 5, and 6. KM curves (lower) for each k value. (**E**) The heatmap depicts the correlation between CoCu clusters, clinical parameters, and 110 genes associated with copper homeostasis and cuproptosis. The asterisks indicate statistical differences between CoCu clusters. In the heatmap, each row corresponds to a specific gene, while each column corresponds to a particular sample. (**F**) The box plots illustrate notable variations in the distribution of all 23 immune cell types across the 5 CoCu clusters, indicating statistical significance. (**G**) KEGG that was performed using 72 CoCu-DEGs showing the top enriched pathways. CoCu clusters: clusters identified by copper homeostasis and cuproptosis correlated genes; CoCu-DEGs: differentially expressed genes identified among CoCu clusters; UMAP: Uniform Manifold Approximation and Projection; CDF: cumulative distribution function; KM: Kaplan–Meier estimator; DEGs: differentially expressed genes; KEGG: Kyoto Encyclopedia of Genes and Genomes; A statistically significant *P*-value was defined as being less than 0.05; The following notation was used: ^*^ for *P*-values less than 0.05, ^**^ for *P*-values less than 0.01, and ^***^ for *P*-values less than 0.001.

The general flowchart of our study is illustrated in Figure 1. To construct the training cohort, we included 500 LUAD patients from TCGA-LUAD, while the validation cohort comprised of 554 LUAD patients from five GEO datasets (GSE29013, GSE30219, GSE31210, GSE37745, and GSE50081), selected based on our predefined criteria. Prior to the analysis, we merged the five GEO datasets and removed any batch effects, as demonstrated in Figure 2A. The UMAP plot revealed that the samples from each dataset were distinct prior to the removal of batch effects. However, after utilizing Johnson et al.’s batch effect removal method [15], the datasets displayed interleaving, which suggests that the technique was effective in removing the batch effect. Table 2 presents the clinical data of the patients included in each cohort of this study.

**Table 2:** The clinical baseline conditions of the cohorts and patients included in the study.

**Characteristics**	**Training cohort**	**Validation cohort**
	**(TCGA-LUAD, *n* = 500)**	**(GSE29013, GSE30219, GSE31210, GSE37745, and GSE50081, *n* = 554)**
Age		
<65	219 (43.8%)	315 (56.86%)
≥65	271 (54.2%)	239 (43.14%)
Unknown	10 (2%)	0
Gender		
Female	270 (54%)	265 (47.83%)
Male	230 (46%)	289 (52.17%)
Race		
White	386 (77.2%)	NA
Non-White	60 (12%)	NA
Unknown	54 (10.8%)	NA
Ethnicity		
Hispanic or Latino	7 (1.4%)	NA
Non-Hispanic or Latino	381 (76.2%)	NA
Unknown	112 (22.4%)	NA
Tumor stage		
Stage I	268 (53.6%)	339 (61.19%)
Stage II	119 (23.8%)	108 (19.49%)
Stage III	80 (16%)	21 (3.79%)
Stage IV	25 (5%)	4 (0.72%)
Unknown	8 (1.6%)	82 (14.8%)
Prior malignancy		
Yes	79 (15.8%)	NA
No	421 (84.2%)	NA
Tissue origin		
Upper lobe lung	291 (58.2%)	NA
Non-upper lobe lung	209 (41.8%)	NA
Smoking history		
Ever	415 (83%)	216 (38.99%)
Never	71 (14.2%)	139 (25.09%)
Unknown	14 (2.8%)	199 (35.92%)
Vital status		
Alive	318 (63.6%)	348 (62.82%)
Dead	182 (36.4%)	206 (37.18%)

### Construction of CoCu clusters in LUADs using consensus clustering

1280 copper homeostasis-correlated genes and 1278 cuproptosis-correlated genes were obtained, and a total of 110 genes were in their intersection (Figure 2B). An algorithm of consensus clustering classified training cohort LUADs into 2, 3, 4, 5, 6, 7, 8, 9, and 10 clusters, respectively, based on the 110 copper homeostasis/cuproptosis correlated genes. We observed the CDF plot for the downward trend of the curve, finding that when *k* = 4, 5, or 6 may potentially arouse our interest (Figure 2C). In addition, we examined the intragroup consistency of each group by checking the consensus values, and the results indicated that when k is 5, the clusters have the highest average consistency. When k is 4, the consistency of the cluster ranked the second highest (Figure 2C). Based on the evidence, we are more interested in the clustering situation when *k* = 4, 5, and 6. In Figure 2D upper parts, we plotted the clustering diagrams for *k* = 4, 5, and 6. It could be seen that when *k* = 5, the clustering was relatively neat and well gathered. Next, we constructed the KM curve for each *k* value (Figure 2D, lower parts), and found that the *p* value was smaller than the others’ when *k* = 5, which indicated that the survival difference was the most pronounced. We then take the cluster with *k* = 5 as our copper homeostasis/cuproptosis related (CoCu) cluster (Figure 2E). Furthermore, there were statistically significant differences in five CoCu clusters of LUAD patients in terms of clinical factors like gender, race, tumor stage, and smoking history (Figure 2E). The ssGSEA method was used to determine the infiltration levels of various immune cell populations in the five CoCu clusters. Figure 2F illustrates that the distribution of all 23 types of immune cells across the clusters differed significantly from a statistical perspective. By comparing the five CoCu clusters, we carried out an analysis to find differentially expressed genes and identified 72 CoCu-DEGs (Supplementary Table 2). The KEGG was performed using these 72 CoCu-DEGs and demonstrated the top ten pathways related to the CoCu cluster, which were VEGF signaling pathway, Parkinson disease, Alzheimer disease, Huntington disease, oxidative phosphorylation, alpha-Linolenic acid metabolism, linoleic acid metabolism, ether lipid metabolism, thermogenesis, and ovarian steroidogenesis (Figure 2G).

### Two CoCu-DEG clusters determined and a CoCuLncSig generated

**Figure 3:**
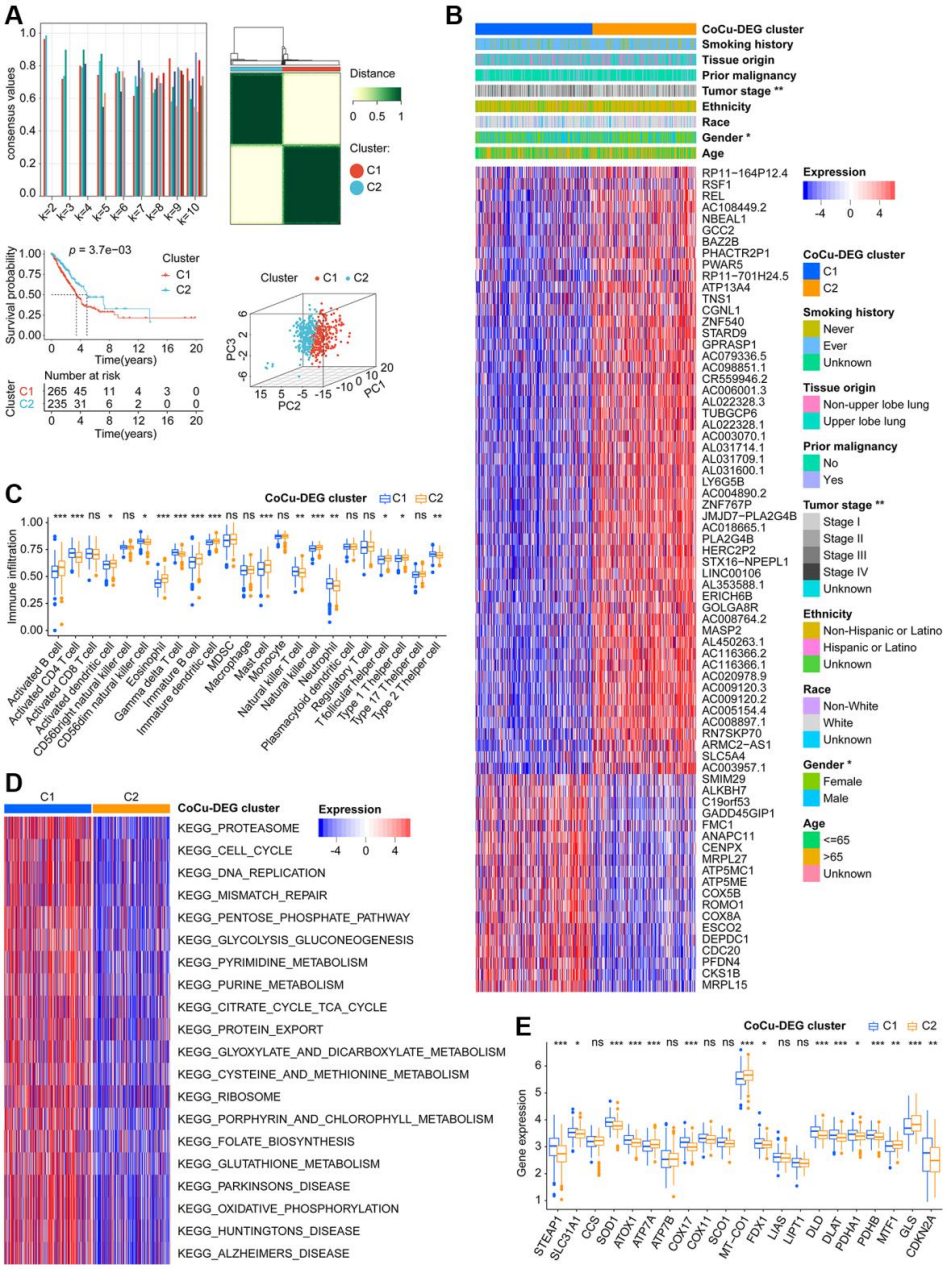
**Two CoCu-DEG clusters identified**. (**A**) The development process of CoCu-DEG clusters, and its KM and PCA performance. 72 CoCu-DEGs were applied for the clusters’ generation. Consensus plot (top left) showing the consensus value at specific k value. Clustering diagrams for *k* = 2 displaying at the top right. KM curves for *k* = 2 is at the lower left and PCA plot is at the lower right for *k* = 2. (**B**) The heatmap depicts the correlation between CoCu-DEG clusters, clinical parameters, and 72 CoCu-DEGs. The asterisks indicate statistical differences between CoCu clusters. In the heatmap, each row corresponds to a specific gene, while each column corresponds to a particular sample. (**C**) The box plots illustrate variations in the distribution of 23 immune cell types across the two CoCu-DEG clusters. The asterisks indicate distribution statistical differences between clusters. (**D**) A heatmap was used to visualize the enrichment of KEGG pathways that were present between the CoCu-DEG clusters, which was carried out utilizing the R package “GSVA.” (**E**) Box plots show the distribution of 21 copper homeostasis/cuproptosis-regulated genes across CoCu-DEG clusters. CoCu clusters: clusters identified by copper homeostasis and cuproptosis correlated genes; CoCu-DEGs: differentially expressed genes identified among CoCu clusters; CoCu-DEG clusters: clusters identified by CoCu-DEGs; DEGs: differentially expressed genes; KM: Kaplan–Meier estimator; PCA: Principal component analysis; KEGG: Kyoto Encyclopedia of Genes and Genomes; In Figure 3, statistical significance was defined as a *P*-value < 0.05; Results with *P*-values greater than or equal to 0.05 were considered not significant (ns), while those with *P*-values less than 0.05, 0.01, and 0.001 were denoted by ^*^, ^**^, and ^***^, respectively.

Using CoCu-DEG as a basis, we attempted to classify the training cohort LUADs into 2, 3, 4, 5, 6, 7, 8, 9, and 10 clusters, respectively (Figure 3A, upper-left). We examined the intragroup consistency of each group by checking the consensus values, and the results indicated that when k is 2, the clusters have the highest average consistency (Figure 3A, upper-left). We plotted the clustering diagram for *k* = 2, showing the clustering was relatively neat and well gathered (Figure 3A, upper-right). Next, we constructed the KM curve for *k* = 2 cluster, finding that the survival difference in clusters was statistically significant (Figure 3A, lower-left). Based on the results of PCA, it was observed that clusters C1 and C2 were distinctly separated from each other (Figure 3A, bottom right). Considering these pieces of evidence, we propose that the clusters at *k* = 2, namely the CoCu-DEG clusters (Figure 3B), can be the focus of our study. Notably, the analysis revealed that LUAD patients in the CoCu-DEG cluster exhibited significant differences in terms of gender and tumor stage (Figure 3B). Further, by utilizing ssGSEA, we were able to determine the extent of infiltration of various types of immune cells within the CoCu-DEG clusters. Figure 3C depicted that among the CoCu-DEG clusters, 15 distinct immune cells, namely Activated B cell, Activated CD4 T cell, Activated dendritic cell, CD56dim natural killer cell, Eosinophil, Gamma delta T cell, Immature B cell, Immature dendritic cell, Mast cell, Natural killer T cell, Natural killer cell, Neutrophil, T follicular helper cell, Type 1 T helper cell, and Type 2 T helper cell, were significantly distributed. To identify the key KEGG pathways between the CoCu-DEG clusters, we conducted GSVA (Figure 3D, Supplementary Table 3). Surprisingly, KEGG_PROTEASOME, KEGG_DNA_REPLICATION, KEGG_PARKINSONS_DISEASE, KEGG_OXIDATIVE_PHOSPHORYLATION, KEGG_CELL_CYCLE, KEGG_PENTOSE_PHOSPHATE_PATHWAY, KEGG_HUNTINGTONS_DISEASE, KEGG_PYRIMIDINE_METABOLISM, KEGG_ALZHEIMERS_DISEASE, and KEGG_PORPHYRIN_AND_CHLOROPHYLL_METABOLISM ranked as the top 10 pathways. Notably, we looked at the distribution of 21 copper homeostasis/cuproptosis-regulated genes across CoCu-DEG clusters and found 15 genes (STEAP1, SLC31A1, SOD1, ATOX1, ATP7A, COX17, MT-CO1, FDX1, DLD, DLAT, PDHA1, PDHB, MTF1, GLS, and CDKN2A) were related to the clusters (Figure 3E).

**Figure 4:**
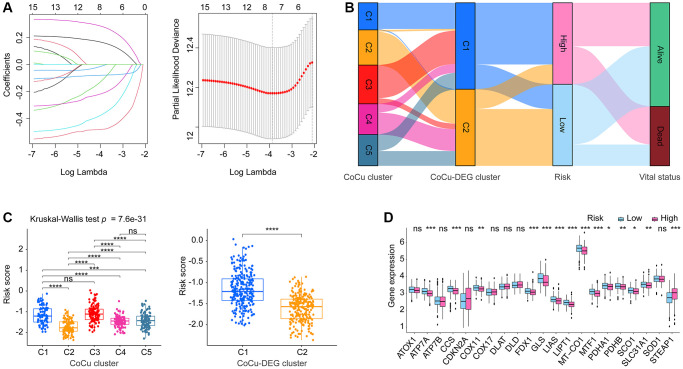
**The establishment of the risk model CoCuLncSig and its basic performance demonstration.** (**A**) This visualization depicts the reduction of dimensionality for prognostic lncRNAs through the use of the LASSO algorithm. The left section of the visualization displays the LASSO coefficient profile for the examined prognostic lncRNAs, while the right section illustrates the LASSO regression process employing ten-fold cross-validation and minimal Lambda to identify eight prognostic lncRNAs. (**B**) The relationship between CoCu clusters, CoCu-DEG clusters, risks, and vital status in general is illustrated by the Sankey diagram. The diagram reveals that a notable portion of the C1 clusters in CoCu-DEG display high-risk scores, while the majority of its C2 clusters exhibit low-risk scores. (**C**) The box plots on the left panel demonstrate distinct statistical variations in the distributions of risk scores across the five CoCu clusters. On the right panel, the box plots exhibit statistically different distributions of risk scores in the two CoCu-DEG clusters. (**D**) Box plots display expression pattern of the 21 copper homeostasis/cuproptosis-regulated genes in the high- and low-risk groups. CoCuLncSig: copper homeostasis and cuproptosis regulated lncRNA signature; CoCu clusters: clusters identified by copper homeostasis and cuproptosis correlated genes; CoCu-DEGs: differentially expressed genes identified among CoCu clusters; CoCu-DEG clusters: clusters identified by CoCu-DEGs; DEGs: differentially expressed genes; LASSO: least absolute shrinkage and selection operator; A *P*-value less than 0.05 was considered significant for statistical analysis; The notation ^*^ represents *P*-value less than 0.05, ^**^ represents *P*-value less than 0.01, ^***^ represents *P*-value less than 0.001, and ^****^ represents *P*-value less than 0.0001.

6646 DELs were identified based on our predefined criteria. Subsequent KM and Cox analyses were performed to screen these DELs, and only 15 of them met our criteria (Supplementary Table 4). Further narrowing down of the results was done using LASSO analysis on these 15 DELs, which identified eight lncRNAs and their corresponding coefficients (Figure 4A, Table 1). To explore the relationships among CoCu clusters, CoCu-DEG clusters, risks, and vital status, a Sankey diagram was created (Figure 4B). We also observed significant differences in risk scores among the CoCu clusters (Figure 4C). Interestingly, the risk scores among the CoCu-DEG clusters were also significantly different. We next tested the expression situation of the 21 copper homeostasis/cuproptosis-regulated genes in the high- and low-risk groups finding 14 genes (STEAP1, SLC31A1, CCS, ATP7A, COX11, SCO1, MT-CO1, FDX1, LIAS, LIPT1, PDHA1, PDHB, MTF1, and GLS) significantly differently expressed (Figure 4D). Among the 14 genes found, only STEAP1 was up-regulated, while the remaining genes were down-regulated in the high-risk group. In addition, we have shown the association between the 21 copper homeostasis/cuproptosis-regulated genes and each CoCuLncSig lncRNA, which is illustrated in Supplementary Figure 1A.

### Validation results demonstrated robust prognostic ability of CoCuLncSig

**Figure 5:**
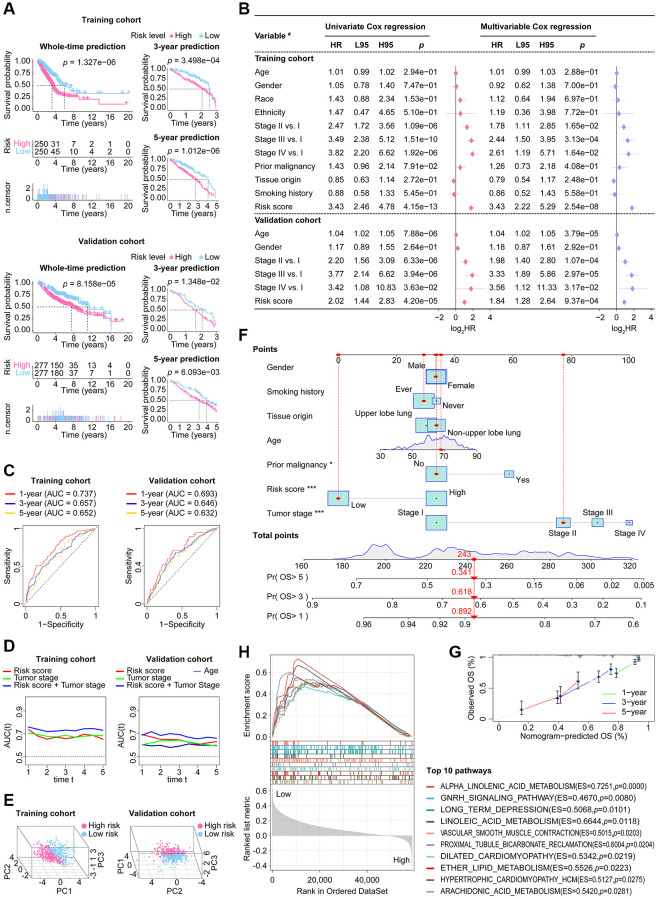
**The stability and applicability of CoCuLncSig were validated in the study cohorts.** (**A**) The prognostic value of CoCuLncSig was demonstrated through Kaplan-Meier analysis in both the training and validation cohorts, which also affirms its broad applicability. By using their median CoCuLncSig risk scores, patients were stratified into high- and low-risk groups, and Kaplan-Meier analysis revealed significant differences in survival between the two groups. (**B**) Univariate and multivariate Cox proportional hazards models were built, incorporating risk scores and several clinical variables. ^#^: the types of variables involved in the studied cohorts. The types of variables included in the analysis were defined as follows: Gender (male vs. female), Race (white vs. non-white), Ethnicity (Hispanic or Latino vs. non-Hispanic or Latino), Prior malignancy (yes vs. no), Tumor origin (upper lobe lung vs. non-upper lobe lung), and Smoking history (ever vs. never). (**C**) ROC curves. Our signature’s accuracy in predicting LUAD outcomes at 1-year, 3-year, and 5-year intervals was evaluated using ROC curves. (**D**) The purpose of the tAUC analysis was to continually assess the prognostic precision of our signature relative to other clinical measures over successive time intervals. An increase in the AUC size is indicative of a more robust predictive accuracy of the model. (**E**) The principal component analysis visualization clearly indicates that the signature is capable of distinguishing the LUAD population. (**F**) A nomogram model was created that predicts the clinical outcome of LUAD patients using seven factors: risk score, tumor stage, age, grade, smoking history, prior malignancy, and tissue origin. This model forecasts the overall survival of patients for 1, 3, and 5 years in the TCGA-LUAD cohort. The significance of the results was indicated using asterisks, where ^*^ represents a *p*-value of < 0.05 and ^***^ represents a *p*-value of < 0.001. (**G**) 1-, 3-, and 5-year overall survival calibration plots for LUAD patients based on the predictive nomogram model. These plots depict the predicted survival rate on the X-axis and the actual survival rate of LUAD patients on the Y-axis. The 45° line on the graph indicates the optimal predicted value. A curve that closely follows the 45° line indicates better results. (**H**) The GSEA analysis identified 10 KEGG pathways with the strongest association with CoCuLncSig. These pathways’ significance thresholds were established as *p*-value < 0.05 and FDR < 0.25. CoCuLncSig: copper homeostasis and cuproptosis regulated lncRNA signature; L95: 95% confidence interval lower; H95: 95% confidence interval higher; HR: hazard ratio; AUC: area under the ROC curve; ROC: receiver operating characteristic; tAUC: time-dependent AUC; TCGA: The Cancer Genome Atlas; GSEA: Gene Set Enrichment Analysis; LUAD: lung adenocarcinoma; OS: overall survival; KEGG: Kyoto Encyclopedia of Genes and Genomes; FDR: false discovery rate; A statistical significance was deemed to be present when the *P*-value was less than 0.05.

The fundamental performance of our CoCuLncSig in both the training and validation cohorts is demonstrated in Supplementary Figure 1B, 1C, respectively. In our analysis of KM, we made survival predictions for whole-time, 3-year, and 5-year periods. The results indicated that both the high-risk LUADs in the training cohort (Figure 5A, upper) and those in the validation cohort (Figure 5A, lower) had a worse prognosis than those in the low-risk groups. Additionally, the KM curve presented in Supplementary Figure 2A of the supplementary material illustrates the prognostic ability of each CoCuLncSig lncRNA in the two cohorts. This curve reveals that ITGB1-DT and LINC01833 were associated with a worse prognosis for LUADs, while AL691432.2, AC093010.2, AC107464.3, AC025278.1, COLCA1, and AC026471.3 were associated with improved outcomes in LUADs. Our analysis then determined whether the risk score was a reliable predictor of outcomes for LUAD patients, independent of clinical parameters such as age, gender, race, ethnicity, tumor stage, tumor origin, etc. To this end, univariate and multivariable analyses were performed, as illustrated in Figure 5B. The risk scores displayed significant prognostic ability (*p* ≤ 4.20e-05) in univariate Cox regression analysis for both the training and validation cohorts. In the training cohort, the risk score had a hazard ratio of 3.43 (95% CI: 2.22–5.29, *p* = 2.54e-08) in multivariate Cox analysis, while in the validation cohort, the risk score had a hazard ratio of 1.84 (95% CI: 1.28-2.64, *p* = 9.37e-04). These results indicate that the risk score performed well in both cohorts and can be considered an independent prognostic factor. Interestingly, while the ‘age’ factor showed independent prognostic power in the validation cohort, it did not have any independent predictive power in the training cohort. In Supplementary Figure 2B of the supplementary material, we have provided an analysis of the results and visualization for each CoCuLncSig lncRNA using univariate Cox regression. ROC analysis (Figure 5C) and tAUC (Figure 5D) were utilized to evaluate the accuracy of the model. The CoCuLncSig AUC in the training cohort was determined to be 0.737, 0.657, and 0.652 at one year, three years, and five years, respectively, as indicated by the ROC curves. In the validation cohort, the AUC was found to be 0.693, 0.646, and 0.632 at one year, three years, and five years, respectively. The CoCuLncSig model’s accuracy was continuously assessed using tAUC. In the training cohort (Figure 5D, left), our risk score was found to be in close proximity to the tumor stage, as determined by tAUC. This was also observed in the validation cohort (Figure 5D, right), where the model’s tAUC was also comparable to the tumor stage, which has been regarded as the gold standard for prognosis prediction. Remarkably, when we combined our risk score with the tumor stage for tAUC, the predictive AUC of the combination was consistently above 0.7 in the training cohort (Figure 5D, left) and outperformed other factors at all time points in the validation cohort (Figure 5D, right). These findings indicate that our CoCuLncSig risk score’s accuracy is comparable to that of the tumor stage and an excellent complement to it. Figure 5E of the study showed that there was significant heterogeneity between high-risk and low-risk patients in the study cohorts based on the results of PCA. This suggests that the risk score model is effective in distinguishing these two groups. Furthermore, a nomogram (Figure 5F) was developed using clinical parameters such as age, tumor stage, gender, smoking history, tissue origin, prior malignancy, and risk score. This nomogram has the potential to assist in determining the 1-year, 3-year, and 5-year prognosis status of clinical patients. The predictive accuracy of the nomogram was confirmed by the calibration curve (Figure 5G). The GSEA analysis demonstrated the most vital ten CoCuLncSig related KEGG pathways were related to alpha-Linolenic acid metabolism, gonadotropin-releasing hormone signaling pathway, long-term depression, linoleic acid metabolism, vascular smooth muscle contraction, proximal tubule bicarbonate reclamation, dilated cardiomyopathy, ether lipid metabolism, hypertrophic cardiomyopathy, and arachidonic acid metabolism (Figure 5H).

### CoCuLncSig is linked to LUAD immune status

**Figure 6:**
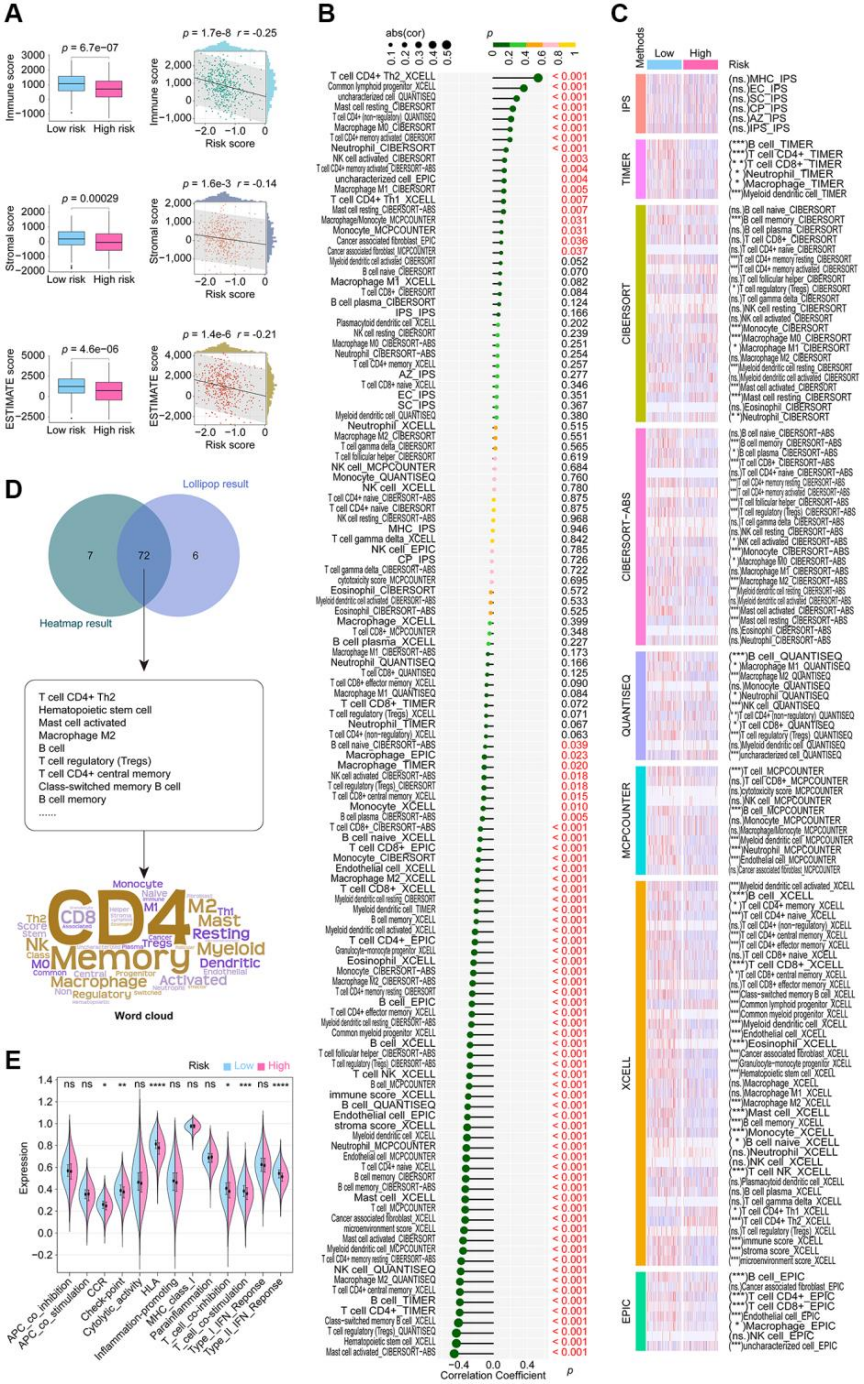
**Extensive examination to investigate the correlation between CoCuLncSig and the tumor microenvironment status, immune cell infiltration, and immune function.** (**A**) Boxplots compared the distribution of immune, stromal, and ESTIMATE scores between high and low-risk groups. The correlation of risk score with immune, stromal, and ESTIMATE scores was depicted using scatterplots. (**B**) Lollipop plots visualize the correlation of immune cell infiltration with CoCuLncSig scores. Here, the R language package “IOBR” generates the immune cell infiltration based on the training cohort data. (**C**) The heatmap demonstrates the immune cell infiltration distributions in high and low CoCuLncSig score population. (**D**) A Venn diagram (upper plot) depicts the intersection between the outcomes of the correlation analysis and the distributional differences. Word clouds (lower plot) were utilized to emphasize crucial immune cell-infiltrating cell types that emerged from this intersection. (**E**) The violin plots display variations in the immune function distribution between the high-risk and low-risk LUADs. CoCuLncSig: copper homeostasis and cuproptosis regulated lncRNA signature; LUAD: lung adenocarcinoma; A *P*-value below 0.05 was deemed as statistically significant. “ns” indicates non-significance, “^*^” represents a *P*-value below 0.05, “^**^” signifies a *P*-value below 0.01, “^***^” denotes a *P*-value below 0.001, and “^****^” indicates a *P*-value below 0.0001.

According to the consensus among the research community, cancer is characterized as a dynamic ecosystem in which malignant and noncancerous cells in the tumor microenvironment collaborate to facilitate the advancement of the disease. As a result, in order to properly examine LUAD, it is imperative to thoroughly investigate its tumor microenvironment. We utilized data from the TCGA cohort and the R language package “ESTIMATE” finding that the high-risk group exhibited decreased stromal, immune, and ESTIMATE scores. Additionally, all scores demonstrated a negative correlation with CoCuLncSig, as illustrated in Figure 6A. By utilizing eight mainstream immune informatics algorithms and employing the Pearson correlation coefficient test and Wilcoxon rank sum test analysis methods, we were able to visually represent the relationship between CoCuLncSig and various immune components through a lollipop (Figure 6B) and heatmap (Figure 6C). To simplify the findings and present crucial information to readers, a Venn diagram (Figure 6D) was used to intersect the results, revealing that CD4 T cells, Memory B cells, Macrophages, Myeloid dendritic cells, and Mast cells are most likely to connect CoCuLncSig and LUAD immune status. Regarding CoCuLncSig’s immune function, the immune functions of the high-score group, such as CCR, Check-point, HLA, T_cell_co-inhibition, T_cell_co-stimulation, and Type_II_IFN_Response, were comparatively weak compared to the low-risk group (Figure 6E). These results suggest that CoCuLncSig may have a connection to the immune status of LUAD.

### CoCuLncSig participates in immunotherapy and targets immune checkpoints

**Figure 7:**
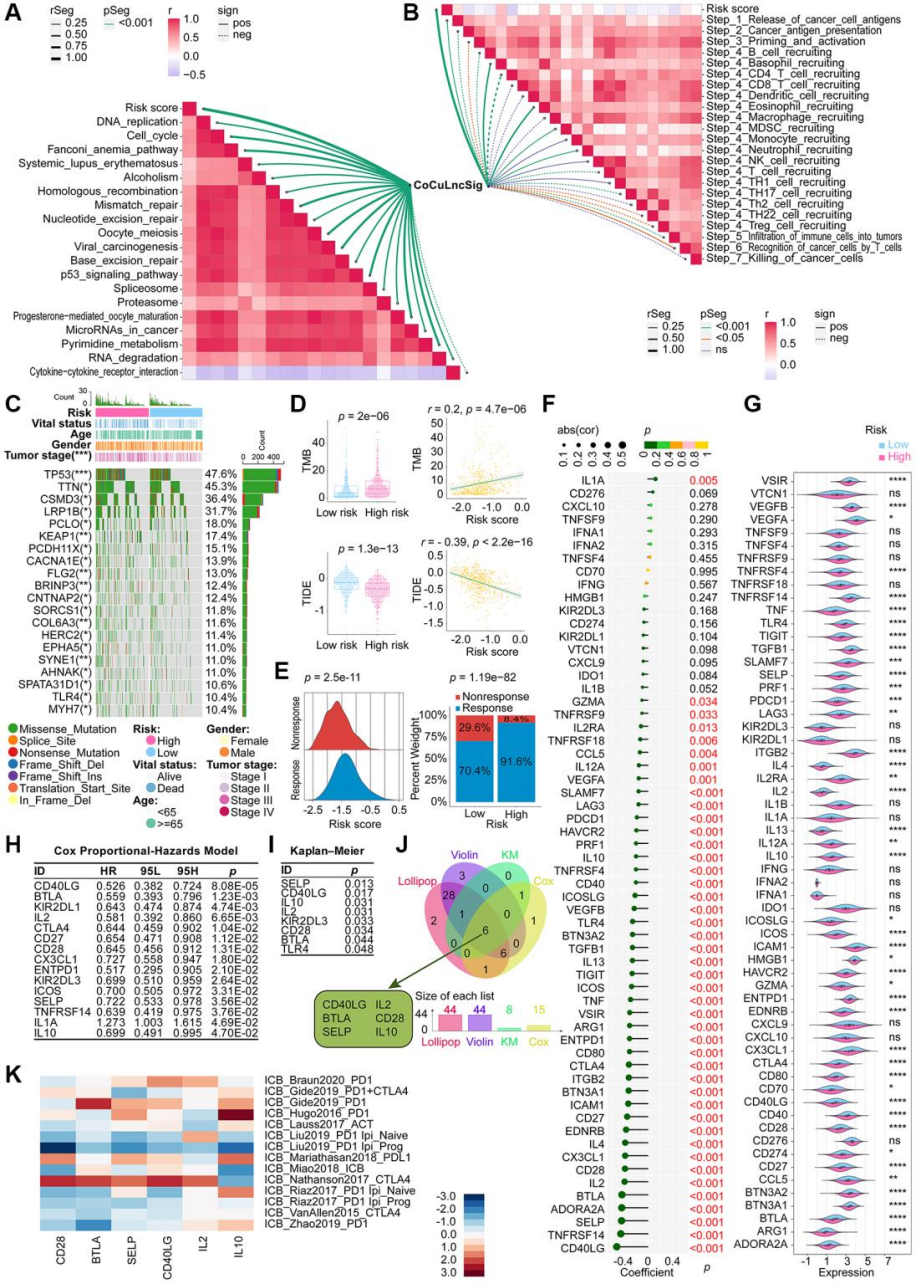
**Demonstration of the relationship between the CoCuLncSig and immunotherapy [80].** Correlation between risk score and ICB response signature (**A**) and correlation between risk score and each step of the tumor immune cycle (**B**). (**C**) A waterfall plot displays the mutational landscape of the 20 most frequently mutated genes in LUAD. Furthermore, the plot showcases the variations in mutations of these genes between the high-risk and low-risk LUADs. (**D**) Boxplots on the left show the difference in the distribution of TMB and TIDE among high-risk and low-risk patients by the Wilcoxon rank sum test. On the right side, scatterplots depict the correlations between risk scores and TMB and TIDE, evaluated by Pearson analysis. (**E**) The ridgeline plot (left) presents the distribution of risk score variation in nonresponse and response LUADs. The proportion (right) of patients with response and nonresponse to immunotherapy in the high and low CoCuLncSig score groups. (**F**) Lollipop plots visualize the correlation between CoCuLncSig and immune checkpoints. Correlation was detected by Pearson’s coefficient test. (**G**) Violin plots showing differences in expression of immune checkpoint genes between high and low risk groups. Differences in expression were analyzed using the Wilcoxon rank sum test. (**H**) Cox analysis was performed to reveal the prognostic potential of the 60 checkpoint genes. The Cox results showed that 15 checkpoint genes had prognostic ability. (**I**) KM analysis evaluated whether high-expression and low-expression checkpoint genes had the predictive ability for LUAD outcomes. The KM results demonstrated that 8 out of 60 checkpoint genes could discriminate LUAD prognosis. (**J**) The Venn diagram merges findings from correlation analysis, difference distribution analysis, Cox analysis, and KM analysis to identify the checkpoint genes associated with CoCuLncSig and impacting the prognosis of LUAD. (**K**) A heatmap has been generated to display published datasets’ relative immunotherapy scores for six checkpoint genes. The checkpoint genes, ranked in order of their immunotherapy score from high to low, are IL10, IL2, CD40LG, SELP, BTLA, and CD28. The immunotherapy scores have been subjected to zero-mean normalization. CoCuLncSig: copper homeostasis and cuproptosis regulated lncRNA signature; KM: Kaplan–Meier estimator; TMB: Tumor mutational burden; ICB response: immune checkpoint blockers response; TIDE: Tumor Immune Dysfunction and Exclusion; ns: not significant; rSeg: *r* segment; pSeg: *p*-value segment; sign: significant; pos: positively; neg: negatively; LUAD: lung adenocarcinoma; Asterisks denote statistical significance levels; in this context, the significance levels for *p*-values are as follows: ^*^p-value < 0.05 ^**^p-value < 0.01 ^***^p-value < 0.001 ^****^p-value < 0.0001; A *p* value < 0.05 was used as the threshold for statistical significance.

The top 10 ICB response pathways that CoCuLncSig correlated with were progesterone mediated oocyte maturation, oocyte meiosis, cell cycle, p53 signaling pathway, viral carcinogenesis, pyrimidine metabolism, mismatch repair, Fanconi anemia pathway, homologous recombination, and spliceosome (Figure 7A, Supplementary Table 5). CoCuLncSig correlated with some of the tumor immune cycle steps, which the top 10 ranked were Step 4 CD4 T cell recruiting, Step 4 Basophil recruiting, Step 4 Eosinophil recruiting, Step 5 Infiltration of immune cells into tumors, Step 2 Cancer antigen presentation, Step 4 TH17 cell recruiting, Step 4 MDSC recruiting, Step 4 Neutrophil recruiting, Step 1 Release of cancer cell antigens, and Step 4 B cell recruiting (Figure 7B, Supplementary Table 6). The relationship between CoCuLncSig and ICB response and the involvement of the tumor immune cycle steps further imply its potential connection to certain immune checkpoint treatments.

In the training cohort, we analyzed the mutation profile of all tumor samples and depicted the top 20 genes with the most significant mutations in Figure 7C. TP53 was identified as the most frequently mutated gene, with a prevalence of around 47.6%, followed by TTN at 45.3% and CSMD3 at 36.4%. Our findings revealed that missense mutations were the most commonly observed variant classification across all mutation types.

According to the Wilcoxon test results, the group with a higher risk score exhibited elevated TMB levels (Figure 7D, upper-left), and a positive correlation was observed between risk score and TMB (Figure 7D, upper-right). Immunotherapy may confer longer-lasting clinical benefits for patients with higher TMB [35, 36]. Based on our analysis, it is plausible that our model’s high-scoring LUADs could benefit from immunotherapy. Furthermore, our findings suggest that patients with high-risk scores had lower TIDE scores, and there was an inverse correlation between TIDE scores and risk scores (Figure 7D, lower panel). Patients with higher TIDE scores are more likely to experience immune evasion [30–32], implying that the high-risk population in our model could benefit more from immunotherapy. This finding aligns with our aforementioned discovery regarding TMB. To testify the immunotherapy trend among CoCuLncSig risk score, we obtained immunotherapy response data of the TCGA-LUAD from the TIDE web portal and displayed them in the form of ridgeline plot and percent stacked column chart, as exhibited in Figure 7E. The ridgeline plot indicated that the response population has a higher risk score distribution than the nonresponse cases. The stacked column chart showed the high-risk population had a higher percentage of responses than that in the low-risk group.

In our study, we conducted a literature review to identify immune checkpoint genes, selecting a total of 60 based on previous research [33]. To analyze their relationship with the risk score, we utilized Pearson correlation coefficient analysis and displayed the results using lollipop plots (Figure 7F). As demonstrated in Figure 7F and Supplementary Table 7, 44 of the 60 genes showed a significant association with the risk score. The top 5 were CD40LG (coefficient = −0.504060342, *p* = 1.41E-33), TNFRSF14 (coefficient = −0.438304706, *p* = 6.89E-25), SELP (coefficient = −0.425936122, *p* = 1.87E-23), ADORA2A (coefficient = −0.422734376, *p* = 4.30E-23), and BTLA (coefficient = −0.419675547, *p* = 9.45E-23) (Figure 7F, Supplementary Table 7). Violin plots were used to visualize the Wilcoxon test, which compared the distribution difference of 60 checkpoints between high-risk and low-risk groups (Figure 7G). The analysis revealed that 44 checkpoint genes exhibited a differential distribution.

To identify prognostic genes among the 60 checkpoints, we employed Cox analysis and KM analysis. Cox analysis indicated that LUAD prognosis was linked to 15 checkpoint genes (Figure 7H), while KM analysis demonstrated that 8 out of 60 checkpoint genes were capable of distinguishing LUAD prognosis significantly (Figure 7I). To further investigate the checkpoints that have a strong association with CoCuLncSig and significantly influence prognosis, we utilized conducted lollipop diagrams, violin diagrams, Cox analysis, and KM analysis, and employed a Venn diagram to intersect the results. The Venn diagram revealed that CD40LG, BTLA, SELP, IL2, CD28, and IL10 are the most noteworthy checkpoints (Figure 7J). In order to gain a better understanding of how these six checkpoint genes may impact immunotherapy, we utilized a heatmap visualization to assess their effectiveness (Figure 7K). Our analysis revealed that IL10, IL2, CD40LG, SELP, BTLA, and CD28 were ranked in decreasing order of immunotherapy scores. These findings suggest that investigating the potential crosstalk between CoCuLncSig and immunotherapy should be a focus of future exploration.

### Selecting potentially effective drugs and validating them for high-risk score LUADs

**Figure 8:**
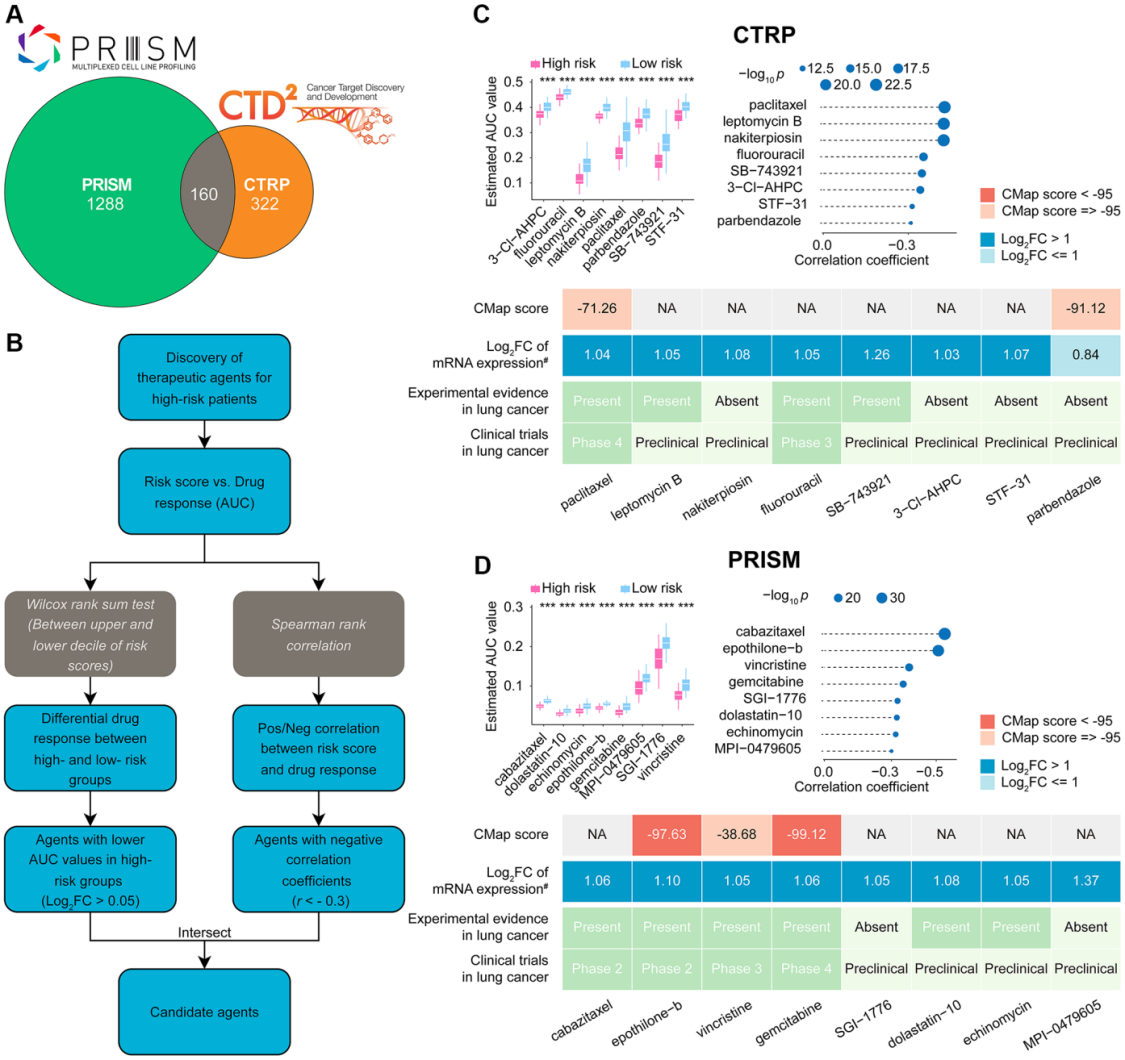
**Identification of candidate drugs for high CoCuLncSig risk score patients.** (**A**) The data for our drug prediction comes from the CTRP and PRISM databases, and the Venn diagram shows the compounds they include. (**B**) The flowchart shows the steps we explored in the drug databases of CTRP and PRISM, respectively, mainly including the Wilcoxon rank sum and the Spearman correlation statistical algorithms. (**C**) A collection of potential drugs has been discovered in the CTRP drug database. The top portion displays eight drug candidates that were identified through Spearman correlation and differential drug response analyses. The lower section presents the validation of the most encouraging LUAD therapeutics with high CoCuLncSig scores, using evidence from various sources. (**D**) The PRISM drug database has revealed a set of potential drug candidates. The top section displays eight drug candidates that were identified through Spearman correlation and differential drug response analyses. The lower section showcases the validation process for the most promising LUAD therapeutics with high CoCuLncSig scores, drawing on evidence from various sources. CoCuLncSig: copper homeostasis and cuproptosis regulated lncRNA signature; LUAD: lung adenocarcinoma; FC: fold change; ^#^: fold change differences of drug targets between tumor and normal tissue (> 0 represents up-regulated in tumor tissue); ^***^P-value < 0.001; In the analysis, a *P*-value of less than 0.05 was considered statistically significant, denoted by an asterisk.

After removing duplicate entries, the combined CTRP and PRISM datasets comprised 1770 unique compounds. One hundred sixty compounds were common to both datasets, as depicted in Figure 8A and summarized in Supplementary Table 8. By utilizing both CTRP and PRISM data, we integrated two distinct methodologies to pinpoint potential therapeutic agents for LUADs with elevated risk scores (Figure 8B). These approaches produced eight CTRP-derived agents (including paclitaxel, leptomycin B, nakiterpiosin, fluorouracil, SB-743921, 3-Cl-AHPC, STF-31, and parbendazole) (Figure 8C, upper) and eight PRISM-derived compounds (including cabazitaxel, epothilone-b, vincristine, gemcitabine, SGI-1776, dolastatin-10, echinomycin, and MPI-0479605) (Figure 8D, upper).

While 16 potential compounds demonstrated increased drug sensitivity in high-risk patients, relying solely on the above analysis is insufficient to conclude that these compounds are effective. Additional validation is necessary to provide more convincing evidence for our conclusion. Therefore, we proceeded with further validation analyses to evaluate the therapeutic potential of the 16 candidate compounds in high-risk score LUAD patients (Figure 8C lower, Figure 8D lower, Supplementary Table 9). According to the CMap analysis findings, two compounds, namely epothilone-b and gemcitabine, exhibited a CMap score of less than −95, indicating their potential as promising therapeutic options for LUAD. The fold-change values depicting elevated expression of drug target genes in tumor tissue as opposed to normal tissue suggest that these drug candidates have a higher likelihood of effectively treating LUAD. Additionally, we conducted a thorough search of the PubMed database https://www.ncbi.nlm.nih.gov/pubmed/) to find *in vivo* or *in vitro* studies supporting the efficacy of drug (Candidates, Furthermore, we utilized https://www.clinicaltrials.gov/ to search for lung cancer clinical trials related to the drug candidates. Our findings, presented in Figure 8C, Figure 8D, and Supplementary Table 9, indicate that epothilone-b and gemcitabine exhibit promising results for the treatment of high-risk score LUAD, based on their outstanding in silico and *in vitro* performance.

### CoCuLncSig is better than similar previous studies in survival predictions

**Figure 9:**
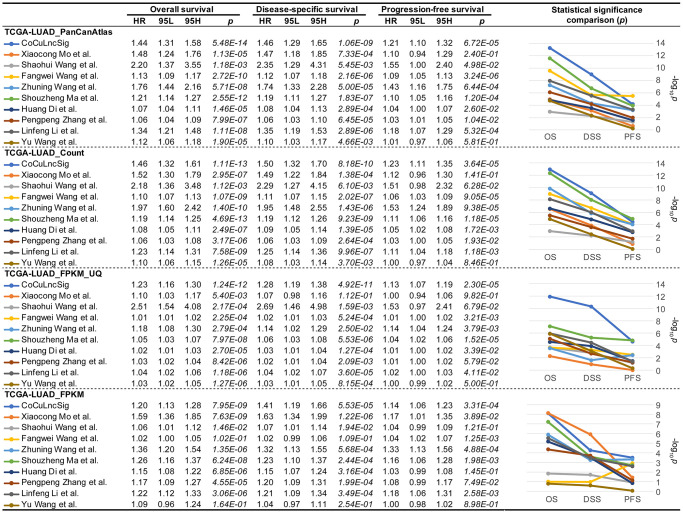
**Comparison of previous signatures [37–47] with CoCuLncSig by performing Cox regression analysis for overall, disease-specific, and progression-free survival using four formats of official TCGA data.** CoCuLncSig: copper homeostasis and cuproptosis regulated lncRNA signature.

In order to conclude whether our study is more robust than previous, we searched PubMed and initially eleven studies [37–47] were found (Table 3). However, Wei Ye et al. and Ran Chen et al. ‘s study did not provide the coefficient details of their signatures, which were excluded from our choices. Finally, nine studies [37–40, 42, 43, 45–47] were listed as candidates for comparison with our signature (Table 3). To compare previous signatures with ours, we performed Cox regression analysis for overall, disease-specific, and progression-free survival using four formats of official TCGA data (Figure 9), respectively. The analysis confirmed that CoCuLncSig has solid predictive ability in overall, disease-specific, and progression-free survival in four testing cohorts (*p* < 3.31e-04) (Figure 9). In particular, our signature occupies the first place in terms of *p* value in overall survival and disease-specific survival analysis of TCGA-LUAD_PanCanAtlas, TCGA-LUAD_Count, and TCGA-LUAD_FPKM_UQ, and progression-free survival analysis of TCGA-LUAD_FPKM (Figure 9). CoCuLncSig ranked 2nd in terms of *p* value in progression-free survival analysis of TCGA-LUAD_PanCanAtlas, TCGA-LUAD_Count, and TCGA-LUAD_FPKM_UQ, and overall survival and disease-specific survival analysis of TCGA-LUAD_FPKM (Figure 9). It’s worth noting that our signature didn’t rank third or worse in the comparisons. From the statistical significance comparison plots (Figure 9, right), we can see that the study that closest to our signature is from Shouzheng Ma et al., but they only ranked first in the progression-free survival analysis of TCGA-LUAD_Count and TCGA-LUAD_FPKM_UQ with a slight advantage, and in other comparisons in a later position.

**Table 3:** The characteristics of the similar categories of studies from predecessors [37–47].

**Authors**	**Published data**	**Journal name**	**Signature**	**Study PMID**
Xiaocong Mo et al.	July 22, 2022	*Frontiers in Oncology*	7-lncRNA signature	PMID: 35936736
Shaohui Wang et al.	August 30, 2022	*Frontiers in Pharmacology*	6-lncRNA signature	PMID: 36110528
Fangwei Wang et al.	September 1, 2022	*World Journal of Surgical Oncology*	16-lncRNA signature	PMID: 36050740
Wei Ye et al.	October 7, 2022	*MEDICINE*	3-lncRNA signature (no coefficient given)	PMID: 36221373
Zhuning Wang et al.	October 14, 2022	*Journal of Immunology Research*	8-lncRNA signature	PMID: 36281357
Shouzheng Ma et al.	October 31, 2022	*Translational Lung Cancer Research*	7-lncRNA signature	PMID: 36386454
Huang Di et al.	December 9, 2022	*MEDICINE*	10-lncRNA signature	PMID: 36626411
Ran Chen et al.	January 7, 2023	*Clinical and Translational Oncology*	5-lncRNA signature (no coefficient given)	PMID: 36609650
Pengpeng Zhang et al.	January 17, 2023	*Frontiers in Oncology*	7-lncRNA signature	PMID: 36733364
Linfeng Li et al.	February 11, 2023	*Scientific Reports*	7-lncRNA signature	PMID: 36774446
Yu Wang et al.	February 11, 2023	*Scientific Reports*	8-lncRNA signature	PMID: 36774418

### Validation of CoCuLncSig in human tissues and pan-cancer

**Figure 10:**
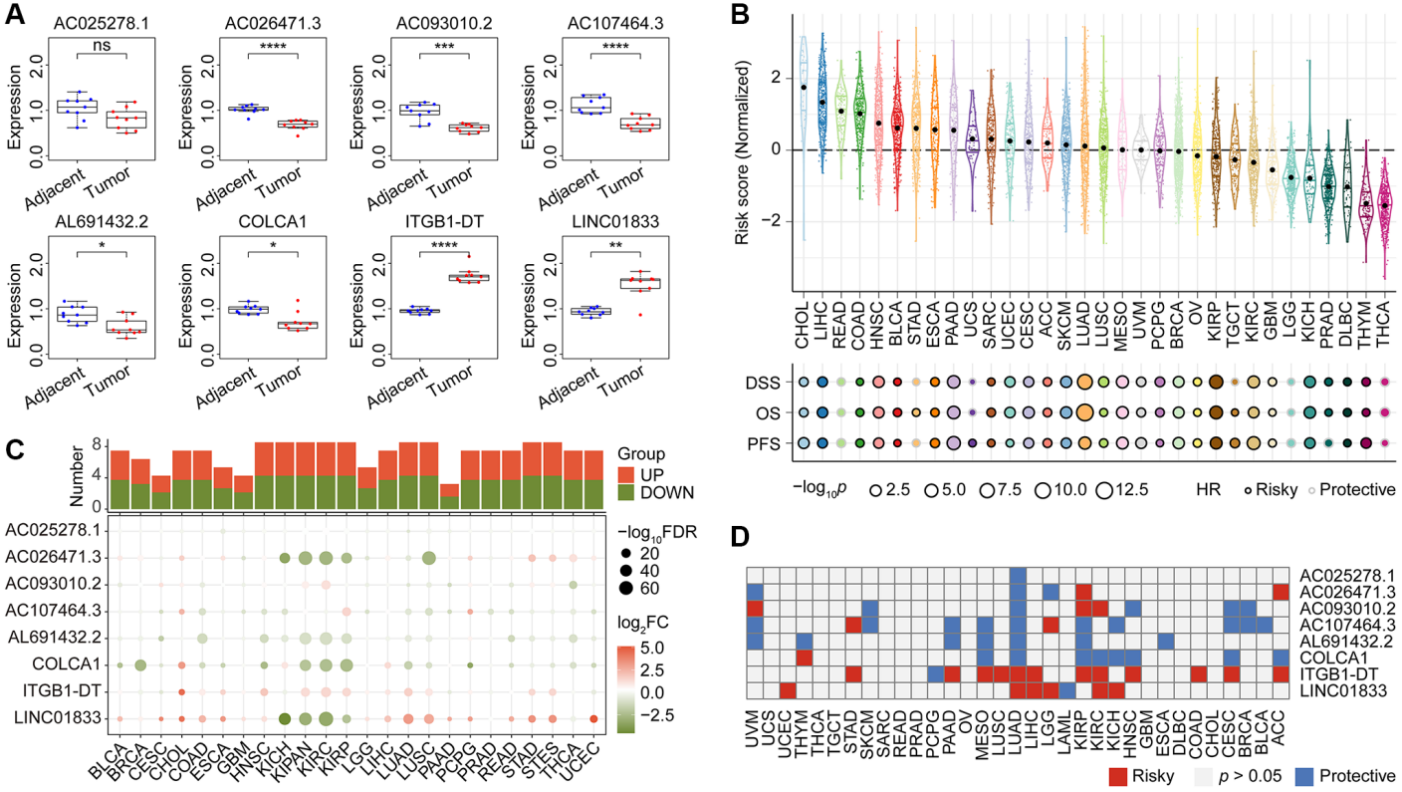
**The qRT-PCR validation of expression pattern of CoCuLncSig lncRNAs in human tissues and the prognostic potential assessments of CoCuLncSig by pan-cancer analysis.** (**A**) Boxplots showing the expression of CoCuLncSig lncRNAs measured by qRT-PCR in LUAD and adjacent tissues. (**B**) CoCuLncSig score distribution in pan-cancer and its impact on overall survival, disease specific survival, and progression free survival of each cancer type. (**C**) Heatmap depicts the variance in expression of CoCuLncSig lncRNAs between normal and tumor tissues across pan-cancer. The histogram at the top illustrates the count of genes exhibiting significant differential expression. Genes that are markedly upregulated and downregulated are identified with red and green markers, respectively. (**D**) Heatmap shows the prognostic ability of CoCuLncSig lncRNAs in pan-cancer. CoCuLncSig: copper homeostasis and cuproptosis regulated lncRNA signature.

Our qRT-PCR validation revealed differential expression of seven lncRNAs between LUAD and normal lung tissues (Figure 10A). Specifically, LINC01833 and ITGB1-DT were upregulated in LUAD tissues, while the remaining five lncRNAs were downregulated in cancer samples. Interestingly, our earlier analysis in Supplementary Figure 2 indicated that the upregulation of LINC01833 and ITGB1-DT in cancer tissues had a negative impact on LUAD prognosis. Conversely, our analysis in Supplementary Figure 2 confirmed that the downregulation of the other five lncRNAs had a protective effect on prognosis. The concurrence of the expression patterns and prognostic abilities further validates the efficacy of our developed CoCuLncSig and offers valuable insights for future in-depth studies. It is worth noting that we did not find differential expression of AC025278.1 in tumors and normal tissues. We speculate that it is due to the size of the sample or the reason for the race, but the specific reason remains to be explored.

To test the effectiveness of our CoCuLncSig in pan-cancer, we called the TCGA-LUAD_PanCanAtlas dataset. According to the calculation formula of the CoCuLncSig, the risk score distribution in pan-cancer was obtained, as shown in Figure 10B. We also called Cox regression to evaluate whether CoCuLncSig can impact the overall, disease-specific, and progression-free survival of pan-cancer. As a result, we found that our signatures were unfavorably influencing 25 of 32 kinds of cancer survival in terms of overall, disease- specific, and progression-free survival. What is very interesting is that our signature showed stable protectable effects on overall, disease-specific, and progression-free survival in READ, LGG, and THCA, which is worthy of further research. We analyzed the LUAD_PanCanAtlas dataset to explore the expression difference of the lncRNAs, as shown in Figure 10C. The plots hinted that the lncRNAs, AC026471.3, AL691432.2, COLCA1, ITGB1-DT, and LINC01833 ranked the different expression ability. The cancer types of KICH, KIPAN, KIRC, KIRP, and NSCLC may more strongly tell the eight lncRNAs’ differences between tumor and normal samples. The survival heatmap in Figure 10D showed that the ITGB1-DT and LINC01833 might have an unfavorable impact on most parts of the pan-cancer population. In contrast, the remaining lncRNAs mostly protected the outcomes. Our preliminary investigation into the potential of CoCuLncSig and eight lncRNAs in pan-cancer has provided valuable insights and highlighted their significance, thereby paving the way for further research in other cancers. However, there are still several obscure factors that require in-depth examination.

## DISCUSSION

Copper homeostasis refers to the maintenance of copper levels within the body at a stable and appropriate level [6]. This involves a delicate balance between copper uptake, distribution, utilization, and elimination [6]. Disruptions in copper homeostasis have been implicated in various human diseases, including cancer [48]. In cancer, copper is required for angiogenesis, the process of blood vessel formation that supplies tumors with nutrients and oxygen, and copper chelation therapy is being explored as a potential cancer treatment [48]. Cuproptosis refers to a recently discovered form of cell death triggered by copper, which is predominantly observed in cells that rely on oxidative phosphorylation as their primary metabolic process for energy production [6]. Numerous studies have indicated the crucial involvement of cuproptosis in cancer, particularly in its response to chemotherapy and radiation therapy [49, 50]. In some cases, cancer cells can become resistant to chemotherapy and radiation therapy by activating survival pathways that block apoptotic cell death. Furthermore, copper accumulation in cancer cells can also induce immunogenic cell death, which can trigger an immune response against the tumor [51]. This mechanism can potentially enhance the efficacy of immunotherapies and lead to better outcomes for cancer patients. Overall, understanding the roles of copper homeostasis and cuproptosis in cancer may lead to the development of new therapeutic strategies for cancer treatment, including the targeting of copper metabolism and the induction of cuproptosis as a means of enhancing the efficacy of existing treatments.

Further attention should be given to the study of joint modulators of copper homeostasis and cuproptosis in the progression and prognosis of LUAD disease, as currently there are no existing studies on the topic. Establishing an effective classifier for the treatment, prediction, follow-up, and other clinical work of LUADs is crucial due to the heterogeneity of patients and the significant difference in their prognostic results. Our study employed a combination of public database mining and experimental validation, as well as the latest concept of copper homeostasis and cuproptosis to establish the LUAD clinical model, CoCuLncSig. By utilizing advanced algorithms and statistics, we were able to confirm the broad applicability and effectiveness of our signature. CoCuLncSig targets immune function and key immune molecules, such as IL10, IL2, CD40LG, SELP, BTLA, and CD28. Our screening of 1770 compounds resulted in the identification of effective drugs for patients with high CoCuLncSig scores, and multidimensional validation of these drugs confirmed their efficacy. We also compared our CoCuLncSig and previous similar studies, described real-world expression patterns of the eight lncRNAs using qRT-PCR, and assessed the performance of the signature and its lncRNAs in pan-cancer.

Table 1 displays the signature we have devised in this study, comprising of eight lncRNAs: AL691432.2, AC093010.2, AC107464.3, AC025278.1, COLCA1, AC026471.3, LINC01833, and ITGB1-DT. In this study, validation through qRT-PCR confirmed that 7 out of 8 lncRNAs in our signature exhibited differential expression in tumor and normal tissue samples (Figure 10A). While public data mining indicated that AC025278.1 was also differentially expressed in tumor and normal, we did not observe a significant difference in the human tissue samples used in our qRT-PCR. This discrepancy may be due to differences in ethnic distribution between our samples and those in the public database. However, further research is required to ascertain the specific reasons. Based on the analysis depicted in Supplementary Figure 2 of this study, it was found that LUAD prognosis is adversely affected by LINC01833 and ITGB1-DT, whereas the remaining lncRNAs included in the CoCuLncSig have a protective effect on prognosis. Furthermore, pan-cancer analysis has identified AC026471.3, AL691432.2, COLCA1, ITGB1-DT, and LINC01833 as the top five lncRNAs in our signature exhibiting the most significant difference between pan-cancer tumors and normal tissue (Figure 10C). This finding may pique the interest of researchers and offer valuable insights for future investigations. Notably, our study highlights ITGB1-DT and LINC01833 as lncRNAs with a significant impact on the prognosis of more cancer types, warranting further attention in future research compare with other lncRNAs in the signature (Figure 10D). The lncRNA LINC01833 was a particular point of interest in this study due to its distinct differential expression in tumor and normal tissues, as well as its prognostic potential in cancer. LINC01833 is a lncRNA that is located on chromosome 2. It regulates various cellular processes, including cell proliferation, differentiation, and apoptosis. Multiple studies have demonstrated that LINC01833 can stimulate cancer cell proliferation, migration, and invasion in various cancer types [52, 53]. Specifically, LINC01833 can promote these activities by modulating the MiR-519e-3p/S100A4 axis and has shown promise as a biomarker for predicting cancer patient prognosis [52, 53]. In lung cancer tissues, LINC01833 is upregulated and has been linked to tumor progression and unfavorable prognosis [52, 53]. Nonetheless, additional research is necessary to gain a comprehensive understanding of how LINC01833 operates in lung cancer.

Considerable effort has been invested in validating the accuracy of our model, including a comparative analysis with previous studies. To accomplish this, we gathered all published studies [37–40, 42, 43, 45–47] that were similar to our research and evaluated their performance, along with our model, on the official TCGA data (Figure 9). By constructing Cox models and analyzing three different outcome endpoint data sets, namely OS, DSS, and PFS, we determined that our model generally outperformed previously published models. These findings suggest that our model has significant advantages over existing models. In addition, our study of pan-cancer has revealed that our signature negatively impacts the prognosis of 25 out of 32 cancers, specifically concerning OS, DSS, and PFS (Figure 10B). Taken together, our model not only has a stronger prognostic ability for LUAD, but also has predictive ability for many other types of cancer.

Tumor immunotherapy utilizes the immune system to combat cancer cells and is a form of cancer treatment. By tailoring treatment to an individual’s cancer type and immune system, immunotherapy has the potential to produce more effective and personalized outcomes [54]. Combining immunotherapy with other therapies like chemotherapy and radiation may enhance results [55]. Immunotherapy represents a highly promising and exciting area of oncology research, with the potential to transform cancer treatment and improve patient outcomes [55]. Immunotherapy’s primary obstacle is determining the suitability of a particular biomarker for a patient and devising a treatment plan that maximizes benefits [56]. This study sheds light on how immune checkpoints and CoCuLncSig are related, which can help determine the most effective immunotherapy methods for specific populations. The results of our study indicate that the CoCuLncSig score is strongly linked to TMB and TIDE, suggesting that CoCuLncSig could be a useful tool for guiding immunotherapy. Additionally, we identified six specific checkpoints (IL10, IL2, CD40LG, SELP, BTLA, and CD28) that are associated with CoCuLncSig, further supporting its potential role in guiding immunotherapy. In our selected cohort for immunotherapy, we evaluated the effectiveness of these checkpoints, and IL10, IL2, and CD40LG emerged as the top three checkpoints in terms of their immunotherapy capacity, listed in descending order (Figure 7K). IL-10 is a versatile cytokine that plays multiple roles in the immune system [57]. On one hand, it is required for the proper function of T-helper cells and for immune surveillance by T cells [58]. IL-10 also suppresses cancer-associated inflammation, making it a key player in the host’s fight against cancer [59]. However, IL-10 is also involved in tumor immune escape, as it is an immunosuppressive cytokine [60]. In addition, IL-10 is known for its potent anti-inflammatory properties and its ability to dampen immune responses to both self and foreign antigens [59]. IL-10 signaling blockade has been shown to enhance vaccine-induced T cell responses and prevent tumor growth [61]. NSCLC patients undergoing immunotherapy may benefit from monitoring IL-10 levels as a potential indicator of immune-related adverse events [62]. Activated CD4+ and CD8+ T cells secrete IL-2, a cytokine with important functions in regulating immune responses and promoting the expansion of T cells that recognize activating antigens [63]. IL-2 plays a critical role in the activation of the immune system and has been shown to be capable of mediating tumor regression as a monotherapy, making it a potential way to eradicate cancer [64]. In 1992, a laboratory-made form of IL-2 was the first immunotherapy approved to treat cancer, but its intravenous administration causes severe side effects, limiting its use [65]. However, in China, IL-2 has been approved since 1998 for the treatment of malignant pleural effusion [66]. Additionally, a meta-analysis has shown that IL-2 combination therapy is efficacious in treating NSCLC, improving overall survival without significant toxic reactions [67]. CD40LG, also known as CD154, is a protein that belongs to the TNF superfamily and is primarily found on activated T cells [68]. Initially, it was recognized for its critical role in T cell-dependent humoral responses by interacting with CD40, but later studies showed that it is also involved in cell-mediated immunity and inflammation [69]. CD154 can interact with CD40 alone or in combination with integrin receptors, contributing to the development of chronic inflammatory-related diseases [70]. Despite its involvement in disease development, CD154 has high potential for cancer treatment [69]. It activates anti-tumoral immunity and can induce apoptosis of tumor cells by engaging CD40 [69]. Animal models and clinical assessments have demonstrated the significant role of CD154 in cancer immunotherapy [71].

Due to the high degree of heterogeneity among individuals with LUAD, it is challenging to effectively treat all cases using a single approach [72]. Current treatment methods for advanced LUAD are not equipped with corresponding biomarkers, rely on population-based approaches, and have limited treatment outcomes [72]. The primary objective of this research is to identify personalized drug or small molecule therapeutic strategies for individual patients, which is crucial for optimizing therapeutic efficacy. Our developed CoCuLncSig model offers prognostic insights for patients with LUAD and can aid in precision oncology by guiding targeted therapies such as small-molecule drugs. We have pinpointed 16 drug candidates that demonstrate potential efficacy in the high-CoCuLncSig-scoring population. After several validations, we have found that Epothilone B and gemcitabine exhibit strong therapeutic potential with robust supporting evidence. Epothilone B has the potential to be an effective anticancer drug as it hinders cell division by interfering with microtubulin function [73]. Although microtubules are crucial for cell division, Epothilone B’s binding at the interface of two tubulin subunits hampers the general dynamics of microtubules [73]. Additionally, Epothilone B has been granted approval for treating metastatic breast cancer [74] and has exhibited encouraging clinical activity in a phase II trial conducted among NSCLC patients [75]. Gemcitabine is a chemotherapy drug used to treat different types of cancer, including bladder and breast cancer. In the 1980s, Larry Hertel synthesized gemcitabine for antiviral purposes, which later received FDA approval in 1998 as a treatment for NSCLC [76, 77]. Research involving gemcitabine monotherapy in over 400 patients has consistently reported response rates exceeding 20%, and it has been well tolerated in advanced NSCLC [78, 79]. Gemcitabine and Epothilone B are both widely recognized for their effectiveness in treating NSCLC [75, 78, 79]. However, research on Epothilone B in NSCLC is currently limited. Our study aims to contribute to the existing evidence by demonstrating the potential benefits of Epothilone B in our high-scoring patients, providing a fresh perspective for further investigation. Despite the considerable amount of data on gemcitabine in NSCLC patients, the drug’s effectiveness varies due to the heterogeneity of tumors [72]. Nevertheless, our study provides valuable guidance for the use of gemcitabine, indicating that high-scoring patients may be more responsive to the drug. Further exploration and research are needed to support our findings.

This study has limitations. Despite the validation of CoCuLncSig’s stable prognostic power in another large independent cohort and the confirmation of its stronger predictive ability through comparison with similar published studies, the data source in this study were solely obtained from open-access databases. Even though qRT-PCR conducted on human tissue samples has validated the differential expression of CoCuLncSig lncRNAs in tumor and normal tissues, the mechanisms behind this signature remain unclear. As such, more studies focusing on *in vivo* and *in vitro* experiments are urgently needed to provide further evidence supporting CoCuLncSig’s potential role in copper homeostasis, cuproptosis, and its clinical significance.

## CONCLUSION

The current research has developed a lncRNA signature, CoCuLncSig, associated with copper homeostasis and cuproptosis. This signature has been constructed to predict the prognosis of LUAD, and its stability and superiority have been confirmed by independent validation and comparison with previous studies. qRT-PCR assessment has also confirmed the differential expression of CoCuLncSig lncRNAs. The study highlights the significant role of CoCuLncSig in LUAD immune function and its potential for precision immunotherapy. Furthermore, our study identifies possible immunotherapeutic targets and drugs closely associated with CoCuLncSig, which could guide targeted therapy based on population characteristics. In conclusion, this study provides new insights into prognostic prediction and highlights the integration of immunotherapy personalization and precision therapy.

## Supplementary Materials

Supplementary Figures

Supplementary Tables 1, 2 and 4-7

Supplementary Tables 3, 8 and 9
