# Supplementary material for: Crosstalk between copper homeostasis and cuproptosis reveals a lncRNA signature to prognosis prediction, immunotherapy personalization, and agent selection for patients with lung adenocarcinoma
Source: Aging (Albany NY). 2023 Nov 26;15(22):13504–41. doi: 10.18632/aging.205281 (PMC10713389; doi:10.18632/aging.205281)
Supplement: Supplementary Figures [file aging-15-205281-s001.pdf]

## SUPPLEMENTARY FIGURES

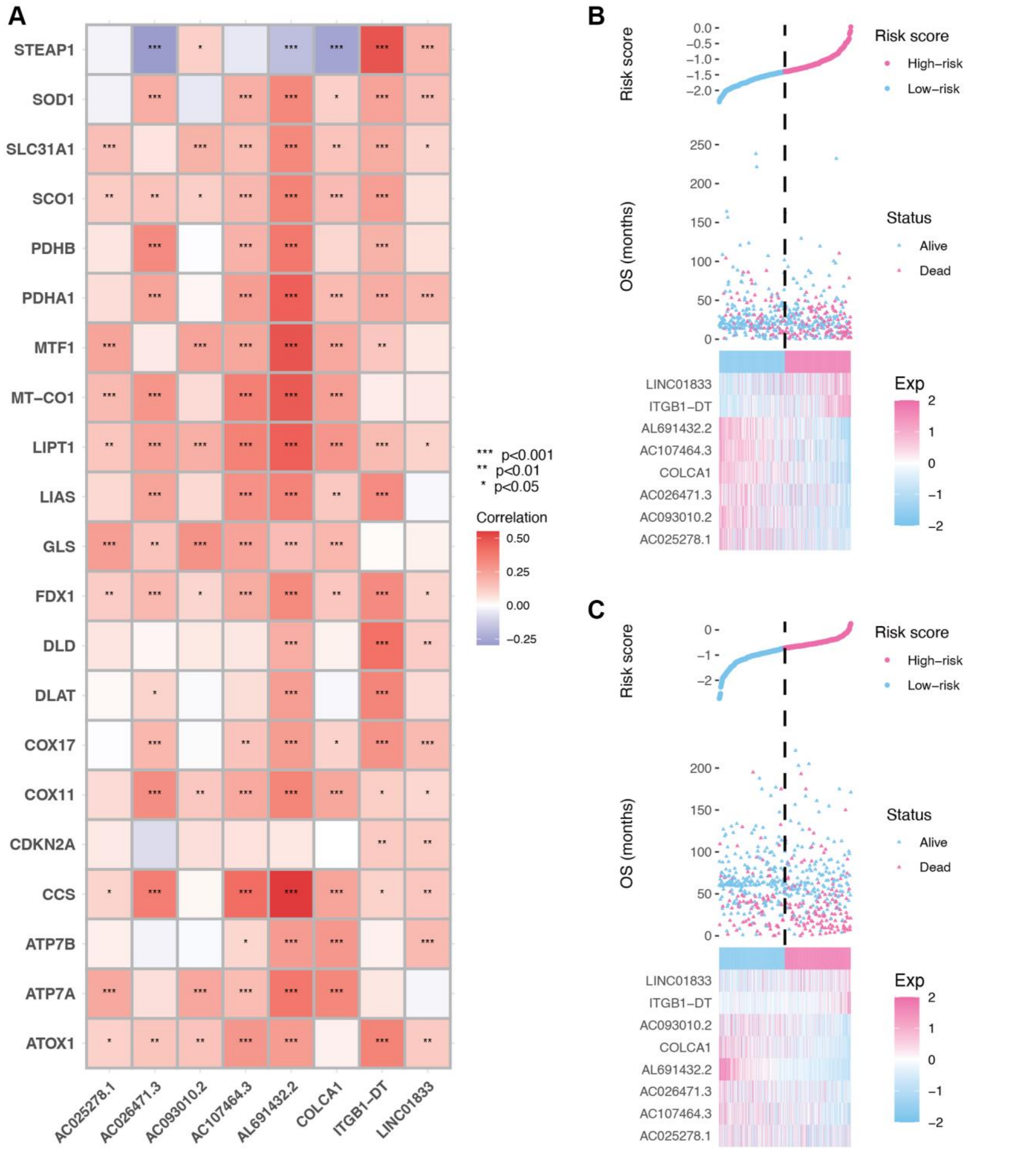


**Supplementary Figure 1. The profile of the signature lncRNAs.** (**A**) The heatmap shows the signature's eight lncRNAs' correlations with 21 copper homeostasis/cuproptosis-regulated genes. (**B**, **C**) The distributions of the risk score, survival status, survival time, and eight lncRNAs' levels for LUAD cases in the training cohort and validation cohort.

**A**

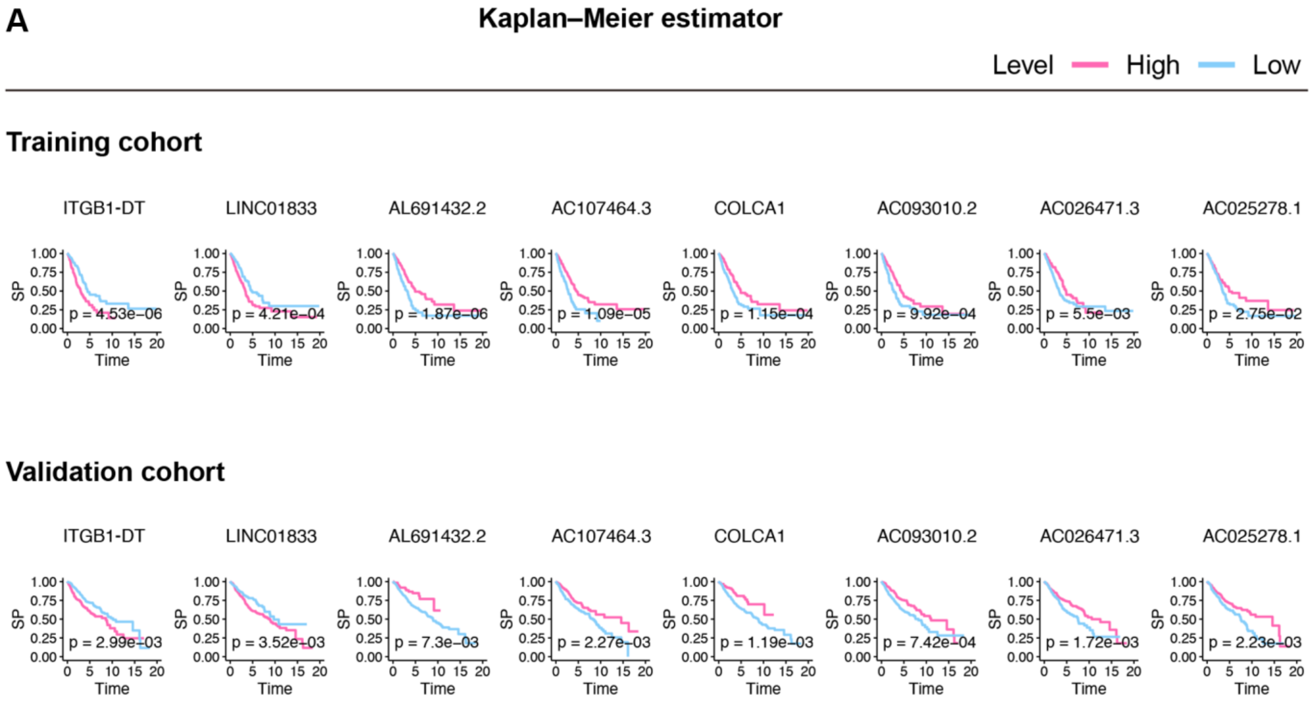


**B**

**Univariate Cox regression**

| Variable | HR | L95 | H95 | *p* |
|---|---|---|---|---|
| **Training cohort** | | | | |
| AC025278.1 | 0.4336 | 0.2332 | 0.8059 | 8.24e−03 |
| AC026471.3 | 0.7099 | 0.5558 | 0.9066 | 6.05e−03 |
| AC093010.2 | 0.4958 | 0.3338 | 0.7364 | 5.09e−04 |
| AC107464.3 | 0.5263 | 0.3776 | 0.7335 | 1.50e−04 |
| AL691432.2 | 0.3118 | 0.1909 | 0.5094 | 3.27e−06 |
| COLCA1 | 0.5885 | 0.4660 | 0.7432 | 8.46e−06 |
| ITGB1−DT | 1.7068 | 1.3467 | 2.1630 | 9.75e−06 |
| LINC01833 | 1.3259 | 1.1330 | 1.5517 | 4.38e−04 |
| **Validation cohort** | | | | |
| AC025278.1 | 0.1209 | 0.0179 | 0.8178 | 3.03e−02 |
| AC026471.3 | 0.7368 | 0.5631 | 0.9639 | 2.59e−02 |
| AC093010.2 | 0.5310 | 0.3664 | 0.7695 | 8.25e−04 |
| AC107464.3 | 0.2870 | 0.1100 | 0.7487 | 1.07e−02 |
| AL691432.2 | 0.7969 | 0.6520 | 0.9741 | 2.67e−02 |
| COLCA1 | 0.8001 | 0.6883 | 0.9300 | 3.68e−03 |
| ITGB1−DT | 1.5734 | 1.0012 | 2.4725 | 4.94e−02 |
| LINC01833 | 3.0241 | 1.3133 | 6.9634 | 9.31e−03 |

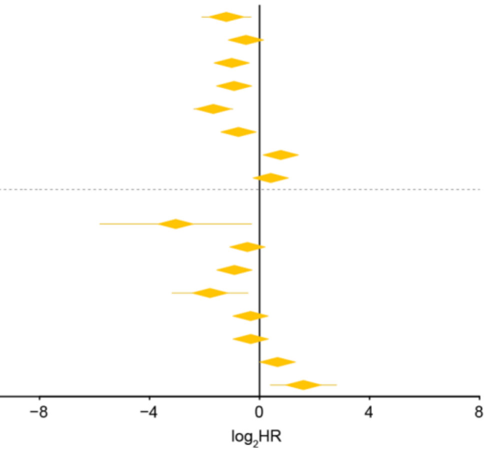


**Supplementary Figure 2.** The Kaplan-Meier analysis (**A**) and univariate Cox models (**B**) established in the studied cohorts testing the predictive ability of each of the eight signature lncRNAs. Patients were grouped based on their median risk score. The Kaplan-Meier method compared the survival difference between high and low-risk patients, and the log-rank test examined the significance.
