# Supplementary material for: Crosstalk between copper homeostasis and cuproptosis reveals a lncRNA signature to prognosis prediction, immunotherapy personalization, and agent selection for patients with lung adenocarcinoma
Source: Aging (Albany NY). 2023 Nov 26;15(22):13504–41. doi: 10.18632/aging.205281 (PMC10713389; doi:10.18632/aging.205281)
Supplement: Supplementary Tables 1, 2 and 4-7 [file aging-15-205281-s002.pdf]

SUPPLEMENTARY TABLES

Supplementary Table 1. 60 immune checkpoints obtained from previous studies.

ID	Type
VSIR	Inhibitory
VTCN1	Inhibitor y
VEGFB	Inhibitory
VEGFA	Inhibitory
TNFSF9	Stimulaotry
TNFSF4	Stimulaotry
TNFRSF9	Stimulaotry
TNFRSF4	Stimulaotry
TNFRSF18	Stimulaotry
TNFRSF14	Stimulaotry
TNF	Stimulaotry
TLR4	Stimulaotry
TIGIT	Inhibitory
TGFB1	Inhibitory
SLAMF7	Inhibitory
SELP	Stimulaotry
PRF1	Stimulaotry
PDCD1	Inhibitory
LAG3	Inhibitory
KIR2DL3	Inhibitory
KIR2DL1	Inhibitory
ITGB2	Stimulaotry
L4	Inhibitory
L2RA	Stimulaotry
L2	Stimulaotry
L1B	Stimulaotry
L1A	Stimulaotry
L13	Inhibitory
L12A	Inhibitory
L10	Inhibitory
FNG	Stimulaotry
FNA2	Stimulaotry
FNA1	Stimulaotry
0 0 1	Inhibitory
COSLG	Stimulaotry
ICOS	Stimulaotry
ICAM1	Stimulaotry
HMGB1	Stimulaotry
HAVCR2	Inhibitory
GZMA	Stimulaotry
ENTPD1	Stimulaotry
EDNRB	Inhibitory
CXCL9	Stimulaotry
CXCL10	Stimulaotry

CX3CL1	Stimulaotry
CTLA4	Inhibitory
CD80	Stimulaotry
CD70	Stimulaotry
CD40LG	Stimulaotry
CD40	Stimulaotry
CD28	Stimulaotry
CD276	Inhibitory
CD274	Inhibitory
CD27	Stimulaotry
CCL5	Stimulaotry
BTN3A2	Stimulaotry
BTN3A1	Stimulaotry
BTLA	Inhibitory
ARG1	Inhibitory
ADORA2A	Inhibitory

Supplementary Table 2. Seventy-two differentially expressed genes identified between five CoCu clusters.

ID
JMJD7-PLA2G4B
STARD9
AC020978.9
AC008764.2
GPRASP1
AC018665.1
TUBGCP6
AL031714.1
AL022328.1
AC009120.3
AL450263.1
AC009120.2
AC079336.5
AC116366.2
AL031709.1
CKS1B
STX16-NPEPL1
MASP2
CR559946.2
AL022328.3
AC004890.2
AC006001.3
LINC00106
MRPL15
AC005154.4
PLA2G4B
AC008897.1
AC116366.1

RN7SKP70
AC098851.1
AC003070.1
PFDN4
PWAR5
AL031600.1
TNS1
CGNL1
ZNF767P
AC108449.2
ARMC2-AS1
HERC2P2
COX5B
AL353588.1
ROMO1
GOLGA8R
LY6G5B
ERICH6B
SLC5A4
AC003957.1
ANAPC11
MRPL27
ATP5ME
C19orf53
ZNF540
GCC2
DEPDC1
ATP13A4
CENPX
NBEAL1
PHACTR2P1
CDC20
GADD45GIP1
COX8A
RP11-164P12.4
ESCO2
ATP5MC1
REL
BAZ2B
RP11-701H24.5
RSF1
ALKBH7
FMC1
SMIM29

Supplementary Table 4. The KM and Cox analysis screened the DELs and found 15 DELs meet our criteria for further LASSO.

ID	KM_pvalue	HR	HR_95L	HR_95H	Cox_pvalue
AC009120.2	0.038352	0.56660	0.389068	0.82515	0.003056
AC020659.1	0.048046	0.690863	0.535336	0.891574	0.004484
AC025278.1	0.038672	0.433554	0.233241	0.805899	0.008237
AC026471.3	0.015271	0.709857	0.555781	0.906646	0.006052
AC093010.2	0.001083	0.495782	0.333801	0.736366	0.000509
AC107464.3	0.004118	0.526263	0.377603	0.733451	0.00015
AL353622.1	0.04654	0.599205	0.426505	0.841837	0.003152
AL691432.2	0.000232	0.311819	0.190863	0.50943	3.27E-06
AP002026.1	0.016943	0.558334	0.385139	0.809413	0.002098
COLCA1	0.003529	0.588493	0.466014	0.743162	8.46E-06
CYP2B7P	0.00346	0.728416	0.610201	0.869532	0.000453
ITGB1-DT	0.000232	1.70676	1.346729	2.163041	9.75E-06
LINC01833	0.001948	1.325895	1.132956	1.551691	0.000438
TSPOAP1-AS1	0.033559	0.479305	0.315157	0.728947	0.000586
ZNF710-AS1	0.01814	0.550923	0.364675	0.832293	0.004626

Supplementary Table 5. Correlations identified between CoCuLncSig and the immune checkpoint blocker response signature.

ID	*r*	*p*	sign	absR	*r*Seg	*p*Seg
Alcoholism	0.317842	3.36E-13	pos	0.317842	0.5	<0.001
Base_excision_repair	0.458873	2.09E-27	pos	0.458873	0.5	<0.001
Cell_cycle	0.551933	3.32E-41	pos	0.551933	0.75	<0.001
Cytokine-cytokine_receptor_interaction	−0.174487	8.78E-05	neg	0.174487	0.25	<0.001
DNA_replication	0.488969	2.06E-31	pos	0.488969	0.5	<0.001
Fanconi_anemia_pathway	0.512925	6.71E-35	pos	0.512925	0.75	<0.001
Homologous_recombination	0.494461	3.45E-32	pos	0.494461	0.5	<0.001
MicroRNAs in cancer	0.380949	1.03E-18	pos	0.380949	0.5	<0.001
Mismatch_repair	0.518448	9.61E-36	pos	0.518448	0.75	<0.001
Nucleotide_excision_repair	0.463601	5.21E-28	pos	0.463601	0.5	<0.001
Oocyte_meiosis	0.574946	2.47E-45	pos	0.574946	0.75	<0.001
p53_signaling_pathway	0.536263	1.42E-38	pos	0.536263	0.75	<0.001
Progesterone-mediated_oocyte_maturation	0.597046	1.29E-49	pos	0.597046	0.75	<0.001

Proteasome	0.284891	8.62E-11	pos	0.284891	0.5	<0.001
Pyrimidine_metabolism	0.528358	2.69E-37	pos	0.528358	0.75	<0.001
RNA_degradation	0.396127	3.11E-20	pos	0.396127	0.5	<0.001
Spliceosome	0.492619	6.30E-32	pos	0.492619	0.5	<0.001
Systemic_lupus_erythematosus	0.266495	1.41E-09	pos	0.266495	0.5	<0.001
Viral_carcinogenesis	0.53229	6.29E-38	pos	0.53229	0.75	<0.001

Supplementary Table 6. Correlations identified between CoCuLncSig and the tumor immune cycle steps.

ID	*r*	*p*	sign	absR	rSeg	pSeg
Step_1_Release_of_cancer_cell_antigens	0.165305	0.000205	pos	0.165305	0.25	<0.001
Step_2_Cancer_antigen_presentation	−0.221413	5.71E-07	neg	0.221413	0.25	<0.001
Step_3_Priming_and_activation	−0.102557	0.021816	neg	0.102557	0.25	<0.05
Step_4_B_cell_recruiting	−0.162754	0.000258	neg	0.162754	0.25	<0.001
Step_4_Basophil_recruiting	0.314476	6.12E-13	pos	0.314476	0.5	<0.001
Step_4_CD4_T_cell_recruiting	−0.359971	9.63E-17	neg	0.359971	0.5	<0.001
Step_4_CD8_T_cell_recruiting	−0.004226	0.924908	neg	0.004226	0.25	ns
Step_4_Dendritic_cell_recruiting	−0.010722	0.810987	neg	0.010722	0.25	ns
Step_4_Eosinophil_recruiting	0.307629	2.02E-12	pos	0.307629	0.5	<0.001
Step_4_Macrophage_recruiting	−0.00854	0.848934	neg	0.00854	0.25	ns
Step_4_MDSC_recruiting	0.181006	4.68E-05	pos	0.181006	0.25	<0.001
Step_4_Monocyte_recruiting	0.024725	0.581244	pos	0.024725	0.25	ns
Step_4_Neutrophil_recruiting	0.180514	4.91E-05	pos	0.180514	0.25	<0.001
Step_4_NK_cell_recruiting	0.017849	0.690518	pos	0.017849	0.25	ns
Step_4_T_cell_recruiting	−0.151569	0.000673	neg	0.151569	0.25	<0.001
Step_4_TH1_cell_recruiting	0.085	0.05752	pos	0.085	0.25	ns
Step_4_TH17_cell_recruiting	−0.184121	3.44E-05	neg	0.184121	0.25	<0.001
Step_4_Th2_cell_recruiting	−0.086891	0.052168	neg	0.086891	0.25	ns
Step_4_TH22_cell_recruiting	−0.110149	0.013726	neg	0.110149	0.25	<0.05
Step_4_Treg_cell_recruiting	−0.100488	0.024637	neg	0.100488	0.25	<0.05
Step_5_Infiltration_of_immune_cells_into_tumors	−0.24098	4.88E-08	neg	0.24098	0.25	<0.001
Step_6_Recognition_of_cancer_cells_by_T_cells	0.102157	0.022339	pos	0.102157	0.25	<0.05
Step_?_Killing_of_cancer_cells	−0.003032	0.946074	neg	0.003032	0.25	ns

Supplementary Table 7. Correlations identified between CoCuLncSig and 60 immune checkpoint genes using the Pearson correlation coefficient.

Genes	cor	*p*value
ADORA2A	−0.422734376	4.30E-23
ARG1	−0.295481493	1.56E-11
BTLA	−0.419675547	9.45E-23
BTN3A1	−0.310095462	1.32E-12
BTN3A2	−0.248063616	1.90E-08
CCL5	−0.128477904	0.004007163
CO2?	−0.334905757	1.43E-14
CD274	−0.063518361	0.156134943
CD276	0.081440831	0.068829414

CO28	−0.370361517	1.06E-17
CO40	−0.192512989	1.46E-05
CO40LG	−0.504060342	1.41E-33
CO70	0.000305993	0.99455441
COBO	−0.296297197	1.37E-11
CTLA4	−0.306745955	2.36E-12
CX3CL1	−0.367033739	2.17E-17
CXCL10	0.04858284	0.278246571
CXCL9	−0.074801613	0.094767141
EDNRB	−0.352046529	4.92E-16
ENTPD1	−0.29612718	1.41E-11
GZMA	−0.094800545	0.034067326
HAVCR2	−0.185886992	2.88E-05
HMGB1	−0.051846811	0.247187926
ICAM1	−0.317627504	3.50E-13
ICOS	−0.26315788	2.30E-09
ICOSLG	−0.207058275	3.03E-06
001	−0.077338556	0.084059493
FNA1	0.047107532	0.293119117
FNA2	0.045016288	0.315096852
FNG	−0.025642875	0.567287385
L10	−0.191580669	1.61E-05
L12A	−0.143913472	0.001252
L13	−0.249069387	1.66E-08
L1A	0.124791538	0.005200089
L1B	−0.086803407	0.052405482
L2	−0.37708981	2.43E-18
L2RA	−0.110953556	0.013048004
L4	−0.366527201	2.41E-17
TGB2	−0.307862593	1.94E-12
KIR2DL1	−0.072705502	0.104413161
KIR2DL3	−0.061740117	0.168076463
LAG3	−0.150219132	0.000752406
PDCD1	−0.176593722	7.18E-05
PRF1	−0.191238621	1.67E-05
SELP	−0.425936122	1.87E-23
SLAMF7	−0.149814436	0.000777867
TGFB1	−0.248568211	1.77E-08
TIGIT	−0.254435724	7.91E-09
TLR4	−0.246665425	2.29E-08
TNF	−0.280494229	1.71E-10
TNFRSF14	−0.438304706	6.89E-25
TNFRSF18	−0.122333533	0.006164353
TNFRSF4	−0.192245883	1.50E-05
TNFRSF9	−0.095476734	0.032805198
TNFSF4	0.033482287	0.455050103
TNFSF9	0.04738705	0.290261217
VEGFA	−0.146499297	0.001018496

VEGFB	−0.220307603	6.52E-07
VSIR	−0.29320105	2.27E-11
VTCN1	−0.074043816	0.098168796
