# Supplementary material for: Crosstalk between copper homeostasis and cuproptosis reveals a lncRNA signature to prognosis prediction, immunotherapy personalization, and agent selection for patients with lung adenocarcinoma
Source: Aging (Albany NY). 2023 Nov 26;15(22):13504–41. doi: 10.18632/aging.205281 (PMC10713389; doi:10.18632/aging.205281)
Supplement: Supplementary Tables 3, 8 and 9 [file aging-15-205281-s003.pdf]

Supplementary Table 3. KEGG pathways identified by GSVA between two CoCu-DEG clusters. ID

logFC	AveExpr			t	P.Value	adj.P.Val	B
KEGG_PROTEASOME		-0.514214	-0.021759	-15.13745	4.46E-43	8.29E-41	87.1137
KEGG_DNA_REPLICATION		-0.482361	-0.022075	-12.71471	2.13E-32	1.99E-30	62.77938
KEGG_PARKINSONS_DISEASE		-0.378733	-0.019199	-12.24435	1.99E-30	1.23E-28	58.29693
KEGG_OXIDATIVE_PHOSPHORYLATION		-0.382917	-0.018117	-11.78579	1.51E-28	7.02E-27	54.0164
KEGG_CELL_CYCLE		-0.362096	-0.018108	-11.62397	6.81E-28	2.53E-26	52.52797
KEGG_PENTOSE_PHOSPHATE_PATHWAY		-0.315492	-0.018257	-11.55217	1.32E-27	4.10E-26	51.87143
KEGG_HUNTINGTONS_DISEASE		-0.327929	-0.017175	-11.41412	4.72E-27	1.25E-25	50.61573
KEGG_PYRIMIDINE_METABOLISM		-0.317194	-0.019189	-11.22323	2.70E-26	6.27E-25	48.89426
KEGG_ALZHEIMERS_DISEASE		-0.309114	-0.021003	-11.20395	3.21E-26	6.64E-25	48.72142
KEGG_PORPHYRIN_AND_CHLOROPHYLL_METABOLISM		-0.253645	-0.037944	-11.10722	7.71E-26	1.43E-24	47.85664
KEGG_GLUTATHIONE_METABOLISM		-0.268108	-0.026108	-10.93837	3.52E-25	5.95E-24	46.35824
KEGG_MISMATCH_REPAIR		-0.403678	-0.022138	-10.88529	5.65E-25	8.76E-24	45.89016
KEGG_GLYCOLYSIS_GLUCONEOGENESIS		-0.224116	-0.02313	-10.73678	2.11E-24	3.02E-23	44.58794
KEGG_GLYOXYLATE_AND_DICARBOXYLATE_METABOLISM		-0.313339	-0.024252	-10.1213	4.43E-22	5.89E-21	39.31444
KEGG_CITRATE_CYCLE_TCA_CYCLE		-0.351093	-0.021677	-10.10571	5.06E-22	6.28E-21	39.18348
KEGG_FOLATE_BIOSYNTHESIS		-0.296017	-0.033599	-10.05187	8.00E-22	9.30E-21	38.73245
KEGG_PROTEIN_EXPORT		-0.371446	-0.017624	-9.932249	2.20E-21	2.40E-20	37.73595
KEGG_RIBOSOME		-0.440127	-0.00877	-9.91832	2.47E-21	2.55E-20	37.62045
KEGG_CYSTEINE_AND_METHIONINE_METABOLISM		-0.221775	-0.031035	-9.547269	5.38E-20	5.27E-19	34.58441
KEGG_PURINE_METABOLISM		-0.214263	-0.02317	-9.482597	9.13E-20	8.49E-19	34.06345
KEGG_BIOSYNTHESIS_OF_UNSATURATED_FATTY_ACIDS		-0.262007	-0.026117	-9.46166	1.08E-19	9.59E-19	33.89533
KEGG_LINOLEIC_ACID_METABOLISM		0.226297	-0.020138	9.438958	1.30E-19	1.10E-18	33.71333
KEGG_PENTOSE_AND_GLUCURONATE_INTERCONVERSIONS		-0.250299	-0.044244	-9.409991	1.65E-19	1.33E-18	33.48155
KEGG_TASTE_TRANSDUCTION		0.148467	-0.013728	9.355618	2.56E-19	1.98E-18	33.04784
KEGG_RNA_POLYMERASE		-0.312704	-0.020275	-9.318326	3.46E-19	2.57E-18	32.75141
KEGG_BASE_EXCISION_REPAIR		-0.306597	-0.014459	-9.254452	5.78E-19	4.14E-18	32.24562

KEGG_ALANINE_ASPARTATE_AND_GLUTAMATE_METABOLISM	-0.192363	-0.030697	-9.05376	2.86E-18	1.97E-17	30.67269
KEGG_OOCYTE_MEIOSIS	-0.229943	-0.023443	-9.048804	2.97E-18	1.97E-17	30.63416
KEGG_NUCLEOTIDE_EXCISION_REPAIR	-0.305548	-0.014825	-8.723936	3.74E-17	2.38E-16	28.14226
KEGG_GALACTOSE_METABOLISM	-0.232092	-0.020419	-8.720505	3.84E-17	2.38E-16	28.11631
KEGG_RIBOFLAVIN_METABOLISM	-0.233498	-0.033088	-8.710556	4.15E-17	2.49E-16	28.04107
KEGG_ONE_CARBON_POOL_BY_FOLATE	-0.268353	-0.024557	-8.686701	4.98E-17	2.90E-16	27.86094
KEGG_HOMOLOGOUS_RECOMBINATION	-0.31657	-0.025433	-8.667472	5.78E-17	3.26E-16	27.71601
KEGG_P53_SIGNALING_PATHWAY	-0.208853	-0.022425	-8.521662	1.75E-16	9.59E-16	26.62483
KEGG_PATHOGENIC_ESCHERICHIA_COLI_INFECTION	-0.233907	-0.018415	-8.496289	2.12E-16	1.13E-15	26.43637
KEGG_ARGININE_AND_PROLINE_METABOLISM	-0.191944	-0.024934	-8.481079	2.38E-16	1.23E-15	26.32359
KEGG_PYRUVATE_METABOLISM	-0.219296	-0.024872	-8.473244	2.53E-16	1.27E-15	26.26556
KEGG_CARDIAC_MUSCLE_CONTRACTION	-0.164164	-0.027522	-8.34021	6.85E-16	3.35E-15	25.2864
KEGG_REGULATION_OF_AUTOPHAGY	-0.170373	0.02176	-8.335925	7.07E-16	3.37E-15	25.25506
KEGG_BASAL_TRANSCRIPTION_FACTORS	-0.266482	-0.022653	-8.14449	2.90E-15	1.35E-14	23.86735
KEGG_TERPENOID_BACKBONE_BIOSYNTHESIS	-0.281833	-0.025094	-8.133532	3.15E-15	1.43E-14	23.78867
KEGG_AMINOACYL_TRNA_BIOSYNTHESIS	-0.295927	-0.013441	-7.71481	6.34E-14	2.81E-13	20.84393
KEGG_AMINO_SUGAR_AND_NUCLEOTIDE_SUGAR_METABOLISM	-0.237755	-0.013869	-7.668198	8.78E-14	3.80E-13	20.5237
KEGG_ABC_TRANSPORTERS	0.164451	-0.017364	7.378422	6.47E-13	2.74E-12	18.56771
KEGG_SPLICEOSOME	-0.275831	-0.005334	-7.252119	1.52E-12	6.27E-12	17.73419
KEGG_FRUCTOSE_AND_MANNOSE_METABOLISM	-0.201241	-0.021361	-7.220111	1.88E-12	7.60E-12	17.52481
KEGG_RNA_DEGRADATION	-0.248671	-0.017427	-7.069034	5.11E-12	2.02E-11	16.54678
KEGG_N_GLYCAN_BIOSYNTHESIS	-0.231612	-0.018882	-7.028059	6.69E-12	2.59E-11	16.28445
KEGG_ALPHA_LINOLENIC_ACID_METABOLISM	0.166292	-0.013956	6.916643	1.38E-11	5.23E-11	15.5775
KEGG_STARCH_AND_SUCROSE_METABOLISM	-0.136802	-0.033695	-6.840882	2.24E-11	8.35E-11	15.10213
KEGG_DRUG_METABOLISM_OTHER_ENZYMES	-0.135286	-0.037578	-6.764966	3.64E-11	1.33E-10	14.63017
KEGG_PHENYLALANINE_METABOLISM	-0.171811	-0.031179	-6.645403	7.73E-11	2.76E-10	13.89581
KEGG_ASCORBATE_AND_ALDARATE_METABOLISM	-0.193024	-0.037442	-6.526082	1.62E-10	5.69E-10	13.17391
KEGG_BLADDER_CANCER	-0.165529	-0.019479	-6.375544	4.06E-10	1.40E-09	12.27899
KEGG_GLYCOSPHINGOLIPID_BIOSYNTHESIS_LACT	-0.14748	-0.029269	-6.329583	5.36E-10	1.81E-09	12.0093

O_AND_NEOLACTO_SERIES						
KEGG_PROGESTERONE_MEDIATED_OOCYTE_MATURATION	-0.164358	-0.021829	-6.27533	7.42E-10	2.46E-09	11.69311
KEGG_SYSTEMIC_LUPUS_ERYTHEMATOSUS	-0.142576	-0.020037	-6.236952	9.32E-10	3.04E-09	11.47084
KEGG_VIBRIO_CHOLERAE_INFECTION	-0.168031	-0.019696	-6.23434	9.47E-10	3.04E-09	11.45576
KEGG_VALINE_LEUCINE_AND_ISOLEUCINE_BIOSYNTHESIS	-0.227949	-0.023389	-6.160682	1.46E-09	4.61E-09	11.03261
KEGG_NICOTINATE_AND_NICOTINAMIDE_METABOLISM	-0.141007	-0.032357	-6.129131	1.76E-09	5.46E-09	10.85268
KEGG_STEROID_BIOSYNTHESIS	-0.197347	-0.018048	-5.882072	7.30E-09	2.23E-08	9.471481
KEGG_UBIQUITIN_MEDIATED_PROTEOLYSIS	-0.191416	-0.015605	-5.758078	1.46E-08	4.39E-08	8.796979
KEGG_GLYCINE_SERINE_AND_THREONINE_METABOLISM	-0.122122	-0.031326	-5.648679	2.68E-08	7.91E-08	8.212364
KEGG_TAURINE_AND_HYPOTAURINE_METABOLISM	0.14871	-0.019764	5.423848	8.99E-08	2.61E-07	7.042064
KEGG_SELENOAMINO_ACID_METABOLISM	-0.158409	-0.025363	-5.356174	1.28E-07	3.67E-07	6.698091
KEGG_PEROXISOME	-0.15393	-0.015619	-5.250336	2.23E-07	6.27E-07	6.167888
KEGG_CYTOSOLIC_DNA_SENSING_PATHWAY	-0.12486	-0.003506	-5.173037	3.31E-07	9.18E-07	5.786655
KEGG_AMYOTROPHIC_LATERAL_SCLEROSIS_ALS	-0.122543	-0.022299	-5.142284	3.86E-07	1.06E-06	5.636399
KEGG_GLYCOSAMINOGLYCAN_BIOSYNTHESIS_KERATAN_SULFATE	-0.153003	-0.016921	-5.089379	5.05E-07	1.36E-06	5.379803
KEGG_NON_HOMOLOGOUS_END_JOINING	-0.176978	-0.023987	-4.880825	1.41E-06	3.75E-06	4.391728
KEGG_BUTANOATE_METABOLISM	-0.12384	-0.027218	-4.812016	1.97E-06	5.15E-06	4.073984
KEGG_ASTHMA	0.183162	0.010845	4.697147	3.39E-06	8.76E-06	3.552743
KEGG_LYSINE_DEGRADATION	-0.143082	-0.022263	-4.528518	7.39E-06	1.88E-05	2.808519
KEGG_LIMONENE_AND_PINENE_DEGRADATION	-0.158602	-0.027811	-4.435954	1.12E-05	2.82E-05	2.410673
KEGG_EPITHELIAL_CELL_SIGNALING_IN_HELICOBACTER_PYLORI_INFECTION	-0.122232	-0.017631	-4.363857	1.55E-05	3.83E-05	2.106069
KEGG_PANCREATIC_CANCER	-0.130526	-0.018202	-4.340245	1.71E-05	4.20E-05	2.007313
KEGG_HEDGEHOG_SIGNALING_PATHWAY	0.097324	-0.015526	4.203503	3.10E-05	7.49E-05	1.445228
KEGG_ARACHIDONIC_ACID_METABOLISM	0.091883	-0.013796	4.192797	3.24E-05	7.74E-05	1.401926
KEGG_BASAL_CELL_CARCINOMA	0.100029	-0.012722	4.180701	3.42E-05	8.04E-05	1.35313
KEGG_PROPANOATE_METABOLISM	-0.134744	-0.022072	-4.172841	3.53E-05	8.21E-05	1.321494
KEGG_RENAL_CELL_CARCINOMA	-0.123927	-0.017196	-4.037009	6.24E-05	0.000143	0.783544

KEGG_SPHINGOLIPID_METABOLISM	-0.111109	-0.023932	-4.007061	7.06E-05	0.00016	0.667184
KEGG_BETA_ALANINE_METABOLISM	-0.10211	-0.029212	-3.991937	7.51E-05	0.000168	0.608732
KEGG_PRION_DISEASES	-0.105652	-0.015367	-3.965328	8.37E-05	0.000185	0.506394
KEGG_CALCIUM_SIGNALING_PATHWAY	0.080791	-0.014154	3.875831	0.00012	0.000263	0.166915
KEGG_GLYCOSPHINGOLIPID_BIOSYNTHESIS_GLOBO_SERIES	-0.117674	-0.017605	-3.853944	0.000131	0.000283	0.085006
KEGG_VALINE_LEUCINE_AND_ISOLEUCINE_DEGRADATION	-0.126087	-0.015296	-3.814449	0.000153	0.000327	-0.061689
KEGG_THYROID_CANCER	-0.111451	-0.019644	-3.742591	0.000203	0.000428	-0.32493
KEGG_VASCULAR_SMOOTH_MUSCLE_CONTRACTION	0.088778	-0.014647	3.626011	0.000317	0.000661	-0.741911
KEGG_PROSTATE_CANCER	-0.101859	-0.015892	-3.607693	0.000339	0.000701	-0.806292
KEGG_TRYPTOPHAN_METABOLISM	-0.080124	-0.024739	-3.559881	0.000405	0.00082	-0.972871
KEGG_MELANOMA	-0.081002	-0.02152	-3.559833	0.000406	0.00082	-0.973038
KEGG_DILATED_CARDIOMYOPATHY	0.089625	-0.01128	3.505	0.000496	0.000993	-1.161469
KEGG_REGULATION_OF_ACTIN_CYTOSKELETON	-0.089184	-0.014289	-3.494386	0.000516	0.001021	-1.197622
KEGG_GNRH_SIGNALING_PATHWAY	0.080396	-0.01471	3.474112	0.000556	0.001088	-1.266389
KEGG_GLYCEROLIPID_METABOLISM	-0.073167	-0.02185	-3.360201	0.000837	0.001621	-1.645651
KEGG_CIRCADIAN_RHYTHM_MAMMAL	0.109512	-0.014208	3.317002	0.000974	0.001868	-1.786316
KEGG_GLYCOSYLPHOSPHATIDYLINOSITOL_GPI_ANCHOR_BIOSYNTHESIS	-0.117702	-0.020031	-3.284277	0.001092	0.002073	-1.891716
KEGG_SMALL_CELL_LUNG_CANCER	-0.092642	-0.01771	-3.268762	0.001152	0.002165	-1.941335
KEGG_SNARE_INTERACTIONS_IN_VESICULAR_TRANSPORT	-0.110064	-0.015749	-3.263336	0.001174	0.002184	-1.958637
KEGG_FC_EPSILON_RI_SIGNALING_PATHWAY	0.080309	-0.012191	3.137232	0.001803	0.003321	-2.352928
KEGG_MATURITY_ONSET_DIABETES_OF_THE_YOUNG	-0.080496	-0.027066	-3.039014	0.002494	0.004548	-2.649669
KEGG_PANTOTHENATE_AND_COA_BIOSYNTHESIS	-0.081088	-0.023307	-3.02088	0.002646	0.004778	-2.70346
KEGG_CHRONIC_MYELOID_LEUKEMIA	-0.094547	-0.013148	-2.991591	0.002908	0.005202	-2.789684
KEGG_PHOSPHATIDYLINOSITOL_SIGNALING_SYSTEM	0.08179	-0.015796	2.987537	0.002947	0.00522	-2.801555
KEGG_INTESTINAL_IMMUNE_NETWORK_FOR_IGA_PRODUCTION	0.111844	0.005086	2.919113	0.003664	0.00643	-2.999566

KEGG_ANTIGEN_PROCESSING_AND_PRESENTATION	-0.087654	-0.003732	-2.87698	0.004182	0.007269	-3.119289
KEGG_GAP_JUNCTION	-0.067521	-0.015589	-2.858762	0.004426	0.007622	-3.170531
KEGG_PATHWAYS_IN_CANCER	-0.06903	-0.01402	-2.822529	0.004949	0.008445	-3.271512
KEGG_INSULIN_SIGNALING_PATHWAY	-0.073665	-0.019116	-2.798877	0.005321	0.008997	-3.336757
KEGG_ADHERENS_JUNCTION	-0.084387	-0.015501	-2.68394	0.007511	0.012585	-3.64622
KEGG_STEROID_HORMONE_BIOSYNTHESIS	-0.061449	-0.025548	-2.666908	0.007896	0.013114	-3.691007
KEGG_MTOR_SIGNALING_PATHWAY	-0.076537	-0.02197	-2.639115	0.008564	0.014097	-3.763492
KEGG_HEMATOPOIETIC_CELL_LINEAGE	0.083917	0.006532	2.616304	0.00915	0.014929	-3.822432
KEGG_NEUROACTIVE_LIGAND_RECEPTOR_INTERACTION	0.050318	-0.017116	2.588367	0.009916	0.016038	-3.893938
KEGG_RIG_I_LIKE_RECEPTOR_SIGNALING_PATHWAY	-0.064953	-0.008088	-2.577236	0.010237	0.016414	-3.92222
KEGG_ALLOGRAFT_REJECTION	0.106854	0.008692	2.467379	0.013935	0.022153	-4.194974
KEGG_HYPERTROPHIC_CARDIOMYOPATHY_HCM	0.059348	-0.01573	2.413485	0.016149	0.025456	-4.32454
KEGG_VASOPRESSIN_REGULATED_WATER_REABSORPTION	-0.072914	-0.0193	-2.349519	0.019175	0.029971	-4.47469
KEGG_ENDOCYTOSIS	-0.06806	-0.013258	-2.329077	0.020242	0.031374	-4.521844
KEGG_TIGHT_JUNCTION	-0.054888	-0.019904	-2.307966	0.021397	0.032891	-4.570113
KEGG_TOLL_LIKE_RECEPTOR_SIGNALING_PATHWAY	-0.064222	-0.00816	-2.283666	0.022798	0.034758	-4.625146
KEGG_TYROSINE_METABOLISM	-0.051171	-0.025545	-2.27135	0.023538	0.035594	-4.65282
KEGG_ETHER_LIPID_METABOLISM	0.050962	-0.019731	2.20885	0.027625	0.041438	-4.790992
KEGG_COLORECTAL_CANCER	-0.067008	-0.016633	-2.177044	0.029931	0.044538	-4.859854
KEGG_CELL_ADHESION_MOLECULES_CAMS	0.065402	3.14E-05	2.162964	0.031004	0.045768	-4.890027
KEGG_GLIOMA	-0.058378	-0.019677	-2.154866	0.031636	0.046333	-4.907292
KEGG_ADIPOCYTOKINE_SIGNALING_PATHWAY	-0.050811	-0.023257	-2.098514	0.036347	0.052817	-5.025679
KEGG_AUTOIMMUNE_THYROID_DISEASE	0.071209	0.02001	2.06734	0.039202	0.056524	-5.089846
KEGG_NOD_LIKE_RECEPTOR_SIGNALING_PATHWAY	-0.062573	-0.012028	-2.064077	0.039511	0.056532	-5.096508
KEGG_OLFACTORY_TRANSDUCTION	-0.044476	0.09938	-2.055628	0.040323	0.057252	-5.113709
KEGG_NOTCH_SIGNALING_PATHWAY	-0.060099	-0.009001	-1.963297	0.05015	0.070666	-5.297163
KEGG_JAK_STAT_SIGNALING_PATHWAY	0.045238	-0.009658	1.93845	0.053115	0.074282	-5.345114

KEGG_METABOLISM_OF_XENOBIOTICS_BY_CYTOCHROME_P450	-0.050642	-0.02922	-1.878295	0.060906	0.084542	-5.45871
KEGG_APOPTOSIS	-0.054734	-0.014625	-1.85402	0.064309	0.088603	-5.503552
KEGG_TYPE_II_DIABETES_MELLITUS	0.040334	-0.018781	1.845925	0.065478	0.08955	-5.518377
KEGG_PRIMARY_BILE_ACID_BIOSYNTHESIS	0.05005	-0.022434	1.842197	0.066022	0.089636	-5.525183
KEGG_FC_GAMMA_R_MEDIATED_PHAGOCYTOSIS	-0.053135	-0.01314	-1.809042	0.071028	0.095734	-5.585113
KEGG_ALDOSTERONE_REGULATED_SODIUM_REABSORPTION	0.043091	-0.015956	1.791273	0.073837	0.098804	-5.616791
KEGG_LYSOSOME	-0.05557	-0.006039	-1.707639	0.088307	0.117322	-5.761737
KEGG_LONG_TERM_DEPRESSION	0.035065	-0.019707	1.632528	0.103181	0.13611	-5.886073
KEGG_TGF_BETA_SIGNALING_PATHWAY	-0.041551	-0.018218	-1.606726	0.108728	0.142419	-5.927508
KEGG_PRIMARY_IMMUNODEFICIENCY	0.062369	-0.002013	1.597753	0.110712	0.144003	-5.941764
KEGG_HISTIDINE_METABOLISM	-0.036857	-0.028017	-1.510267	0.13159	0.16997	-6.076617
KEGG_NON_SMALL_CELL_LUNG_CANCER	-0.0445	-0.020469	-1.502669	0.133538	0.171297	-6.087973
KEGG_COMPLEMENT_AND_COAGULATION_CASCADES	0.036433	-0.006965	1.373747	0.170119	0.216407	-6.272011
KEGG_NEUROTROPHIN_SIGNALING_PATHWAY	-0.040801	-0.013307	-1.370812	0.171032	0.216407	-6.276011
KEGG_GLYCEROPHOSPHOLIPID_METABOLISM	0.030883	-0.018392	1.34768	0.178355	0.224127	-6.307233
KEGG_FATTY_ACID_METABOLISM	-0.040115	-0.022206	-1.343995	0.179543	0.224127	-6.312157
KEGG_O_GLYCAN_BIOSYNTHESIS	-0.034714	-0.020791	-1.314855	0.189145	0.234539	-6.350631
KEGG_SULFUR_METABOLISM	-0.03653	-0.020348	-1.282503	0.200244	0.246658	-6.392365
KEGG_ACUTE_MYELOID_LEUKEMIA	-0.040067	-0.012176	-1.275022	0.202877	0.248258	-6.401869
KEGG_WNT_SIGNALING_PATHWAY	-0.030975	-0.017947	-1.267064	0.205706	0.250074	-6.411917
KEGG_GLYCOSAMINOGLYCAN_DEGRADATION	-0.038493	-0.007653	-1.236695	0.216765	0.261807	-6.449691
KEGG_OTHER_GLYCAN_DEGRADATION	-0.042049	-0.011183	-1.229849	0.219316	0.262538	-6.458079
KEGG_ARRHYTHMOGENIC_RIGHT_VENTRICULAR_CARDIOMYOPATHY_ARVC	0.032139	-0.016561	1.227509	0.220193	0.262538	-6.460936
KEGG_NATURAL_KILLER_CELL_MEDIATED_CYTOTOXICITY	-0.034189	-0.008489	-1.209555	0.227005	0.268936	-6.482676
KEGG_CHEMOKINE_SIGNALING_PATHWAY	-0.033196	-0.008947	-1.184377	0.236811	0.278777	-6.512626
KEGG_ENDOMETRIAL_CANCER	-0.037408	-0.016541	-1.171321	0.242012	0.283109	-6.52791
KEGG_GLYCOSAMINOGLYCAN_BIOSYNTHESIS_CHONDROITIN_SULFATE	-0.035828	-0.008862	-1.130065	0.258976	0.301059	-6.5751

KEGG_ERBB_SIGNALING_PATHWAY	-0.030561	-0.01742	-1.089785	0.276318	0.319225	-6.619547
KEGG_FOCAL_ADHESION	-0.031469	-0.010454	-1.07472	0.283004	0.324931	-6.635759
KEGG_MELANOGENESIS	0.022711	-0.014975	1.042123	0.297844	0.339871	-6.670067
KEGG_ECM_RECEPTOR_INTERACTION	0.031832	-0.007888	0.99585	0.319791	0.36269	-6.716962
KEGG_VIRAL_MYOCARDITIS	0.031135	-0.003898	0.98424	0.32546	0.366164	-6.728395
KEGG_AXON_GUIDANCE	-0.024937	-0.012636	-0.981533	0.326792	0.366164	-6.731042
KEGG_GLYCOSPHINGOLIPID_BIOSYNTHESIS_GANGLIO_SERIES	-0.026971	-0.020059	-0.86752	0.386062	0.429985	-6.835915
KEGG_PROXIMAL_TUBULE_BICARBONATE_RECLAMATION	-0.017988	-0.027752	-0.762647	0.446024	0.493812	-6.920992
KEGG_T_CELL_RECEPTOR_SIGNALING_PATHWAY	0.021619	-0.012448	0.706841	0.479986	0.528268	-6.961812
KEGG_PPAR_SIGNALING_PATHWAY	-0.013925	-0.022642	-0.672778	0.501391	0.54858	-6.985207
KEGG_LEUKOCYTE_TRANSENDOTHELIAL_MIGRATION	-0.017487	-0.008996	-0.642911	0.520568	0.566232	-7.004771
KEGG_INOSITOL_PHOSPHATE_METABOLISM	0.018332	-0.015631	0.629428	0.529349	0.572435	-7.013313
KEGG_GRAFT_VERSUS_HOST_DISEASE	0.020338	0.000209	0.474316	0.635476	0.680197	-7.098571
KEGG_LEISHMANIA_INFECTION	0.016519	0.004473	0.473141	0.636314	0.680197	-7.099126
KEGG_DORSO_VENTRAL_AXIS_FORMATION	-0.013021	-0.022796	-0.465534	0.641746	0.680331	-7.102682
KEGG_RENIN_ANGIOTENSIN_SYSTEM	0.013426	-0.017151	0.460464	0.645378	0.680331	-7.10502
KEGG_DRUG_METABOLISM_CYTOCHROME_P450	0.011983	-0.026743	0.45763	0.647411	0.680331	-7.106316
KEGG_VEGF_SIGNALING_PATHWAY	0.006718	-0.016058	0.27616	0.782536	0.816138	-7.172651
KEGG_NITROGEN_METABOLISM	0.005951	-0.035188	0.2724	0.785424	0.816138	-7.173678
KEGG_TYPE_I_DIABETES_MELLITUS	0.008905	0.002698	0.236749	0.812946	0.840044	-7.182722
KEGG_GLYCOSAMINOGLYCAN_BIOSYNTHESIS_HEPARAN_SULFATE	-0.005731	-0.021545	-0.211348	0.8327	0.855702	-7.188393
KEGG_MAPK_SIGNALING_PATHWAY	-0.00414	-0.013437	-0.180958	0.856472	0.875295	-7.194334
KEGG_B_CELL_RECEPTOR_SIGNALING_PATHWAY	0.00525	-0.011728	0.166351	0.867946	0.881027	-7.196861
KEGG_RETINOL_METABOLISM	-0.004016	-0.031174	-0.161766	0.871554	0.881027	-7.197611
KEGG_CYTOKINE_CYTOKINE_RECEPTOR_INTERACTION	0.003252	-0.003649	0.12269	0.9024	0.907278	-7.203149
KEGG_LONG_TERM_POTENTIATION	-0.001866	-0.021488	-0.077012	0.938644	0.938644	-7.207695

Supplementary Table 8. Lists of drugs in CTRP and PRISM.

CTRP	PRISM
968	10-deacetylbaccatin
16-beta-bromoandrosterone	10-hydroxycamptothecin
1S,3R-RSL-3	12-O-tetradecanoylphorbol-13-acetate
3-Cl-AHPC	1-azakenpaullone
5-fluorouracil	1-naphthyl-PP1
A-804598	1-phenylbiguanide
AA-COCF3	2,3-DCPE
abiraterone	2-MeCCPA
ABT-199	2-methoxyestradiol
ABT-737	3-amino-benzamide
AC55649	3-deazaneplanocin-A
afatinib	3-fluorobenzylspiperone
AGK-2	4-(4-fluorobenzoyl)-1-(4-phenylbutyl)-piperidine
alisertib	4-chlorophenylguanidine
alvocidib	4E1RCat
AM-580	4-hydroxy-phenazone
apicidin	4-iodo-6-phenylpyrimidine
AT13387	4-methylgenistein
AT-406	4-methylhistamine
AT7867	4-pyrimidinecarbonitrile
austocystin D	5-fluorouracil
avicin D	5-hydroxymethyl-tolterodine
avrainvillamide	7-aminocephalosporanic-acid
axitinib	7-hydroxystaurosporine
AZ-3146	7-nitroindazole
azacitidine	8-bromo-cGMP
AZD1480	8-hydroxy-PIPAT
AZD4547	9-aminoacridine
AZD6482	A61603
AZD7545	A-674563
AZD7762	A-803467
AZD8055	abemaciclib
B02	abiraterone
bafilomycin A1	abiraterone-acetate
barasertib	ABT-702
bardoxolone methyl	ABT-737
Bax channel blocker	ABT-751
BCL-LZH-4	AC-264613
BEC	ACDPP
belinostat	acesulfame-potassium

bendamustine
betulinic acid
bexarotene
BI-2536
BIBR-1532
BIRB-796
birinapant
BIX-01294
blebbistatin
bleomycin A2
BMS-195614
BMS-270394
BMS-345541
BMS-536924
BMS-754807
bortezomib
bosutinib
BRD1812
BRD1835
BRD4132
BRD6340
BRD8899
BRD8958
BRD9647
BRD9876
BRD-A02303741
BRD-A05715709
BRD-A71883111
BRD-A86708339
BRD-A94377914
BRD-K01737880
BRD-K02251932
BRD-K02492147
BRD-K04800985
BRD-K09344309
BRD-K09587429
BRD-K11533227
BRD-K13999467
BRD-K14844214
BRD-K16147474
BRD-K17060750
BRD-K19103580
BRD-K24690302

acetazolamide
acetophenazine
acetylcysteine
acetylsalicylsalicylic-acid
acipimox
acivicin
ACY-1215
adapalene
adaprev
adarotene
adefovir-dipivoxil
ADL5859
AEE788
AEG3482
afatinib
afobazole
AG-14361
ajmaline
albendazole
alectinib
alexidine
alfacalcidol
alisertib
almorexant
alogliptin
alpelisib
altretamine
alvespimycin
alvocidib
AM-251
AM-404
AM-580
ambazone
amfenac
AMG-208
AMG-232
AMG458
AMG900
aminoglutethimide
aminopurvalanol-a
amisulpride
ammonium-lactate
amonafide

BRD-K26531177	amoxicillin
BRD-K27224038	amprolium
BRD-K27986637	amsacrine
BRD-K28456706	anagrelide
BRD-K29086754	anastrozole
BRD-K29313308	anguidine
BRD-K30019337	anisomycin
BRD-K30748066	AP1903
BRD-K33199242	AP26113
BRD-K33514849	apatinib
BRD-K34099515	aphidicolin
BRD-K34222889	AR-12
BRD-K34485477	AR-42
BRD-K35604418	arctigenin
BRD-K37390332	arcyriaflavin-a
BRD-K41334119	aripiprazole
BRD-K41597374	ARRY-334543
BRD-K42260513	artemisinin
BRD-K44224150	artesunate
BRD-K45681478	AS-1949490
BRD-K48334597	AS-703026
BRD-K48477130	aspirin
BRD-K49290616	AST-1306
BRD-K50799972	astemizole
BRD-K51490254	asymmetrical-dimethylarginine
BRD-K51831558	AT13387
BRD-K52037352	AT-7519
BRD-K55116708	AT7867
BRD-K61166597	AT-9283
BRD-K63431240	atenolol-(-)
BRD-K64610608	atenolol-(+/-)
BRD-K66453893	atipamezole
BRD-K66532283	atiprimod
BRD-K70511574	atorvastatin
BRD-K71781559	aurora-a-inhibitor-i
BRD-K71935468	AV-412
BRD-K75293299	avanafil
BRD-K78574327	AVL-292
BRD-K80183349	AVN-944
BRD-K84807411	axitinib
BRD-K85133207	AZ20
BRD-K86535717	AZ3146
BRD-K88742110	AZ-628

BRD-K90370028	AZ960
BRD-K92856060	azacitidine
BRD-K94991378	azalomycin-b
BRD-K96431673	azathioprine
BRD-K96970199	AZD1480
BRD-K97651142	AZD2014
BRD-K99006945	AZD2461
BRD-M00053801	AZD2858
brefeldin A	AZD3463
brivanib	AZD4547
BYL-719	AZD5363
C6-ceramide	AZD5438
cabozantinib	AZD6482
canertinib	AZD7762
CAY10576	AZD8055
CAY10594	AZD8330
CAY10618	AZD8931
CBB-1007	AZD9668
CCT036477	azilsartan
CD-1530	azithromycin
CD-437	azodicarbonamide
cediranib	azomycin-(2-nitroimidazole)
ceranib-2	aztreonam
cerulenin	bafetinib
Ch-55	baicalein
CHIR-99021	baicalin
chlorambucil	balapiravir
CHM-1	BAM7
CI-976	BAN-ORL-24
ciclopirox	barasertib
ciclosporin	barasertib-HQPA
CID-5951923	bardoxolone
CIL41	bardoxolone-methyl
CIL55	baricitinib
CIL55A	batimastat
CIL56	BAY-11-7082
CIL70	BAY-11-7085
cimetidine	BAY-87-2243
clofarabine	BAY-K-8644-(s)-(-)
COL-3	beclomethasone-dipropionate
Compound 1541A	bekanamycin
compound 1B	belinostat
Compound 23 citrate	bendamustine

Compound 7d-cis
CR-1-31B
crizotinib
cucurbitacin I
curcumin
cyanoquinoline 11
cyclophosphamide
cytarabine hydrochloride
cytochalasin B
dabrafenib
dacarbazine
darinaparsin
dasatinib
DBeQ
decitabine
dexamethasone
dinaciclib
docetaxel
doxorubicin
ELCPK
elocalcitol
entinostat
epigallocatechin-3-monogallate
erastin
erismodegib
erlotinib
etomoxir
etoposide
ETP-46464
EX-527
FGIN-1-27
fingolimod
FK-866
fluvastatin
foretinib
FQI-1
FQI-2
FSC231
fulvestrant
fumonisin B1
GANT-61
GDC-0879
GDC-0941
bendroflumethiazide
benfotiamine
bentazepam
benzethonium
benzocaine
benzydamine
benzyldimethylhexadecylammonium
bephenium-hydroxynaphthoate
berberine
beta-lapachone
betamethasone
betamethasone-dipropionate
beta-naphthol
betulinic-acid
bexarotene
BF2.649
BGT226
BI-2536
BI-78D3
BIBR-1532
BIBU-1361
BIBX-1382
bicalutamide
BI-D1870
bifemelane
BIIB021
bindarit
birinapant
bis(maltolato)oxovanadium(IV)
bisacodyl
bisindolylmaleimide-ix
bitopertin
BIX-01294
BIX-02188
blebbistatin-(-)
BMS-265246
BMS-345541
BMS-387032
BMS-599626
BMS-626529
BMS-690514
BMS-707035
BMS-754807

gefitinib
gemcitabine
GMX-1778
gossypol
GSK1059615
GSK2636771
GSK-3 inhibitor IX
GSK4112
GSK461364
GSK-J4
GW-405833
GW-843682X
HBX-41108
HC-067047
HLI 373
hyperforin
I-BET151
I-BET-762
ibrutinib
IC-87114
idelalisib
ifosfamide
imatinib
importazole
indisulam
IPR-456
isoevodiamine
isoliquiritigenin
isonicotinohydroxamic acid
ISOX
istradefylline
itraconazole
IU1
JQ-1
JW-480
JW-55
JW-74
KH-CB19
KHS101
Ki8751
Ko-143
KPT185
KU 0060648
BMS-986020
BNC105
BNTX
bortezomib
bosentan
bosutinib
brefeldin-a
brequinar
brigatinib
brilliant-green
brivaracetam
bromosporine
broxaldine
broxyquinoline
bruceantin
BTS-54505
bucladesine
bumetanide
buparlisib
buphenine
busulfan
butamben
buthionine-sulfoximine
butylscopolamine-bromide
BVD-523
BVT-948
BW-180C
BX-912
cabazitaxel
cabozantinib
caffeic-acid
caffeic-acid-phenethyl-ester
calcitriol
camptothecin
camylofine-chlorhydrate
candesartan
canertinib
canrenone
capecitabine
capsaicin
captamine
carbachol
carboplatin

KU-0063794	carboxyamidotriazole
KU-55933	carboxypyridine-disulfide
KU-60019	carfilzomib
KW-2449	cariporide
KX2-391	carmofur
L-685458	carmustine
lapatinib	carvedilol
LBH-589	CB-10-277
LE-135	CCG-50014
lenvatinib	CCMI
leptomycin B	CCT128930
linifanib	CCT129202
linsitinib	CCT137690
lomeguatrib	CD-437
lovastatin	cebranopadol
LRRK2-IN-1	cediranib
LY-2157299	cefdinir
LY-2183240	cefditoren-pivoxil
manumycin A	cefpiramide
marinopyrrole A	CEP-32496
masitinib	CEP-33779
Mdivi-1	CEP-37440
Merck60	cephalomannine
methotrexate	cetrimonium
methylstat	cetylpyridinium
MG-132	CGM097
MGCD-265	CGP-37849
MI-1	CGP-52411
MI-2	CGP-54626
mitomycin-c	CGS-15943
MK-0752	CGS-20625
MK-1775	CH5132799
MK-2206	chicago-sky-blue-6b
ML006	chidamide
ML029	CHIR-124
ML031	CHIR-98014
ML050	CHIR-99021
ML083	chlorambucil
ML162	chloramphenicol-palmitate
ML203	chlorhexidine
ML210	chlorindanol
ML239	chlormidazole
ML258	chlorogenic-acid

ML311	chloropyramine
ML312	chloroxine
ML320	chlorpropamide
ML334 diastereomer	chlorpyrifos
MLN2238	chlorquinaldol
MLN2480	cholecalciferol
momelotinib	chromocarb
MST-312	CI-844
myricetin	cibenzoline
myriocin	ciclesonide
N9-isopropylolomoucine	ciclopirox
nakiterpiosin	CID-5458317
narciclasine	cidofovir
navitoclax	cilostamide
necrostatin-1	cimetidine
necrostatin-7	cinacalcet
necrosulfonamide	cinalukast
nelarabine	cinnarazine
neopeltolide	cinromide
neratinib	ciprofibrate
neuronal differentiation inducer III	cisplatin
niclosamide	CL-218872
nilotinib	cladribine
nintedanib	clebopride
NPC-26	clenbuterol
NSC 74859	clindamycin-phosphate
NSC19630	clobetasol-propionate
NSC23766	clobutinol
NSC30930	clocortolone-pivalate
NSC48300	clofarabine
NSC632839	clofazimine
NSC95397	clofibrate
nutlin-3	clofoctol
NVP-231	clomifene
NVP-ADW742	clonazepam
NVP-BEZ235	clopamide
NVP-BSK805	clotrimazole
NVP-TAE684	CMPD-1
O-6-benzylguanine	CNX-2006
obatoclax	CNX-774
olaparib	cobicistat
oligomycin A	cobimetinib

omacetaxine mepesuccinate
OSI-027
OSI-930
ouabain
PAC-1
paclitaxel
palmostatin B
pandacostat
parbendazole
parthenolide
pazopanib
PD 153035
PD318088
PDMP
pevonedistat
PF-184
PF-3758309
PF-4800567 hydrochloride
PF-543
PF-573228
PF-750
PHA-793887
phloretin
PI-103
pifithrin-alpha
pifithrin-mu
PIK-93
piperlongumine
pitstop2
PL-DI
pluripotin
PLX-4032
PLX-4720
PRIMA-1
PRIMA-1-Met
PRL-3 inhibitor I
procarbazine
prochlorperazine
purmorphamine
PX-12
PYR-41
pyrazolanthrone
QS-11

colchicine
colforsin-daproate
colfosceril-palmitate
colistin-b-sulfate
combretastatin-A-4
costunolide
CP-673451
CP-724714
CP-945,598
CPI-1189
CPP
CR8-(R)
creatine
crenolanib
crizotinib
crizotinib-(S)
cromakalim
crystal-violet
CS-110266
CUDC-101
CUDC-907
curcumin
CX-4945
CX-5461
CYC116
cyclocytidine
cycloheximide
cyclophosphamide
cyclosporin-a
cyclosporine
cyclovalone
cyclovirobuxin-d
cyproheptadine
cyproterone-acetate
cyromazine
cyt387
CYT-997
cytarabine
cytochalasin-b
cytochlor
D-4476
D-64131
D-7193

quizartinib
QW-BI-011
R428
RAF265
regorafenib
Repligen 136
RG-108
rigosertib
RITA
RO4929097
ruxolitinib
salermide
saracatinib
SB-225002
SB-431542
SB-525334
SB-743921
SCH-529074
SCH-79797
selumetinib
semagacestat
serdemetan
SGX-523
SID 26681509
sildenafil
silmitasertib
simvastatin
sirolimus
sitagliptin
SJ-172550
skepinone-L
SKI-II
SMER-3
SN-38
SNS-032
SNX-2112
sorafenib
sotrastaurin
spautin-1
SR1001
SR8278
SR-II-138A
SRT-1720

dabrafenib
dacarbazine
dacinostat
daclatasvir
dacomitinib
dalcetrapib
danazol
danusertib
dapivirine
darapladib
darifenacin
dasatinib
daunorubicin
DCEBIO
decitabine
defactinib
deferasirox
deflazacort
deforolimus
dehydrocholate-acid
delanzomib
demecarium
deoxycorticosterone-acetate
dequalinium
desonide
desoxycortone
dexamethasone-acetate
dexrazoxane
dianhydrogalactitol
diaveridine
diazooxonorleucine
dichlorisone-acetate
dichloroacetate
dichlorvos
diclazuril
dicycloverine
dienogest
difluprednate
digitoxigenin
digitoxin
digoxigenin
digoxin
dihydroartemisinin

staurosporine
StemRegenin 1
STF-31
SU11274
sunitinib
SZ4TA2
tacedinaline
tacrolimus
tamatinib
tamoxifen
tandutinib
tanespimycin
temozolomide
temsirolimus
teniposide
TG-100-115
TG-101348
TGX-221
thalidomide
tigecycline
tipifarnib
tipifarnib-P2
tivantinib
tivozanib
topotecan
tosedostat
tozasertib
TPCA-1
trametinib
tretinoin
triazolothiadiazine
trifluoperazine
triptolide
tubastatin A
TW-37
UNC0321
UNC0638
VAF-347
valdecoxib
vandetanib
veliparib
VER-155008
vincristine
dihydroergocristine
dihydromyricetin
dimethisoquin
dinaciclib
dioscin
diphemanil
diphenyleneiodonium
dirithromycin
disulfiram
DMH1
docetaxel
dofetilide
dolastatin-10
dolutegravir
domiphen
domperidone
dorzolamide
dovitinib
doxercalciferol
doxifluridine
doxorubicin
doxycycline
DPI-201106
dronedarone
drospirenone
D-Serine
DU-728
DVD-111
E7449
ebastine
ecamsule-triethanolamine
echinomycin
efonidipine-monoethanolate
elacridar
elesclomol
eltrombopag
embelin
EMD-53998
emetine
endo-IWR-1
ENMD-2076
enocitabine
enoximone

vorapaxar
vorinostat
VU0155056
WAY-362450
WP1130
WZ4002
WZ8040
XL765
YK 4-279
YM-155
zebularine
ZSTK-474
entinostat
enzalutamide
epiandrosterone
epinastine
epinephrine
epirubicin
eplerenone
epothilone-a
epothilone-b
epothilone-d
eprinomectin
eprobemide
eprosartan
eptifibatide
EPZ-5676
equol
ER-27319
erastin
ercalcitriol
erdafitinib
erlotinib
erteberel
erythritol
estradiol
estradiol-benzoate
estradiol-cypionate
estramustine
estramustine-phosphate
estrone
ethacridine-lactate-monohydrate
ethinyl-estradiol
etofylline-clofibrate
etomoxir
etoposide
etoposide-phosphate
ETP-46464
evacetrapib
everolimus
evodiamine
EVP4593
exatecan-mesylate
exemestane
famciclovir

famotidine
favipiravir
fdcyd
felbamate
fenaclon
fenbendazole
fendiline
fenofibrate
fenoprofen
fenretinide
FERb-033
fexinidazole
FG-4592
filanesib
filgotinib
finafloxacin
FK-3311
FK-866
FK-888
fleroxacin
FLI-06
florfenicol
floxuridine
flubendazole
fludarabine
fludarabine-phosphate
fludroxycortide
flufenamic-acid
flumatinib
flumethasone
flumethasone-pivalate
fluocinolone-acetonide
fluorometholone
fluoromethylcholine
fluroxene
flutamide
fluticasone-propionate
fluvastatin
foretinib
formestane
forodesine
forskolin
fosbretabulin

foscarnet
FPH1-(BRD-6125)
FR-122047
FR-139317
frentizole
ftorafur
fudosteine
fulvestrant
G-1
galeterone
gambogic-acid
gamma-aminobutyric-acid
ganetespib
gastrodin
GDC-0068
GDC-0152
GDC-0349
GDC-0879
GDC-0941
GDC-0980
gefitinib
gemcitabine
genipin
genz-644282
gestrinone
GGTI-298
gidazepam
gilteritinib
gimeracil
ginkgolide-a
givinostat
golgicide-a
golvatinib
gossypol
GS-39783
GS-9973
GSK1070916
GSK1292263
GSK1838705A
GSK1904529A
GSK2110183
GSK2126458
GSK256066

GSK2656157
GSK2830371
GSK-3-inhibitor-IX
GSK429286A
GSK461364
GSK650394
GSK923295
GSK-J4
GTP-14564
guanidine
GW-3965
GW-405833
GW-441756
GW-501516
GW-583340
GW-788388
GW-842166
GW-843682X
GZD824
HA14-1
halcinonide
halobetasol-propionate
halofantrine
halofuginone
harringtonine
hesperadin
hexachlorophene
hexaminolevulinate
hexylresorcinol
HMN-214
homoharringtonine
homoquinolinic-acid
homosalate
hydrocortisone
hydroxytacrine-maleate-(r,s)
hydroxyurea
hyodeoxycholic-acid
hyoscyamine
hypericin
hypoestoxide
I-BET151
I-BET-762
ibrutinib

ibutilide
ICG-001
ICI-162846
icotinib
idarubicin
idasanutlin
idazoxan
idelalisib
idoxuridine
IDRA-21
idronoxil
IEM1754
ifosfamide
IKK-16
IKK-2-inhibitor-V
ilomastat
imatinib
imidapril
imiloxan
imiquimod
INC-280
indibulin
indiplon
indirubin
indisulam
indoprofen
ingenol-mebutate
INH1
inosine
iobenguane
iodipamide
iododexetimide
IOWH032
iproniazid
ipsapirone
irinotecan
isoetharine
isofloxythepin
isoflupredone-acetate
isometheptene-mucate
isopropyl-myristate
isoxsuprine
ispinesib

istradefylline
itraconazole
ivermectin
ixabepilone
ixazomib
ixazomib-citrate
JIB04
JK-184
JNJ-16259685
JNJ-26481585
JNJ-7706621
josamycin
JQ1
JTC-801
JTE-607
JZL-184
K-858
KD-023
KD025
kenpaullone
ketoconazole
ketoprofen
KF-38789
KI-16425
KI-8751
kifunensine
KP-1212
KPT-185
KPT-276
K-Ras(G12C)-inhibitor-6
k-strophanthidin
ku-0063794
KW-2449
KW-2478
KX2-391
KY02111
kynurenic-acid
L-798,106
lacitol
lafutidine
lanatoside-c
lanoconazole
lapatinib

lappaconite
lasalocid
latanoprost
latrepirdine
LB42708
LCL-161
LDN-212854
LDN-57444
LE-135
lenalidomide
lenvatinib
lerisetron
lestaurtinib
letrozole
leucovorin
levobunolol
levocarnitine
levocetirizine
levonorgestrel
LGX818
licochalcone-a
lidamidine
lidocaine
linalool
linifanib
linsitinib
litronesib
lomefloxacin
lomitapide
lonafarnib
lorazepam
lorlatinib
lovastatin
loxistatin-acid
lucitanib
lurasidone
LY2090314
LY2109761
LY2183240
LY2334737
LY2603618
LY2606368
LY2608204

LY2784544
LY2801653
LY2874455
LY3023414
LY303511
LY364947
LY456236
M-344
maprotiline
marimastat
masitinib
maxacalcitol
maytansinol-isobutyrate
mebendazole
mechlorethamine
meclizine
mefexamide
meglitinide
MEK1-2-inhibitor
MEK162
melengestrol-acetate
meloxicam
melphalan
menadione
mephenytoin
mepivacaine
mercaptopurine
mericitabine
merimepodib
mesna
mesoridazine
metaraminol
metatinib
methacycline
methiazole
methocarbamol
methotrexate
methoxyflurane
methscopolamine
methyldopa
methylphenidate
methylprednisolone
metoprolol

metoxibutropate
metronidazole
metyrapone
mevastatin
mexeneone
mexiletine
MG-132
MGCD-265
mibampator
mibefradil
midodrine
midostaurin
mifepristone
minocycline
mitomycin-c
mitoxantrone
MK-0773
MK-0812
MK-1775
MK-2206
MK-2461
MK-3207
MK-5108
MK-8245
MK-8745
ML133
MLN0128
MLN-8054
MNS-(3,4-Methylenedioxy-nitrostyrene)
mocetinostat
monensin
monobenzone
morin
motesanib
mozavaptan
MPI-0479605
MRS-1220
MTPG
mubritinib
mycophenolate-mofetil
mycophenolic-acid
N-acetylmannosamine
nadide

nadifloxacin
nafcillin
naftidrofuryl
naftifine
naloxone
naltrexone
NAN-190
nanchangmycin
napabucasin
naproxen
narasin
narlaprevir
navarixin
navitoclax
nefiracetam
nelarabine
nemonapride
nemorubicin
neratinib
nexturastat-a
NH125
niacin
niclosamide
nicorandil
nicotine
nilotinib
nilutamide
nimorazole
nintedanib
niraparib
niridazole
nisoxetine
nitarsone
nithiamide
nitisinone
nitrocaramiphen
nizatidine
nizofenone
NMS-1286937
NMS-873
NMS-E973
nobiletin
nocodazole

nomegestrol-acetate
nonoxynol-9
norepinephrine
norethindrone
norethindrone-acetate
noretynodrel
norfloxacin
norgestrel
novobiocin
NSC-23766
NSC-319726
NSC-3852
NSC-632839
NSC-663284
NSC-697923
NU6027
nutlin-3
NVP-AEW541
NVP-AUY922
NVP-BEZ235
NVP-BHG712
NVP-BVU972
NVP-TAE226
NVP-TAE684
obatoclax
obidoxime
octenidine
octopamine
odanacatib
olaparib
OLDA
oleanolic-acid
oleoylethanolamide
oligomycin-a
oltipraz
omeprazole
ONC201
oncrasin-1
ONX-0914
oprozomib
orantinib
oridonin
ornithine

orotic-acid
oseltamivir-phosphate
OSI-027
OSI-420
OSI-930
osimertinib
OTS167
OTX015
ouabain
oxaliplatin
oxazepam
oxcarbazepine
oxibendazole
oxiperomide
oxiracetam
oxonic-acid
oxprenolol
oxymatrine
oxymetazoline
oxyphencyclimine
oxyquinoline
P22077
P276-00
P5091
PAC-1
paclitaxel
pacritinib
palbociclib
paliperidone
palmatine-chloride
palmitoylethanolamide
palomid-529
panobinostat
papaverine
parachlorophenol
parbendazole
pardoprunox
paricalcitol
parthenolide
parthenolide-(-)
pazopanib
PCI-24781
PD-0325901

PD-153035
PD-168393
PD-173074
PD-184352
PD-198306
PD-318088
PD-407824
PD-98059
pelitinib
penfluridol
penicillamine-(D)
pentagastrin
pentamidine
pentostatin
pepstatin
peruvoside
PETCM
pevonedistat
PF-03758309
PF-03814735
PF-04136309
PF-04217903
PF-04457845
PF-04691502
PF-05212384
PF-3845
PF-477736
PF-4981517
PF-562271
PF-573228
PFI-1
PFK-015
PHA-665752
PHA-680632
PHA-767491
PHA-793887
PHA-848125
phenazone
phenelzine
pheniramine
phenylbutazone
phenylmercuric-acetate
phlorizin

PI-103
PI3K-IN-2
PI-828
pibenzimol
picolinic-acid
PIK-75
pilaralisib
pilocarpine
pindolol
piperacetazine
piperazine
piperine
pirenperone
piretanide
piroxicam
pitavastatin
PJ-34
PKI-179
plinabulin
PLX-4720
podophyllotoxin
polydatin
pomalidomide
ponatinib
posaconazole
poziotinib
PP-1
PP-121
PP-2
PP242
PPT
PQ-401
PR-619
practolol
pralatrexate
pranidipine
prazosin
prednisolone
prednisolone-acetate
prednisolone-hemisuccinate
prednisolone-tebutate
primaquine
proflavine-hemisulfate

propoxycaine
propranolol
proscillaridin-a
protirelin
PRT062070
PRT062607
PSB-1115
PSI-7976
PTC-209
pterostilbene
PU-H71
puromycin
pyrimethamine
pyrithione-zinc
pyritinol
pyroxamide
quinethazone
quinidine
quizartinib
R306465
R406
R547
rabeprazole
racecadotril
raclopride
radezolid
RAF265
raloxifene
raltitrexed
ramatroban
ramifenazone
ranitidine
refametinib
regorafenib
remoxipride
repaglinide
repsox
resatorvid
resiquimod
resminostat
RG108
RG2833
RGFP966

rheochrysidin
RI-1
ribavirin
ribitol
ribociclib
riboflavin
rifampin
rigosertib
riluzole
rimexolone
riociguat
RITA
ritodrine
RKI-1447
RN-1734
Ro-04-5595
Ro-10-5824
Ro-106-9920
Ro-4987655
Ro-90-7501
Ro-9187
rociletinib
romidepsin
roquinimex
RS-16566
RS-17053
RS-504393
RS-67506
RU-58841
rubitecan
rucinol
rutin
ruxolitinib
ryuvidine
S26948
salidroside
salinomycin
salvianolic-acid-B
salvinorin-a
sangivamycin
SAR131675
saracatinib
sarafloxacin

satraplatin
saxagliptin
SB-200646
SB-205384
SB-216641
SB-218078
SB-218795
SB-225002
SB-228357
SB-2343
SB-239063
SB-242235
SB-366791
SB-431542
SB-505124
SB-525334
SB-590885
SB-657510
SB-743921
SB-939
SC-12267
SC-144
SCH-58261
SCH-900776
sclareol
scriptaid
SCS
SDZ-WAG-994
secnidazole
securinine
selamectin
selinexor
selumetinib
semaxanib
se-methylselenocysteine
serdemetan
sertindole
sevelamer
sevoflurane
SGI-1027
SGI-1776
SIB-1757
SID-7969543

simvastatin
sirolimus
skepinone-l
SKF-96365
SKI-II
S-methylcysteine
SMI-4a
SN-38
SNS-314
SNX-2112
SNX-5422
sobetirome
sodium-glucoheptonate
sodium-stibogluconate
sodium-tanshinone-ii-a-sulfonate
sonidegib
sorafenib
sparfloxacin
spermidine
spermine
spiradoline
spironolactone
SR-27897
SR-33805
SR-57227A
SRT1720
STA-5326
stattic
stemregenin-1
STF-118804
streptozotocin
SU014813
SU3327
sufentanil
sulconazole
sulfabenzamide
sulfamethazine
sulfanilamide
sulfasalazine
sulmazole
sunitinib
suxibuzone
tacalcitol

tacedinaline
tacrolimus
tagatose
TAK-285
TAK-632
TAK-715
TAK-733
TAK-901
talazoparib
talmapimod
taltirelin
taltobulin
TAME
tamibarotene
tamoxifen
tanaproget
tandutinib
tanespimycin
tanshinone-i
TAS-103
taselisib
tasisulam
taurine
tazemetostat
TC1
tecastemizole
tedizolid
tedizolid-phosphate
telatinib
telotristat-ethyl
temazepam
temocapril
temoporfin
temozolomide
temsirolimus
teniposide
tenovin-6
tepoxalin
teprenone
terbutaline
terconazole
terfenadine
teriflunomide

teroxirone
tetrahydropapaverine
tetrahydrouridine
tetramethylthiuram-monosulfide
tetrandrine
tezacaftor
TG-02
TG100-115
TG-100572
TG-100713
TG-101209
TG-101348
TH-302
theophylline
thiamine
thiocolchicoside
thioguanine
thiomersal
thioproperazine
thiostrepton
thiram
thonzonium
tiagabine
tideglusib
tiletamine
tilmicosin
tiludronate
tioguanine
tiotidine
tioxolone
tipifarnib
tipiracil
tipranavir
tirapazamine
tivantinib
tivozanib
TMC-353121
TMS
tofogliflozin
topotecan
torasemide
torcetrapib
toremifene

torin-1
torin-2
tosedostat
tozasertib
TPCA-1
trametinib
tranilast
trans-4-Hydroxycrotonic-acid
trapidil
tremorine
trequinsin
triamcinolone
triamcinolone-acetonide
triamterene
triapine
trichostatin-a
triciribine
triclabendazole
trifluridine
trimebutine
triptolide
trolox
trometamol
tropisetron
trovafloxacin
tryptanthrin
TU-2100
tucatinib
TW-37
TWS-119
tyloxapol
tyrphostin-A9
tyrphostin-AG-1296
tyrphostin-AG-1478
tyrphostin-AG-494
tyrphostin-AG-99
U-0126
U-18666A
ubenimex
UH-232-(+)
UK-383367
ulipristal
UNBS-5162

UNC0631
UNC2250
uprosertib
uracil
uric-acid
uridine
usniacin-(+)
valnemulin
valrubicin
vandetanib
vanoxerine
vatalanib
VE-821
VE-822
vemurafenib
venetoclax
VER-49009
verubulin
vidarabine
vinblastine
vincamine
vincristine
vindesine
vinflunine
vinorelbine
vismodegib
VLX600
volasertib
voreloxin
vorinostat
voxtalisib
VS-4718
VTP-27999
VU0238429
VU0361737
VU0364770
VX-702
VX-765
warfarin
WAY-170523
WAY-600
WHI-P154
WP1066

WP1130
WYE-125132
WYE-354
WZ-3146
WZ-4002
WZ8040
xanomeline
XBD173
xilobam
XL388
XL-647
XL888
xylazine
Y-27632
Y-320
Y-39983
YM-155
YM-201636
YM-976
YO-01027
zaldaride
zaleplon
zardaverine
ziprasidone
ZK811752
ZK-93423
ZK-93426
ZLN005
ZM-306416
ZM-447439
zolantidine
zolmitriptan
ZSTK-474

Supplementary Table 9. List of potential therapeutic agents for LUAD patients with high-risk scores.

Drug name	Source	MOA (mechanism of action)	Targets (https://pubchem.ncbi.nlm.nih.gov/)	Clinical trials in lung cancer (https://clinicaltrials.gov/)	Experimental evdience in lung cancer (https://pubmed.ncbi.nlm.nih.gov/)	Drug targets mRNA Log2FC (normal and tumor)	CMap Score	Correlation Coefficient	Correlation P-value	AUC Log2FC	AUC Wilcox FDR
paclitaxel	CTRP	Tubulin inhibitor	PON1, Abcb1a, UGT1A1, UGT1A4, UGT2B10, UGT2B7, UGT1A6, BATF3, CD74, CD83, CYBB, Fcgr1, H2-Aa, H2-DMa, H2-M3, H2-T23, MAP3K14, PSMB9, TNF, TUBB4A, TUBB, TUBA3C, TUBA1B, TUBA4A, TUBB4B, TUBB3, TUBB2A, TUBB8, TUBA3E, TUBA1A, TUBA1C, TUBB6, TUBB2B, TUBB1, NRG1, SRC, NFE2, NULL, EPHA5, TRIM5, MET, CYP1B1, AURKA, CYP3A5, CXCL8, NOTCH1, SLCO1B3, STMN1, BDNF, KRAS, GSTP1, ABCC1, BCL2, PIK3CG, FOS, ABCC2, NR1I2, SOD2, ABCB1, GSTM1, PGP,	Phase 4	PMID: 9473739	1.043919	-71.26	-0.4274251	1.27E-23	0.47697598	2.02E-11

			FASN, JAK2, E2F1, DSCAM, CDK2, ABCG2, ERCC1, ATM, GSTT1, NAT2, BRCA2, EIF4EBP1, MYB, PDCD4, FNTB, FGD4, PTEN, MMP2, ERBB2, SPATA5, TP53, MAD1L1, BRCA1, EGFR, LIF, ABCC10, CDKN1A, AR, SMAD4, TUBA3D, PIK3CA, CYP19A1, RRM1, TLE3, ERCC2, ABCC3, TOP2A, DDIT3, CYP3A4, SOX10, BIRC5, CASP7, CASP3, OPRM1, SLC29A1, GMNN, CDKN2A, PDGFRA, IL2RA, EDNRB, SLCO1B1, SYK, RAF1, AKT1, MAP3K1, ACSS2, CYP3A7, CYP2C8, ABCB11, MAP4, MAPT, MAP2, Tlr4								
leptomycin B	CTRP	CRM1 inhibitor	AHR, AKT1, APAF1, AR, BACH1, BAX, BCL2, BCL2L1, BIRC2, BIRC3, BIRC5, BRCA1, BTG2, CASP1, GPX1, H2AX,	Preclinical	PMID: 28942004	1.046014	NA	-0.424487	2.73E-23	0.58634325	5.37E-10

			CCL2, CCNB2, CCND1, CCNE2, CDC25A, CDC25C, CDK1, CDKN1A, CHEK1, CHEK2, CXCL10, CXCL8, CYP3A4, DNMT1, E2F1, E2F3, EGFR, EI24, ESR1, FGF1, FOXO1, GADD45A, HAX1, HK2, ID3, IL1B, IL6, MAPK1, MAPK3, MDM2, MDM4, MYH4, NFKB1, NPPA, NR4A1, NR4A2, NUP62, PIDD1, PML, PRC1, PRKCA, PTTG1, RANBP1, RELA, RPS6KB2, RXRA, SESN1, SMAD2, SMAD3, SMAD4, SOD2, STAT4, STK4, TGFA, TGFB2, TNFRSF10B, TNFRSF10D, TP53, TP63, TXNDC5, WTIP, XPO1, XPO4								
nakiterpiosin	CTRP	Tubulin inhibitor	TUBA4A, TUBB	Preclinical	NA	1.076213	NA	-0.4242508	2.90E-23	0.12312712	8.53E-13

fluorouracil	CTRP	Thymidylate synthase inhibitor	TYMS, BAX, BBC3, BBS4, BBX, BCAR3, BCAS4, BCAT1, BCKDHA, BCL2, BCL2L1, BCL2L11, BCL2L12, BDH1, BDKRB2, BDNF, BEAN1, BECN1, BGLAP, BHLHE41, BID, BIRC2, BIRC3, BIRC5, BIRC6, BIRC7, BLM, BLVRB, BNIP1, BNIP3, BNIP3L, BOLL, BOP1, BORA, BRCA1, BRIX1, BTG2, BTG3, BTN3A3, BUB1, BUB1B, BUB3, C11orf24, C14orf132, C1orf35, C4BPB, CA12, CA14, CABYR, CAD, CALB2, CANX, CAP2, CASD1, CASP1, CASP10, CASP2, CASP3, CASP4, CASP6, CASP7, CASP8, CASP9, CAT, CAV1, CAV2, CBR3, CBS, CBX1, CCDC186, CCL2, CCN1, CCN2, CCN3, CCNA1, CCNA2, CCNB1, CCNB2, CCNC,	Phase 3		PMID: 32331446	1.045272	NA	-0.3531886	3.90E-16	0.06662838	1.88E-09

CCND1, CCND2,
CCND3, CCNE1,
CCNE2, CCNF,
CCNG1, CCNG2,
CCNJL, CD24, CD274,
CD34, CD38, CD40,
CD40LG, CD44,
CD52, CD63, CD8A,
CD8B, CD9, CDA,
CDC20, CDC25A,
CDC25B, CDC25C,
CDC34, CDC40,
CDC42EP1, CDC45,
CDC6, CDC73,
CDCA5, CDCA7,
CDCA8, CDH1, CDH5,
CDH6, CDIPT, CDK1,
CDK2, CDK5R1,
CDKN1A, CDKN1B,
CDKN1C, CDKN2A,
CDKN2AIP, CDKN2C,
CDKN3, CDX2,
CEBPB, CENPQ,
CENPU, CENPX,
CEP55, CER1, CES2,
CFLAR, CHEK1,
CHEK2, CHI3L1,
CHRNA4, CHST7,
CIAO1, CIDEB,
CITED2, CKB, CKS2,
CLCN3, CLDN2,
CLDN4, CLEC11A,
CLIC1, CLTC, cnk,

COCH, COL12A1,
COL1A1, COL1A2,
COL2A1, COL3A1,
COL4A2, COL5A2,
COL6A3, COX14,
COX5B, COX6C,
COX7C, CP, CPA4,
CPD, CPE, CPT1A,
CPXM1, CRABP1,
CRABP2, CRHBP,
CRIM1, CRK, CRMP,
CRYGS, CRYL1,
CRYZ, CSE1L, CSF1,
CSF2, CSNK1E,
CSRP1, CST7, CST8,
CSTF3, CTBP2,
CTNND2, CTPS1,
CTSB, CTSH, CTTN,
CUL4A, CUL4B,
CUL5, CUTC, CXCL1,
CXCL12, CXCL2,
DAPK1, DARS1,
CXCL8, CXCR1,
CXCR4, CYC1, CYCS,
CYFIP2, CYLD,
CYP1A1, CYP2A6,
Cyp2b1, CYP2F1,
CYP2S1, CYP3A4,
CYP51A1,
CYP51A1P2, CYTH3,
DBF4, DBNDD2, DCC,
DCK, DCLK1, DCT,
DCX, DCXR, DDAH2,

DDB2, DDIT3, DDIT4,
DDX39B, DDX4,
DENND2C, DEPDC7,
DES, DFFA, DHFR,
DHX15, DIABLO,
DICER1, DIMT1,
DKK1, DKK4, DLC1,
DLG1, DLK1, DNAH2,
DNER, DNMT3B,
DPAGT1, DPY19L1,
DPYD, DPYSL3,
DRAM1, DRD5, DST,
DTWD1, DTYMK,
DUSP1, DUSP14,
DUSP5, DUSP6, DUT,
E2F1, E2F2, E2F3,
E2F4, E2F8, EBP,
ECHDC3, ECHS1,
ECT2, EDIL3, EDN1,
EDNRA, EDNRB,
EEF1A1, FAM102A,
EFEMP1, EFHD1,
EFHD2, EFNB2,
EGFR, EGR1, EHD2,
EHF, EIF2AK3,
EIF2B2, EIF2S1,
EIF3H, EIF4E,
EIF4EBP1, EIF4H,
EIF5, EIPR1, ELMO2,
ELN, ELOA, ELOVL5,
EMC1, EML2, EMP3,
ENAH, ENDOG,
ENOSF1, ENTPD1,

ENTREP3, EOMES,
EP300, EP400,
EPAS1, EPB41L3,
EPHA2, EPHX1, EPO,
EPOR, EPPK1,
EPRS1, EPYC, ERAS,
ERBB2, ERBB3,
ERBB4, ERCC1,
ERCC2, EREG, ERN1,
ERP29, ESPL1, ETF1,
ETFB, ETS1, ETV2,
EXOSC10, F12, F2,
F3, F8, FABP7, FADD,
FADS1, FAH,
FAM107B, FAM114A1,
FAM171A1, FAM20C,
FAM234B, FAM76B,
FANCC, FANCG,
FANCI, FARS2, FAS,
Fasl, FASTK, FBLN1,
FBXO5, FKBP11,
FDXR, FEN1,
FERMT2, FGD6,
FGF2, FGF4, FGF5,
FGF7, FGFR2, FGG,
FHL2, FHL3, FIBCD1,
FILIP1L, FKBP1B,
FKBP5, FKBPL,
FLRT2, FLT1, FMOD,
FN1, FNBP1,
FNDC3B, FOLR1,
FOS, FOSL1, FOXA2,
FOXC1, FOXD3,

FOXG1, FOXL2,
FOXM1, FOXN3,
FOXO3, FPGS,
FTLP3, FUT1, FXYD3,
FYN, FZD2, G0S2,
GABRB3, GADD45A,
GADD45B, GADD45G,
GAL, GALNT3, GAMT,
GAPDH, GART,
GAST, GATA2,
GATA4, GATA6,
GCLM, GCSH, GDF1,
GDF15, GDF3, GEM,
GFAP, GFRA1,
GINS1, GINS2, GJA1,
GJC1, GLA, GLI1,
GLIPR1, GNB2, GNE,
GOLGA8A, GP1BB,
GPR132, GPR137C,
GPR35, GPR87, GPT,
GPX1, GPX2, GPX4,
GRAMD4, GRB7,
GRHL3, GRINA,
GSPT1, GSR, GSTA4,
GSTM1, GSTP1,
GSTT1, GTPBP4,
GUCY1B1, GYG2,
H1-3, H1f0, H2AX,
H2BC12, H3-7,
H4C14, H4C2, H4C3,
HACD1, HAND1,
HAND2, HAPLN2,
HBA1, HBB, Hbb-b2,

HBE1, HBZ, HDAC1,
HDAC2, HDAC3,
HDAC4, HDAC7,
HDAC9, HDGFL3,
HERPUD1, HES1,
Hexo1, HGF, HHLA3,
HIC2, HIF1A, HINT1,
HK2, HLA-F, HLA-G,
HM13, HMCES,
HMG1, HMG20B,
HMGN5, HMMR,
HMOX1, HNF1A,
HNF4A, HNRNPA1,
HNRNPA2B1,
HNRNPC, HNRNPF,
HNRNPH1, HNRNPL,
HOXA1, HOXB3,
HOXC11, HOXD1,
HPX, HRAS, HS6ST2,
HSD17B2, HSPA1B,
HSPA2, HSPA4,
HSPA5, HSPA8,
HSPB1, HSPB2,
HSPB8, HSPE1,
HTATIP2, HTR7,
HTRA1, IAPP, ID1,
ID2, ID2B, ID3, IDI1,
IER5, IFI16, IFI27L2,
IFI35, IFI6, IFIT1,
IFIT2, IFITM1, IFITM2,
IFITM4P, IFNA1,
IFNA2, IFNAR1,
IFNAR2, IFNB1, IFNG,

IGF1, IGF1R, IGFBP2,
IGFBP4, IGFBP5,
IGFBP6, IHH, IKBIP,
IKBKB, IL10, IL12B,
IL18, IL1A, IL1B,
IL1RN, IL27RA, IL3,
IL6, IL7, IMPA2,
IMPDH2, INKA2,
INPP5E, Ins2, INSIG1,
IQGAP2, IRAK1,
IREB2, IRF9, Irgm1,
ISG15, ISL1, ITGAV,
ITGB2, ITGB4, ITPR1,
ITSN1, JAG1, JMJD4,
JUN, JUNB, KANSL1,
KCNA5, KCNQ2,
KCTD12, KDM4B,
KDM5A, KDR, KEAP1,
KHK, KIAA0040,
KIF14, KIF15, KIF23,
KIF26B, KIF2C, KIF3A,
KIT, KITLG, KLF4,
KLF5, KLF6, KLHL24,
KLHL7, KLHL9,
KLK15, KLK6, KMT2E,
KNG1, KPNA2,
KPNA3, KRAS, KRT1,
KRT16, KRT17,
KRT18, KRT5, KRT6A,
KRT86, L1CAM,
L1TD1, LAMA1,
LAMB1, LAMC1,
LARP4B, LASP1,

LCMT2, LDHA, LDHB,
LEFTY1, LEFTY2,
LGALS3, LGALS3BP,
LGALS7, LGALS8,
LGR4, LGR5, LHX1,
LIFR, LIG1, LIMK1,
LIMK2, LIN28A, lin-29,
LIPH, LMF1, LMNA,
LMNB1, LMNB2,
LPAR6, LPO, LPXN,
LRATD1, LRBA, LRP8,
LSS, LXN, LY6G6C,
LY6G6D, LYPLA1,
M6PR, MACF1,
MAD2L1, MAD2L2,
MADD, MAF, MAFF,
MAGEA12, MAGEA2,
MAGEA4, MAGEA6,
MAGEB2, MAGED1,
MAGED4B, MALAT1,
MALT1, MAMDC2,
MAML2, MAN1A1,
MANEA, MAP1LC3B,
MAP2, MAP2K1,
MAP2K2, MAP3K1,
MAP3K20, MAP4K5,
MAPK1, MAPK13,
MAPK3, MAPK7,
MAPKAP1, MAPT,
MARCKS, MARS1,
MAST4, MBD4,
MBNL1, MBP,
MCCC2, MCF2, MCL1,

MCM10, MCM2,
MCM3, MCM4, MCM6,
MCM7, MCMBP,
MDFI, MDFIC, MDM2,
MDM4, MED13L,
MEF2B, MELK,
MEOX1, MESP1,
MESP2, METAP2,
METRN, MFN1,
MFN2, MGAT2,
MGMT, MICA, MICB,
MIF, MIR577, MKI67,
MKX, MLF1, MLH1,
MLLT11, MMP1,
MMP2, MMP3, MMP9,
MMS19, MNX1,
MORF4L2, MPC2,
MPIG6B, MPO, MPP7,
MPRIP, MR1, MRAS,
MSH2, MSX2, MT1E,
MT1M, MT1X, MTAP,
MTHFR, MTOR,
MTTP, MTUS1, MVP,
MXI1, MYB, MYBL2,
MYC, MYCL, MYCN,
MYD88, MYF5, MYL4,
MYL9, MYLIP,
MYO1E, MYO6,
MYOD1, MYOF,
NAF1, NAIP, NANOG,
NASP, NBL1, NBN,
NBPF10, NCAPG,
NCL, NCOR1,

NDUFA10, NDUFA4,
NDUFA6, NDUFAB1,
NDUFAF1, NDUFAF3,
NDUFAF4, NDUFB10,
NDUFB2, NDUFB9,
NDUFC1, NDUFV1,
NDUFV3, NEDD4,
NEK1, NEK2, NEK4,
NEK6, NEK9, NES,
NETO2, NEUROD1,
NEUROG2, NFE2L2,
NFE2L3, NFIB, NFIC,
NFKB1, NFKBIA,
NFYA, AARS1,
ABCB1, ABCB6,
ABCC1, ABCC11,
ABCC2, ABCC5,
ABCC6, ABCG2,
ABHD12B, ABI1, ABI2,
ACAD9, ACADL,
ACE2, ACKR3,
ACOT13, ACOT7,
ACTB, ACTC1,
ACTG2, ACTL8,
ACY1, ADAM10,
ADAM3A, ADAMTS9,
ADCY9, ADD3,
ADGRG1, ADGRG6,
ADGRL2, ADIRF,
ADM, ADSS2, AFP,
AGAP3, AGR2,
AHNAK, AIFM1,
AKAP12, AKR1B10,

AKR1C1, AKR1C2,
AKR1C3, AKT1, AKT2,
ALB, ALCAM,
ALDH1A1, ALDH1A3,
ALDH3A2, ALG3,
ALKBH8, AMFR,
AMPD2, AMT, ANG,
ANGPT1, ANGPT2,
ANGPTL2, ANGPTL3,
ANGPTL4, ANKRD1,
ANKRD11, ANKRD36,
ANKRD36B, ANLN,
ANP32E, ANXA1,
ANXA2, ANXA3,
ANXA4, AP2S1,
AP3S1, AP4S1,
APAF1, APBA2,
APBB2, APEX1,
APOB, APOBEC3C,
APOC1, APOC3,
AQP12B, AQP6,
AQP9, AREG, ARF1,
ARF3, ARFGAP1,
ARHGDIB, ARID5B,
ARL4C, ARL6IP5,
ARMCX1, ARPC3,
ASAH1, ASS1, ATF3,
ATF4, ATF6, ATG12,
ATOH8, ATP5F1D,
ATP5PB, ATP5PF,
ATP5PO, ATP6,
ATP7B, ATP9A,
ATXN10, AURKA,

AVEN, BAD, BAG3,
BAK1, BARX2, NFYB,
NINJ1, NIPSNAP2,
NKAIN1, NKIRAS1,
NME1, NME4,
NODAL, NOG, NOM1,
NOS2, NOS3,
NOTCH1, NPM1,
NPM3, NPPA, NQO1,
NR1D2, NR4A1,
NR5A2, NR6A1,
NRG1, NRIP1, NRP1,
NT5C1A, NT5E,
NT5M, NTN4, NTRK2,
NTS, NTSR1,
NUCKS1, NUPR1,
OAS2, ODC1,
OGFRL1, OLFM1,
OLIG2, OLIG3, OPTN,
ORC3, ORC6, OTX2,
OXCT1, P4HB,
PAFAH1B1, PAICS,
PAIP1, PANK1,
PAQR8, PARD3,
PARP1, PARP14,
PAX3, PAX6, PBX3,
PCDHA5, PCLAF,
PCNA, PCOLCE2,
PDAP1, PDCD1LG2,
PDCD4, PDCD6IP,
PDF, PDIA4, PDLIM5,
PDXK, PDXP,
PECAM1, PEG10,

PER1, PER2, PFN2,
PGLYRP1, PGR,
PHIP, PHLDA3,
PHYH, PI4KB, PIR,
PISD, PLA2G4A,
PLAT, PLAU, PLCG1,
PLEKHB1, PLEKHO1,
PLIN2, PLIN3, plk,
PLK1, PLK2, PLK3,
PLOD2, PLP2,
PLRG1, PLSCR1,
PMAIP1, PMF1,
PMS2P4, PNMA2,
PNP, PNPLA6,
PNRC1, PODXL,
POLA1, POLA2,
POLE2, POLG,
POSTN, POU5F1,
PPA2, PPARG,
PPFIBP1, PPIE,
PPM1D, PPP1CA,
PPP1CC, PPP1R15A,
PPP1R17, PPP1R1B,
PPP1R2, PPP2CB,
PPP3CA, PRC1,
PRDM14, PREPL,
PRIM1, PRKAG2,
PRKCI, PRKCZ,
PRKDC, PRNP,
PROC, PROCR,
PRODH2, PROM1,
PRPF18, PRPS1,
PRPS2, PRR13,

PRRG1, PRRX1,
PRSS2, PSG9,
PSMB10, PSMC3IP,
PSRC1, PTCH1,
PTEN, PTGR1, PURA,
PURG, PTGS2, PTK2,
PTPRK, PTTG1,
PTTG3P, PUM1,
PVT1, PWP2, PXMP2,
QSOX1, R3HDM2,
RAB11A, RAB20,
RAB21, RAB25,
RAB8A, RAD23B,
RAD51C, RAD54B,
RAF1, RAMP1,
RANBP1, RARRES2,
RAX2, RB1, RBBP4,
RBM14, RBM5, RBP1,
RBPJ, RCBTB2,
RCC1, RDX, RECQL4,
REG4, RELA, REPS1,
REST, REV1, RFC2,
RFC3, RFC4, RGS1,
RGS13, RGS16,
RGS2, RGS4, RGS5,
RHBDL2, RHOA,
RIPK1, RIT1,
RNASE4, RND2,
RNF34, RNF4,
RPL10A, RPL21P4,
RPL23, RPL23AP1,
RPL24, RPL3, RPL37,
RPL6, RPRD1B,

RPS26, RPS27L,
RPS28, RRAS,
RRBP1, RRM1,
RRM2, RRM2B,
RRP15, RSRC2,
RTKN, RTL8C,
RUNX2, S100A10,
S100A11, S100A2,
S100A3, S100A4,
S100P, SAA1, sah,
SAMHD1, SAP30,
SAT1, SCAMP3,
SCCPDH, SCD,
SCG2, SCN2B,
SCNN1A, SCNN1B,
SCUBE2, SDCCAG8,
SDHA, SDHD,
SEC14L1, SEC23A,
SEC31A, SEC31B,
SELENOP, SEMA3A,
SEPHS1, SEPHS2,
SEPTIN9, SERF2,
SERPINA5,
SERPINA7,
SERPINB2,
SERPINB4,
SERPINB5,
SERPIND1,
SERPINE2, SERPINI1,
SERTAD1, SESN1,
SESN2, SET, SETD5,
SEZ6L, SF3B3, SFN,
SFRP2, SGCE, SHH,

SHISA9, SHMT2,
SIGLEC1, SIGLEC9,
SKIC8, SLC13A5,
SLC15A1, SLC16A1,
SLC16A6, SLC22A7,
SLC22A8, SLC25A23,
SLC25A27, SLC29A1,
SLC2A1, SLC2A14,
SLC2A2, SLC2A3,
SLC35F5, SLC3A2,
SLC47A1, SLC48A1,
SLC5A1, SLC9A3,
SLCO2A1, SMAD4,
SMARCA1, SMCHD1,
SMIM10L2A, SMIM14,
SMN2, SMO, SMOX,
SMS, SMURF1,
SNAI2, SNAP29,
SNAPC1, SNHG15,
SNORD22, SNRNP48,
SNRPA1, SNTB1,
SNX6, SOAT1,
SOCS1, SOD1, SOD2,
SOX17, SOX18,
SOX2, SOX4, SP1,
SP3, SPAG5,
SPANXA1, SPANXC,
SPARC, SPATA25,
SPINK4, SPN,
SPOCK1, SPON1,
SPRR1A, SPRR1B,
SPRR3, SPRY1,
SPTAN1, SQLE, SRC,

SREBF1, SREBF2,
SRI, SRP19, SRP72,
SRP9, SRRM1,
SRSF11, SRSF6, SST,
STARD7, STAT1,
STAT3, STAU1,
STBD1, STK38, STK4,
STOM, STX5,
STXBP4, SUFU,
SUPV3L1, su(r),
SWAP70, SYCP3,
SYMPK, SYNCRIP,
SYP, SYTL2, T, TAF3,
TAF5L, TAGLN,
TANK, TAOK3, TAP1,
TAP2, TAT, TAX1BP3,
TBCD, TBCE, TBRG4,
TCF15, TCF19, TCF3,
TCF7L2, TDGF1, TEK,
TERF2, TERT,
TFCP2L1, TFDP1,
TFF1, TFPI, TFRC,
TGFA, TGFB1,
TGFB2, TGFBI,
TGFBR3, TH, THBS1,
THNSL2, THOC1,
THSD7A, TIAL1,
TIGAR, TIMELESS,
TIMP3, TINF2, TJP3,
TK1, TK2, TLR3,
TLR4, TLR9,
TMEM150C,
TMEM184B,

TMEM187, TMEM33,
TMEM40, TMEM74,
TMEM9B, TMSB10,
TMSB15A, TMSB4X,
TMSB4XP6, TNF,
TNFAIP3,
TNFRSF10A,
TNFRSF10B,
TNFRSF10C,
TNFRSF10D,
TNFRSF11B,
TNFRSF18,
TNFRSF19,
TNFRSF1A,
TNFRSF1B, TNFSF10,
TNFSF13, TNNI1,
TNNI3, TNNT1, TNS3,
TOB1, TOP1, TOP2A,
TOPBP1, TP53,
TP53AIP1, TP53I3,
TP53INP1, TP73,
TPI1, TPM1, TPMT,
TPP1, TPP2, TPST2,
TPT1, TRADD,
TRAF2, TRAP1,
TRIAP1, TRIB2,
TRIB3, TRIM2,
TRIOBP, TROAP,
Trp53, TSC2, TSHB,
TSHZ1, TSPAN8,
TSPO, TSR1, TST,
TTYH2, TUBA4B,
TUBA8, TUBB,

TUBB2A, TUBB3,
TUBB4B, TUBD1,
TXNIP, TXNRD1,
TXNRD3, TYMP,
TYRP1, U2SURP,
UBE2C, UBE2S,
UBE2T, UCHL1,
UCK2, UCP2, UGDH,
UGT1A1, UGT8,
UMPS, UNC13B,
UNC5B, UNG, ung-1,
UPP1, UPRT,
UQCR10, UQCR11,
UQCRB, UQCRC2,
UQCRFS1, UQCRH,
UROD, USH2A,
USP14, USP9X,
UTP3, UXT, VANGL2,
VASP, VAT1, VAV3,
VCL, VEGFA, VGF,
VIM, VPREB3, VPS35,
VPS37A, VPS52,
WARS1, WBP4,
WDR4, WEE1, WNT3,
WNT5A, WNT8A,
WT1, WTAP, XAF1,
XAGE1B, XBP1, XIAP,
XIST, XRCC1,
XRCC3, XRCC5,
XRCC6, YARS1,
YARS2, YBX1, YES1,
YIF1A, YWHAB,
YWHAE, YWHAH,

ZBTB18, ZBTB20,
ZBTB7A, ZC3H3,
ZCCHC18, ZDHHC9,
ZFP42, Zfp518a, ZIM2,
ZKSCAN3, ZMAT3,
ZMYND8, ZNF263,
ZNF266, ZNF282,
ZNF32, ZNF367,
ZNF408, ZNF473,
ZNF532, ZNF552,
ZNF582, ZRSR2,
ZSCAN18, PARD3B,
IRS1, MEGF11, IL11,
ALDH3A1, CYP1B1,
ITGAL, CHN2, GNAS,
TMEM167A, XRCC4,
SLC22A16, CYP19A1,
CCNK, KLC1, FGFR4,
MIR27A, PTPRC,
PON1, CBLB, DLG5,
IL6R, NOS1, ATM,
CBR1, PPP2R5D,
SELE, ABCC4, PERP,
DPYS, PIK3CA,
WNT5B, MET,
SHMT1, FOXO1,
CYP2C8, INSR,
SLC19A1, BRAF,
CYP2C19, MUC2,
REV3L, RBX1, EXO1,
ABCG1, IGFBP3,
APC, PIK3R2, IL2RA,
CYP2E1, SLCO1B1,

			GNL3, GALNT14, UPP2, PPAT, CYP1A2, CYP2C9, ABCC3, ATAD5, NR1H4, CDC21								
SB-743921	CTRP	KSP inhibitor	KIF11	Preclinical	PMID: 23394180	1.260722	NA	-0.3475159	1.22E-15	0.41608951	3.56E-08
3-Cl-AHPC	CTRP	AKT inhibitor	BCL2L1, BIRC2, CHUK, FAS, HSP90AB1, NFKBIA, RALBP1, RELA, TNFRSF10A, TNFRSF10B, XIAP	Preclinical	NA	1.028049	NA	-0.3418504	3.75E-15	0.10593642	1.35E-10

STF-31	CTRP	GLUT1 inhibitor	SLC2A4, CYP19A1, HSD3B1	Preclinical	NA	1.069723	NA	-0.3133482	7.47E-13	0.12603485	2.81E-08
parbendazole	CTRP	Tubulin inhibitor	NR1H4, AR, GMNN, CYP1A2	Preclinical	NA	0.8428409	-91.12	-0.3089235	1.62E-12	0.14344103	1.91E-07
cabazitaxel	PRISM	Microtubule inhibitor	AKT1S1, FOXO3, TUBB4A, TUBB, TUBA3C, TUBA1B, TUBA4A, TUBB4B, TUBB3, TUBB2A, TUBB8, TUBA3E, TUBA1A, TUBA1C, TUBB6, TUBB2B, TUBB1, TUB, IGF2, CYP3A4, NULL, TUBA3D, CYP3A5, CYP2C8, ABCB1, ABCG2, SLCO1B1, SLCO1B3	Phase 2	PMID: 27572899	1.061563	NA	-0.5380321	7.29E-39	0.3482832	2.42E-15
epothilone-b	PRISM	Microtubule inhibitor	CALCA, NGF, TUBB4A, TUBB, TUBA3C, TUBA1B, TUBA4A, TUBB4B, TUBB3, TUBB2A, TUBB8, TUBA3E, TUBA1A, TUBA1C, TUBB6, TUBB2B, TUBB1, CYP3A4, TUBA8	Phase 2	PMID: 21738778	1.103772	-97.63	-0.5094874	2.21E-34	0.3210276	1.92E-14
vincristine	PRISM	Tubulin inhibitor	AR, TYMS, TUBA1A, SRI, TUBB2A, ABL1, CAPG, CXCL12, TUBB4B, NRG1, DROSHA, TUBB3,	Phase 3	PMID: 28988243	1.05251	-38.68	-0.3790285	1.58E-18	0.4474621	1.23E-10

			ATRX, PIK3CA, PIK3CG, TUBA1B, TUBA4A, CP, ABCB4, VWF, SMAD3, NTF3, AFP, ABCC3, CYP3A5, NR1I2, LINC00251, TUBB8, BMP7, TUBB, DOK5, TUBB4A, GATA3, TUBB6, FGF2, TUBB1, BDNF, ABCC1, RALBP1, TUBA3E, CDKN1B, GMNN, ODC1, TUBA1C, BCR, MYCN, TUBA3C, PNPLA3, TUBA3D, TOP2A, NULL, ABCC2, CEP72, ABCC10, BCL2, TUBB2B, ACTG1, ABCB1, GSTA1, SLC22A3, ABCB11, ABCG2, SLCO1B1, SLCO1B3, CYP3A4, CYP3A7								
gemcitabine	PRISM	Ribonucleotide reductase inhibitor	XYLT2, SLC28A1, SMAD4, PTEN, IGF2, SERPINC1, ERBB2, GPX5, DAPK1, DOCK8, NT5C2, CBL, KLC3, TNF, CYP2C8, FANCC, RRM2,	Phase 4	PMID: 29949158	1.057902	-99.12	-0.3522404	4.73E-16	0.5379188	1.35E-10

			RRM1, TENT4A, KDR, PPP1R13L, RRM2B, ERCC2, AKT1, TGM2, DCK, BRCA1, SH2D5, BRCA2, EGFR, SRC, ABCG2, KRAS, ERCC1, MSH5, PPP1R18, IDH1, ATAT1, FHL2, FKBP5, CDA, PRRC2A, POLR1G, SLC29A3, NT5C1A, CDKN2A, HLA-C, POLR2A, NT5C3A, CMPK1, SLC28A3, NRG1, ETS2, CDSN, SLC28A2, ACSS2, HRAS, NT5C, NRAS, CHEK1, MUCL3, PIK3CA, HCP5, SLC29A1, TP53, CIP2A, SERPINA5, ATRX, RAF1, PSORS1C1, WEE1, ESR2, DCTD, ALG10, CASP7, C6orf15, PPCDC, XRCC1, FGFR3, CDC5L, SLC29A2, TK2, TYMS, NME1, ABCB1, ABCC10								
SGI-1776	PRISM	PIM Kinase Inhibitor	PIM1, PIM3, PIM2, FLT3, YES1, JAK2,	Preclinical	NA	1.0477	NA	-0.3269775	6.37E-14	0.3311275	1.45E-09

			HASPIN								
dolastatin-10	PRISM	Microtubule inhibitor	TUBB4A, TUBB, TUBA3C, TUBA1B, TUBA4A, TUBB4B, TUBB3, TUBB2A, TUBB8, TUBA3E, TUBA1A, TUBA1C, TUBB6, TUBB2B, TUBB1, TUB	Preclinical	PMID: 10321512	1.083865	NA	-0.3237382	1.16E-13	0.3295997	1.28E-09
echinomycin	PRISM	HIF1 Inhibitor	HIF1A, VEGFA	Preclinical	PMID: 33572152	1.049149	NA	-0.318722	2.87E-13	0.3816931	1.81E-08
MPI-0479605	PRISM	MPS1 inhibitor	TTK	Preclinical	NA	1.372288	NA	-0.3003071	7.02E-12	0.3255934	5.25E-08
